# Theoretical Study of the Iron Complexes with Aminoguanidine: Investigating Secondary Antioxidant Activity

**DOI:** 10.3390/antiox9080756

**Published:** 2020-08-15

**Authors:** Guillermo García-Díez, Nelaine Mora-Diez

**Affiliations:** Department of Chemistry, Thompson Rivers University, Kamloops, BC V2C 0C8, Canada; garciadiezg18@mytru.ca

**Keywords:** aminoguanidine, glycation inhibitor, iron complexes, copper complexes, Haber–Weiss cycle, superoxide radical anion, ascorbate, Marcus theory

## Abstract

A thorough analysis of the thermodynamic stability of various complexes of aminoguanidine (AG) with Fe(III) at a physiological pH is presented. Moreover, the secondary antioxidant activity of AG is studied with respect to its kinetic role in the Fe(III) reduction to Fe(II) when reacting with the superoxide radical anion or ascorbate. Calculations are performed at the M05(SMD)/6-311+G(d,p) level of theory. Solvent effects (water) are taken into account in both geometry optimizations and frequency calculations employing the SMD solvation method. Even though the results of this study show that AG can form an extensive number of stable complexes with Fe(III), none of these can reduce the rate constant of the initial step of the Haber–Weiss cycle when the reducing agent is $O_{2}^{•-}$. However, when the reductant is the ascorbate anion, AG is capable of reducing the rate constant of this reaction significantly, to the point of inhibiting the production of ^•^OH radicals. In fact, the most stable complex of Fe(III) with AG, having a $∆G_{f}^{{}^{\circ}}$ of −37.9 kcal/mol, can reduce the rate constant of this reaction by 7.9 × 10^5^ times. Thus, AG possesses secondary antioxidant activity relative to the Fe(III)/Fe(II) reduction with ascorbate, but not with $O_{2}^{•-}$. Similar results have also been found for AG relative to the Cu(II)/Cu(I) reduction, in agreement with experimental results.

## 1. Introduction

Sugars are necessary for life. Nevertheless, the nucleophilic attachment of different naturally occurring molecules (such as proteins, lipids or DNA) to sugars in the bloodstream may lead to the formation of harmful molecules. This natural process, known as glycation or nonenzymatic glycosylation, starts with the nucleophilic groups of these molecules attacking the carbonyl groups of the saccharides. This reaction yields a Schiff base intermediate, which undergoes a series of intramolecular rearrangements that lead to the formation of an Amadori compound. Amadori compounds may further react to form advanced glycation end-products (AGEs) [1,2]. Some of these AGEs are not known to cause any harm, while others are highly reactive, and may lead to a number of diseases, such as Alzheimer’s disease [3], eye diseases [4] or cancer, hence the need to discover novel pharmaceuticals capable of stopping this set of reactions. Glycation can be halted in three different ways: scavenging carbonyl species, scavenging radical species (which increase the formation of AGEs) or chelating metal ions, such as Cu(II) and Fe(III) [5]. These cations are essential for human metabolism. However, they can catalyze the autoxidation of Amadori compounds, hence their chelation and immobilization is of interest.

The iron-catalyzed Haber–Weiss cycle is shown in Equations (1) and (2). This process starts with the reduction of Fe(III) or Cu(II). These metals can then be oxidized again by hydrogen peroxide, resulting in the formation of very reactive ^•^OH radicals [6]. The superoxide radical anion and ascorbate are known to take part in this sequence of reactions [7,8]. A compound is said to have secondary antioxidant activity when it is capable of chelating Cu(II) or Fe(III) ions and lowering the rate constant of their reduction process. This way, the Fenton reaction (the second step of the Haber–Weiss cycle, which yields OH radicals) is hindered, and any oxidative damage caused by the radicals can be minimized. Some glycation inhibitors probably possess secondary antioxidant activity, but this has not been confirmed from the kinetic standpoint with respect to the Fe(III)/Fe(II) reduction.

Fe^3+^ + O_2_^−^ → Fe^2+^ + O_2_ (1)

Fe^2+^ + H_2_O_2_ → Fe^3+^ + OH^−^ + ^•^OH (2)

Theoretical and experimental research has been carried out in the field of glycation inhibitors. Some of the molecules studied are pyridoxamine, metformin, LR-74, tenilsetam, carnosine, pioglitazone and aminoguanidine (AG), among others [9,10,11,12]. This last compound (which was given the name pimagedine) was researched as a potential drug to treat diabetic nephropathy [13,14]. Some potential complexes between pyridoxamine, AG, ascorbic acid and a model Amadori compound with Cu(II) and Fe(III) ions have already been investigated [15,16]. In addition to this, the reactions between glycation inhibitors (namely pyridoxamine, its analogs and metformin) and several sugars (e.g., ribose and glucose) have been examined theoretically and experimentally [17,18,19,20]. The preparation of new inhibitors of AGEs has also been described in the literature [21].

Ortega-Castro et al. explored some complexes of Fe(III) with AG in water, making use of two different levels of theory: B3LYP(CPCM)/6-31+G(d) and M06(CPCM)/6-31+G(d,p). However, only one conformer of neutral AG was used as the ligand, which is not the most stable conformation, from a thermodynamic point of view, at physiological pH [15]. Four complexes were reported, two with two AG molecules (1:2 complexes) and two with three AG molecules present (1:3 complexes, both high- and low-spin complexes were optimized). The high-spin 1:3 iron complex was shown to be the most stable at the M06(CPCM)/6-31+G(d,p) level of theory, whereas the low-spin counterpart was the most stable coordination compound at the B3LYP(CPCM)/6-31+G(d) level of theory. The change in Gibbs free energy for each coordination compound was reported at the 1 atm reference state without considering pH, relative to the isolated Fe^3+^, AG and H_2_O species involved in each complex.

Ramis et al. [22] have investigated the reaction between different radicals (namely ^•^OCH_3_ and ^•^OOH) and AG in order to understand the primary antioxidant activity of this molecule (i.e., its ability to scavenge free radicals) from a thermodynamic and kinetic point of view. The level of theory used was M05-2X(SMD)/6-311+G(d,p), and the reactions were modeled in polar and nonpolar environments at physiological pH. It was found that AG is preferentially protonated under these conditions. Moreover, AG was shown to exert its primary antioxidant activity solely via hydrogen atom transfer (HAT), with larger rate constants in a nonpolar medium. According to this research, AG is only a moderate free radical scavenger. The HAT reactions between AG and the ^•^OCH_3_ radical, which were the fastest, had rate constants from 4.68 × 10^5^ to 6.40 × 10^7^ M^−1^ s^−1^. The most thermodynamically favorable reaction displayed a ΔG° of −22.4 kcal/mol.

Using an equivalent level of theory to the one applied in the previously described kinetic study (M05(SMD)/6-311+G(d,p)), our group recently performed an extensive examination of the stability of a wide range of Cu(II) complexes with AG from the thermodynamic standpoint at a physiological pH [23]. We also examined the secondary antioxidant activity of AG by means of the methodology described in [22], but adapted it to a single electron transfer (SET) reaction. We reported that AG is capable of slowing down the initial reaction of the Haber–Weiss cycle by a factor of 2.8 (from 7.71 × 10^9^ to 2.80 × 10^9^ M^−1^ s^−1^) when it chelates Cu(II) and forms the most thermodynamically stable complex. However, this was only studied with $O_{2}^{•-}$ as the reductant. The most thermodynamically stable complexes of Cu(II) with one and two ligands had standard Gibbs free energies of formation, $∆G_{f}^{{}^{\circ}}$ of −16.3 and −29.7 kcal/mol, respectively.

It is important to employ the same level of theory when studying the thermodynamic and kinetic properties of a reaction if the results are to be compared to previous research. With the goal of understanding the potential of AG as a glycation inhibitor, we are further pursuing this avenue of inquiry, consistent with the computational methods previously used [22,23]. This paper presents an in-depth study of the metal chelating activity of AG with respect to Fe(III) ions in aqueous solution at a physiological pH. Sixty complexes with one to three organic ligand molecules (1:1, 1:2 and 1:3 complexes) are reported, using both protonated and neutral AG in its four possible conformations. The most stable complexes are further investigated to quantitatively explore the secondary antioxidant activity of AG from a kinetic point of view with respect to the reduction of Fe(III) to Fe(II) ions with $O_{2}^{•-}$ and ascorbate. To the best of our knowledge, besides a recent paper from our group [24], theoretical kinetic studies of secondary antioxidant activity relative to Fe(III)/Fe(II) reduction, and its comparison with the Cu(II)/Cu(I) reduction for the same set of antioxidants, have not been previously reported.

## 2. Computational Details

Geometry optimizations and frequency calculations were carried out at the M05(SMD)/6-311+G(d,p) level of theory using the Gaussian09 software package [25]. The aqueous environment was modeled using the SMD continuum solvation method [26]. We also included the ultrafine integration grid in our calculations. It has been previously proven that the M05 hybrid meta functional accurately models the behavior of transition metals [27]. The chemistry of these elements can also theoretically be studied using the M06 hybrid meta functional, which is an improved version of the M05 functional [28]. However, the choice of the level of theory was driven by our previous studies, for consistency and to make comparisons possible [22,23].

All the relevant information of the different complexes and molecules studied can be reviewed in the Supplementary Materials (absolute standard Gibbs free energies (G°) and enthalpies (H°) at 298.15 K are reported in Table S1, alongside their Cartesian coordinates and structures). The standard Gibbs free energy change ($∆G_{f}^{{}^{\circ}}$) for the formation of each complex was calculated using Equation (3) and the corresponding G° values of reactants and products. This reaction refers to the formation of the coordination compound from its infinitely separated ligand and solvated central ion. The $∆G_{f}^{{}^{\circ}}$ was then employed to calculate the formation constant ($K_{f}$) of the complexes, following Equation (4).

(3) $$∆G_{f}^{{}^{\circ}}=\sum^{\;}G_{\mathrm{products}}^{{}^{\circ}}-\sum G_{\mathrm{reactants}}^{{}^{\circ}}$$

(4) $$K_{f}{=e}^{-\frac{∆G_{f}^{{}^{\circ}}}{\mathrm{RT}}}$$

Conventional transition state theory was used to calculate the rate constants (k) of the single electron transfer (SET) reactions in conjunction with Marcus theory [29,30]. When rate constants were in the diffuse-limited regime (*k* > 1 × 10^8^ M^−1^ s^−1^), apparent rate constants, $k_{app}$, were calculated by applying the Kimball-Collins theory [31]. This model requires the calculation of the steady-state Smoluchowski rate constant for an irreversible diffusion-controlled bimolecular reaction [32], and the application of the Stokes–Einstein approach [33,34]. Details on this methodology and the equations used can be found in previous publications [35,36,37,38,39]. This methodology has been successfully used in studies of primary and secondary antioxidant activity [22,23,24,35,36,37,38,39,40].

Since the calculated complexes, both in high- and low-spin versions, contain a metallic center with unpaired electrons, it is important to determine whether these compounds present any spin contamination before and after annihilation. This effect arises due to the merging of different electronic spin states, and could affect the calculated energies and/or geometries [41]. Table S2 displays the $\langle {\hat{S}}^{2}\rangle$ values of the calculated Fe(III) and Fe(II) complexes before and after annihilation where applicable. These values were checked to ensure spin contamination was negligible (values close to 0.75, 6.00 and 8.75, for systems with two, four and five unpaired electrons, respectively). This was not the case for the low-spin complexes identified as {6} and {79} that do not play a relevant role in this work.

Ascorbic acid presents two chiral centers, thus having four possible stereoisomers. The L isomer was used, as this is the naturally occurring molecule. The hydroxyl group at the 4-position in the furan ring is deprotonated under physiological pH conditions. The structure of ascorbate (ASC^−^) is labelled {90} in the Supplementary Materials.

## 3. Results and Discussion

Since the aqueous pK_a_ of AG is 11.5 at 298.15 K, it is mostly protonated (AGH^+^) at physiological pH (7.40) [42]. Nevertheless, complexes were studied both with protonated and neutral AG, while accounting for the pH conditions to compare their stabilities. Most Fe(III) complexes have octahedral geometry and we have followed this information when exploring various geometrical possibilities. However, it is known that Fe(III) complexes can exhibit other geometries [43], some of which have been calculated previously [15]. Thus, some complexes with coordination numbers of four and five were also explored. Furthermore, high- and low-spin complexes were also taken into account.

### 3.1. Complexes of Fe(III) with Protonated AG

**Figure 1:**
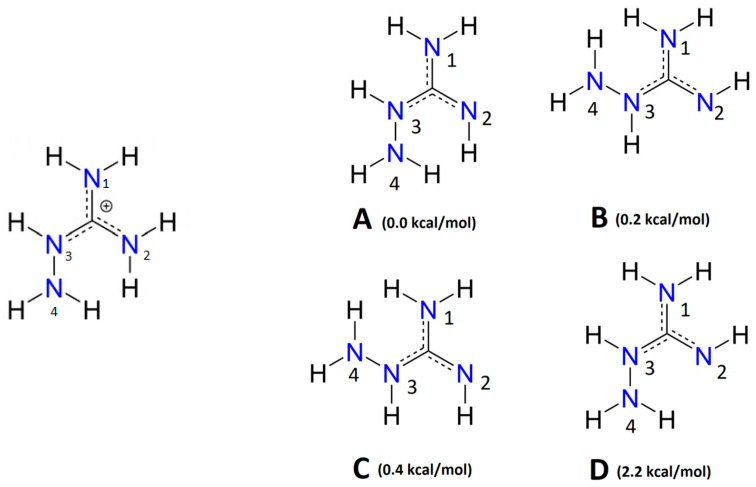
Forms of aminoguanidine (AG) considered in this study (on the left, protonated AG, AGH^+^; on the right, the four conformers of AG in stability order).

As shown in Figure 1, protonated AG, labeled AGH, exists in one conformation with two possible coordinating nitrogen atoms: N_2_ and N_4_. Ten complexes containing one, two or three AGH ligands were optimized, all of which were endergonic. The corresponding thermodynamic data for each complex ($∆G_{f}^{{}^{\circ}}$, $K_{f}$ and ${\mathrm{logK}}_{f}$) following the formation equilibrium shown in Equation (5), are displayed in Table S3. The structures of these complexes (which include coordinating bond distances) can be found in Figure S1.

(5) $$x{\mathrm{AGH}}^{+}\;+\;[\mathrm{Fe}(H_{2}O{)}_{6}]3^{+}\;⇆\;{\left[\mathrm{Fe}(\mathrm{AGH}{)}_{x}(H_{2}O{)}_{n}\right]}^{(3+x)+}\;+\;(6-n)H_{2}O\;∆G_{f}^{{}^{\circ}}{}_{Fe^{3+}-AGH^{+}},\;K_{f_{Fe^{3+}-AGH^{+}}}$$

The lowest Gibbs free energy dimer or trimer between AGH ligands was used when taking into account the reactant species in the formation reaction of the complexes. The same approach was employed when considering the cluster of (6 − n) water molecules, with *n* = 0.5 depending on the coordination of AGH^+^ to Fe(III). In this approach, no corrections due to changing the reference state or to account for the solvent cage effect are needed because the same number of species exists in the reactant and product sides. The same idea is followed for each of the complex formation equilibria considered in this study, consistent with our previous work [23,24,25]. For additional explanations on these corrections and our procedure, refer to [39] and Appendix 1 in the Supplementary Materials.

Four pairs of high- (hs) and low-spin (ls) complexes were calculated. In all cases, the low-spin complexes ({2}, {4}, {6} and {10}) were significantly less stable than their high-spin counterparts. Fe(III) is known to usually form high-spin complexes except with strong field ligands, with which it prefers to form low-spin complexes. Therefore, AGH is not a strong enough ligand to stabilize the Fe(III) low-spin complexes over the high-spin ones. It is interesting to note that the low-spin complexes always have shorter bond distances from the coordinating atoms to the central ion compared to the high-spin counterparts, especially from the water molecules. This behavior has been shown previously in computation calculations with other ligands [44].

Regarding the high-spin complexes, a trend can be observed: the fewer the number of AGH ligands in the complex, the more stable it becomes. Hence, stability increases in the order {1} < {3} < {5}. This observation can be rationalized by considering the loss of planarity and resonance suffered when AGH coordinates through N_2_, which reduces the stability of the complex. When N_2_ becomes a coordination point, the hydrogens have to move in order to adapt to the new geometry. Thus, the guanidine moiety is not planar anymore. In addition, the electron pair that this nitrogen can donate to the delocalized system now plays a role in the interaction between the ligand and the metal center. Hence, the loss of resonance. Moreover, complexes such as {1} contain a great amount of positive charges close together, which could lead to electrostatic repulsion, further reducing the stability. It is then not unexpected that the most stable complex with AGH is the one where only one AGH coordinates to Fe(III), and less positive charges are in close proximity in a monodentate fashion through N_4_ (so that the AGH planarity or resonance is not lost, as it is not part of the guanidine moiety) complex {7}. Complex {8}, with two monodentate AGH ligands with N_4_ coordination, follows in stability.

Complexes {9} and {10} were optimized with coordination number four. The high-spin complex is the second least stable among the high-spin complexes, more so than the six-coordinated equivalent 1:2 complex {3}. This indicates that Fe(III) prefers a six-coordinated environment over a four-coordinated one.

### 3.2. Complexes of Fe(III) with AG

Depending on the conformation of the hydrazine moiety and the imine hydrogens, unprotonated AG can exist in four conformations, as shown in Figure 1. These conformations, labeled AG_A_, AG_B_, AG_C_ and AG_D_, have been energetically ordered. The nitrogen atoms are numbered as well to easily indicate which nitrogen coordinates to the iron center in the complex. AG_A_ is the most stable conformation of AG. However, only some complexes between the AG_D_ conformer and Fe(III) were examined in [15].

Various combinations of 1:3, 1:2 and 1:1 octahedral complexes of Fe(III) with AG were calculated, together with several penta- and tetra-coordinated complexes for a total of fifty coordination compounds. Table S4 displays their $∆G_{f}^{{}^{\circ}}$, $K_{f}$ and ${\mathrm{logK}}_{f}$ values relative to the formation equilibrium illustrated in Equation (5), where the reactant xAG (with *x* = 2, 3) refers to the lowest Gibbs free energy dimer or trimer of AG_A_, the most stable conformation of AG.

**Figure 2:**
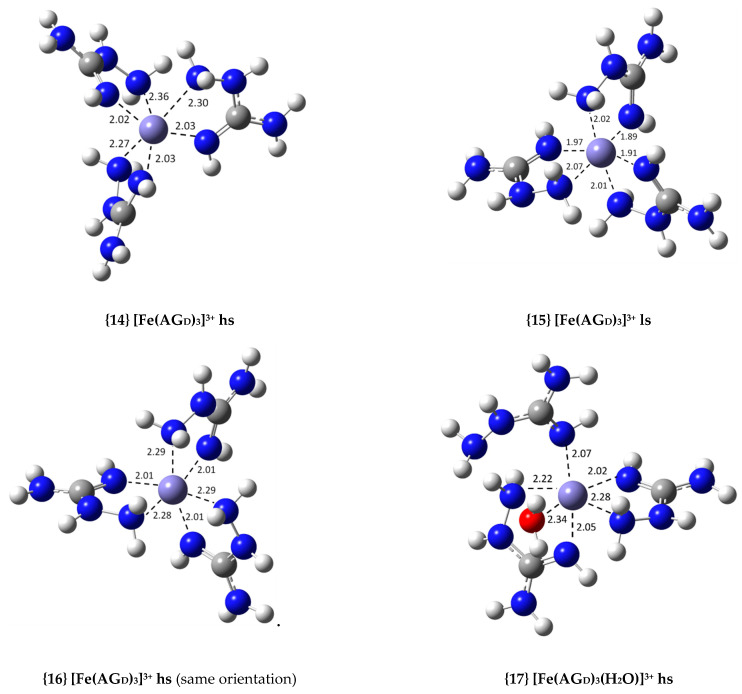

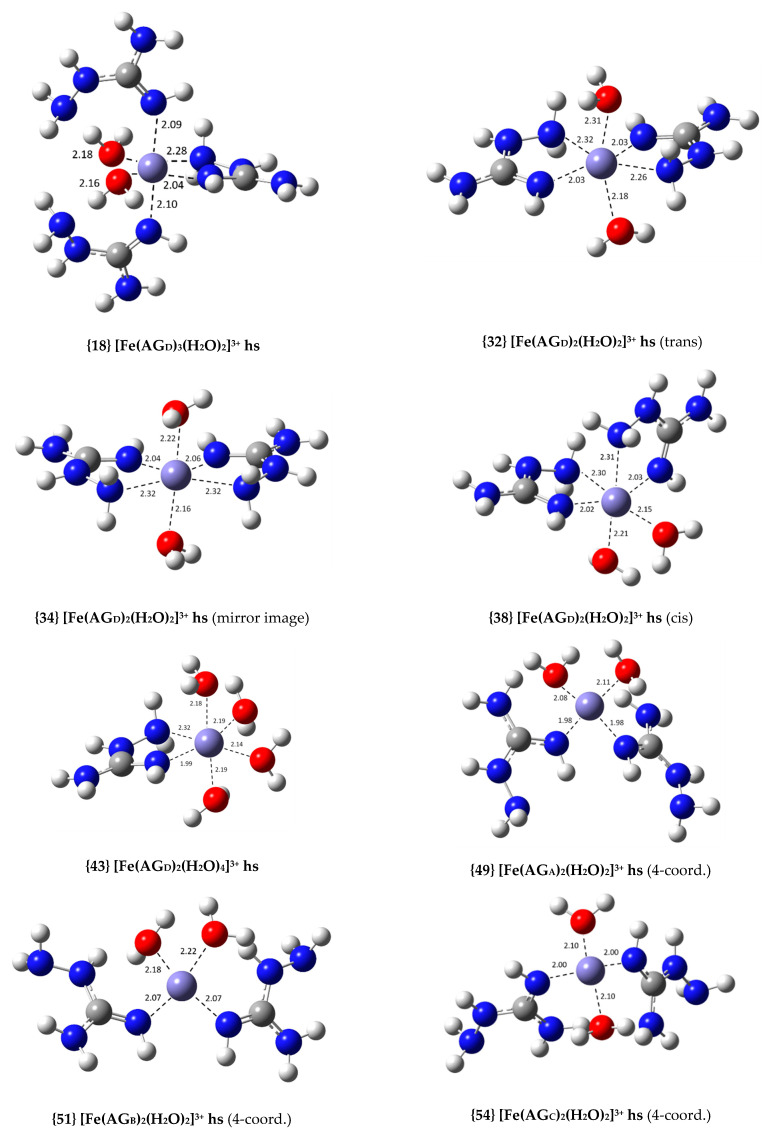

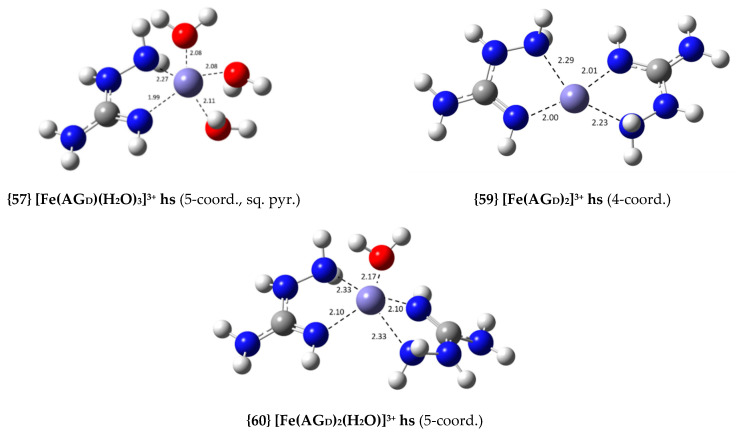
Optimized geometries of the most relevant Fe(III) complexes with AG displayed in Table 1 (bond distances in Å); high- (hs) and low-spin (ls) complexes are identified.

As previously illustrated in our study of Cu(II) complexes with AG [23], transforming Equation (6) into Equation (7) implies adding 5.6 kcal/mol to the $∆G_{f}^{{}^{\circ}}$ values reported in Table S4 for each AG ligand present in the complex. This way, the Gibbs free energy cost of forming AG from AGH^+^ under physiological pH conditions is taken into account. The new values, displayed in Table S5 for the fifty complexes calculated, can be properly compared to the values reported in Table S3, relative to an equivalent set of reactant species. At physiological pH conditions, the thermodynamic stability of all complexes was reduced. Even though two of the complexes became endergonic (complexes {36} and {48}), the most stable ones remained highly exergonic. Table 1 displays the thermodynamic calculations for the most relevant Fe(III) complexes with AG. The structures of these complexes are shown in Figure 2, with indicated coordinating bond distances. The structures of the remaining complexes are displayed in Figure S2.

(6) $$x\mathrm{AG}+{\left[\mathrm{Fe}{\left(H_{2}O\right)}_{6}\right]}^{3+}\;⇆\;{\left[\mathrm{Fe}{\left(\mathrm{AG}\right)}_{x}{\left(H_{2}O\right)}_{n}\right]}^{3+}\;+\;(6-n)H_{2}O\;∆G_{f}^{{}^{\circ}}{}_{{\mathrm{Fe}}^{3+}-\mathrm{AG}}{,\;K}_{f_{{\mathrm{Fe}}^{3+}-\mathrm{AG}}}$$

(7) $$x{\mathrm{AGH}}^{+}+{\left[\mathrm{Fe}{\left(H_{2}O\right)}_{6}\right]}^{3+}\;⇆\;{\left[\mathrm{Fe}{\left(\mathrm{AG}\right)}_{x}{\left(H_{2}O\right)}_{n}\right]}^{3+}\;+\;(6-n)H_{2}O\;+\;{\mathrm{xH}}^{+}$$

The relative distribution of two and three AG molecules coordinating the Fe(III) central ion lead to different structures being optimized for the complexes and various labels have been used to differentiate them. For example, in a 1:3 complex, three bidentate AG molecules could be equally oriented (or heading in the same direction), e.g., {14}, or not, e.g., {16} (see Figure 2). The label “same orientation” has been used to distinguish complexes in this situation. The labels “mirror image”, “cis” and “trans” have been used to identify pairs of related 1:2 complexes with the same type and number of coordination sites, but different ligand distributions, e.g., see complexes {32}, {34} and {38} in Figure 2. These differences in spatial distribution usually led to $∆G_{f}^{{}^{\circ}}$ values within 0.5 to 3.0 kcal/mol of each other.

High- and low-spin complexes were considered. Of the fifty Fe(III)-AG complexes calculated, ten ({15}, {19}, {20}, {23}, {25}, {28}, {30}, {33}, {35}, {37}) were low-spin versions. In all cases, as previously found with AGH^+^ as a ligand, the low-spin complexes were significantly less stable than their high-spin counterparts. Only complex {15} is exergonic, but 30 kcal/mol less stable than its high-spin counterpart, complex {14}. For all other comparisons (1:2 complexes), differences of about 40 kcal/mol were calculated. Furthermore, as previously reported, the bond distances from the coordinating atoms to the central ion are shorter when compared to the equivalent high-spin complexes.

The only 1:3 complexes with AG as bidentate ligands that are exergonic are those with AG_D_, complexes {14} and {16}, with $∆G_{f}^{{}^{\circ}}$ values of −36.8 and −37.9 kcal/mol, respectively. These are the most thermodynamically stable Fe(III) complexes with AG. Unlike the three other conformations of AG, AG_D_ can coordinate through the most negatively charged nitrogen atom without losing planarity or resonance upon coordination, leading to more thermodynamically stable complexes. In contrast with AGH+, N_2_ in AG_D_ is a neutral imine. This atom can coordinate to the metal center and retain the planarity of the guanidine moiety as well as its resonance. The stability of this conformation decreases if the ligands become monodentate and water fills the vacant coordination site. This behavior is exemplified by complexes {17} and {18}. If one of the AG_D_ ligands becomes monodentate, the stability drops by more than 10 kcal/mol. The stability drops another 5 kcal/mol if a second AG_D_ becomes monodentate.

When considering the octahedral 1:2 complexes, those that contain two bidentate AG_D_ ligands are the most exergonic, as expected. These are complexes {32}, {34} and {38}, with $∆G_{f}^{{}^{\circ}}$ values of −20.1, −19.0 and −22.9 kcal/mol, respectively. As the AG_D_ ligands go from bidentate to monodentate (see complexes {39} and {40}), their stability drops dramatically to $∆G_{f}^{{}^{\circ}}$ values of −13.5 and −4.1 kcal/mol, respectively. This shows that AG_D_ is a much more effective bidentate ligand. Mixing AG_D_ and other AG conformers, e.g., AG_C_ in {36}, both as bidentate ligands, produces a complex with intermediate stability between that of the complexes where only one of the conformers is present, {27} and {32}. In addition, complexes which present other conformers of AG are exergonic as long as these are monodentate, as in the case of complexes {21}, {26} and {31}.

Two sets of 1:1 complexes were optimized with the different conformations of AG, one group with AG acting as a bidentate ligand (complexes {41} to {43}; no alternative arrangement of the ligands is possible) and another group with AG as a monodentate ligand (complexes {44} to {47}). The complexes containing AG_B_ and AG_C_ are endergonic (complexes {41} and {42}), while complex {43}, with AG_D_ as a ligand, shows the highest stability in this group, with a $∆G_{f}^{{}^{\circ}}$ of −10.3 kcal/mol. The 1:1 monodentate complexes {44} to {47} have AG coordinating via N_2_, the nitrogen with the highest negative charge. These complexes are all exergonic (with $∆G_{f}^{{}^{\circ}}$ values between −5.5 and −2.9 kcal/mol), for planarity and resonance are maintained in the AG ligand. The complex containing AG_D_ is the least stable, {47}, which reinforces our previous observation.

There are experimental studies with copper and AG which indicate that this transition metal prefers a square planar geometry [45]. No experiments of this kind have been done with Fe(III) and AG, and thus the preferred geometry of this metal when coordinating to AG is unknown. Therefore, it is of interest to study other possible geometries besides the octahedral one. Thirteen non-octahedral complexes were calculated, {48} to {60}, and the structures of the most relevant complexes are displayed in Figure 2 (the rest can be found in Figure S2 in the Supplementary Materials). Nine of the eleven exergonic non-octahedral complexes calculated have $∆G_{f}^{{}^{\circ}}$ values below −10 kcal/mol. Some of these complexes were originally designed as octahedral complexes, but they lost coordination points during the optimization process. This is the case of complexes {48}, {49}, {50} and {53}. Except for complex {53}, all the aforementioned complexes contain AG_A_ as the ligand. As AG_A_ can only coordinate through N_1_ and/or N_2_, the resulting complexes present high-angle strain (a four-membered ring is formed between the ligand and the Fe(III) ion). It is then not surprising that some of the ligands became monodentate and a lower coordination number was attained during the optimization. Of these, complexes {49} and {50} are quite stable, and so it was decided to optimize the analogous complexes with AG_B_ and AG_C_. The resulting complexes ({51}, {52}, {54} and {55}) show stabilities akin to those of {49} and {50}. Among these, the 4-coordinated complexes, {49}, {51} and {54}, are the most stable, with $∆G_{f}^{{}^{\circ}}$ values of −19.6, −21.6 and −20.5 kcal/mol, respectively, with stabilities comparable to that of {38} (the most stable octahedral 1:2 complex with AG_D_). The planarity and resonance of the AG ligands is preserved, and these only coordinate to the central ion by N_2_, hence, the increased stability when compared to the bidentate counterparts. In the case of AG_D_, we decided to optimize complexes with coordination numbers lower than six in which the ligands were bidentate, as this is favored by this conformer of AG. Three of these, {56}, {57} and {58}, are 1:1 complexes and two, {59} and {60}, are 1:2 complexes. Of these five, the most stable are the 1:2 complexes, especially the penta-coordinated one {60}. Actually, this complex is the most stable of all the 1:2 AG_D_ complexes, slightly more so than {38}. Only the 1:3 fully bidentate AG_D_-containing complexes surpass the stability of this complex. The same is true for the 1:1 complexes. Both {56} and {57}, with $∆G_{f}^{{}^{\circ}}$ values of −10.8 and −15.3 kcal/mol, respectively, are more stable than the corresponding octahedral complex {43} with a $∆G_{f}^{{}^{\circ}}$ of −10.3 kcal/mol. It appears that Fe(III) favors lower coordination numbers when bonded to fewer than three AG_D_ ligands. Nonetheless, as AG_D_ prefers to be bidentate, the 1:3 complex has to be octahedral. These results need experimental research to be confirmed.

All the calculated complexes with protonated or neutral AG with one, two or three ligands, in which any of the organic ligands is bidentate, have these ligands forming a five-membered ring with the central ion. An exception to this is found when AG_A_ is the bidentate ligand and a four-membered ring is formed. These complexes are endergonic, e.g., complexes {19}, {20} and {48}, see Figure S2.

Some insightful similarities can be drawn when comparing the thermodynamic stability of the Fe(III) complexes with AG with that of equivalent Cu(II) complexes previously reported [23]. The most stable complexes are those containing the highest number of bidentate AG_D_ ligands. In general, other conformers of AG do not form stable complexes except when the ligands are monodentate, but these complexes are not particularly stable, either. Moreover, even though AG is protonated at physiological pH, AGH^+^ cannot form stable complexes with Cu(II) or Fe(III). When homologous complexes are compared, the Cu(II)-AG complexes tend to be more stable than the Fe(III)-AG complexes. For example, the most sTable 1:2 complex of Cu(II) has a $∆G_{f}^{{}^{\circ}}$ value of −29.7 kcal/mol, whereas the most sTable 1:2 complex of Fe(III), {60}, has a $∆G_{f}^{{}^{\circ}}$ of −23.5 kcal/mol. When considering the most sTable 1:1 complexes, the Cu(II) complex with a $∆G_{f}^{{}^{\circ}}$ of −16.3 kcal/mol is also more stable than the Fe(III) complex, {57}, with a $∆G_{f}^{{}^{\circ}}$ of −15.3 kcal/mol. It is interesting to note that the opposite situation was found when comparing the M06(SMD)/6-31+G(d,p) thermodynamic stability of 1:1 Cu(II) and Fe(III) complexes with lipoic (LA) and dihydrolipoic (DHLA) acids [24]. The Cu(II) complexes were found to be about 6 kcal/mol less stable than their Fe(III) counterparts. LA and DHLA coordinate via the carboxylate oxygen atoms and –SH (or –S^−^) groups, while AG coordinates solely via nitrogen atoms.

The previous study of AG complexes with Fe(III) reported the optimization of four complexes at the M06(CPCM)/6-31+G(d,p) and B3LYP(CPCM)/6-31+G(d) levels of theory [15]. The four reported complexes match our complexes {14}, {15}, {32} and {59}. Nonetheless, their $∆G_{f}^{{}^{\circ}}$ calculations (directly related to the stability constants of these complexes) differ significantly from ours. Whereas the $∆G_{f}^{{}^{\circ}}$ values of our 1:3 bidentate complexes {14} and {15} are −36.8 and −6.7 kcal/mol, respectively, they obtained much lower values with both levels of theory: −114.9 and −96.6 kcal/mol (M06(CPCM)/6-31+G(d,p)), and −115.3 and −116.4 kcal/mol (B3LYP(CPCM)/6-31+G(d)), respectively. The M06(CPCM) high- and low-spin stability difference (18.3 kcal/mol) is significantly less than in our current study (30.1 kcal/mol). At B3LYP(CPCM), the high- and low-spin stability difference of 1.1 kcal/mol favors the low-spin complex. A similar situation arises with complexes {32} and {59}, for which our $∆G_{f}^{{}^{\circ}}$ values are −20.1 and −20.5 kcal/mol, respectively. On the other hand, Ortega-Castro et al. reported values of −78.8 and −72.0 kcal/mol (M06(CPCM)/6-31+G(d,p)), and −80.1 and −85.0 kcal/mol (B3LYP(CPCM)/6-31+G(d)), respectively. Based on this previous study [15], complex {14} would be the most stable complex at the M06(CPCM)/6-31+G(d,p) level of theory (this would overlap with our results if we ignore complex {16}, which is closely related to {14}). Nonetheless, at the B3LYP(CPCM)/6-31+G(d) level of theory, complex {15} is the most stable of the four. It should be noted that this group obtained the $∆G_{f}^{{}^{\circ}}$ values by subtracting from the standard Gibbs free energy of the complexes the standard Gibbs free energy of the isolated Fe^3+^, AG (using AG_D_, which is not the most stable form of AG in aqueous solution) and H_2_O species, without making reference state conversions (the calculated $∆G_{f}^{{}^{\circ}}$ was reported at the 1 atm reference state). Furthermore, since pH was not considered in the previous calculations, the deprotonation energy of AG was not taken into account. Given that this group also researched the stability of various Fe(III) complexes with ascorbate, a model Amadori compound, pyridoxamine and LR-74 (a novel glycation inhibitor), we believe it would be appropriate to perform these calculations again, making use of the methodology we have applied.

### 3.3. Kinetic Calculations for the Reduction of Fe(III): Comparison with the Cu(II)/Cu(I) Reduction

As previously stated, AG would exhibit secondary antioxidant activity if it could chelate Fe(III) and reduce the rate constant of its reduction with $O_{2}^{•-}$ or with ascorbate (${\mathrm{ASC}}^{-}$), which constitutes the first step of the Haber–Weiss cycle (shown in Equation (8). In doing so, AG would minimize (or perhaps fully inhibit) the formation of ^•^OH radicals in the second step, which are very harmful species. A similar set of reactions would also apply to the reduction of Cu(II) to Cu(I) [23].

(8) $${\left[\mathrm{Fe}{\left(H_{2}O\right)}_{6}\right]}^{3+}{+\;\mathrm{ASC}}^{-}\to {\left[\mathrm{Fe}{\left(H_{2}O\right)}_{6}\right]}^{2+}{+\mathrm{ASC}}^{•}$$

$${\left[\mathrm{Fe}{\left(H_{2}O\right)}_{6}\right]}^{2+}{+\;H}_{2}O_{2}\to {\left[\mathrm{Fe}{\left(H_{2}O\right)}_{6}\right]}^{3+}{+\mathrm{OH}}^{-}{+\mathrm{OH}}^{•}$$

It is well known that ascorbate undergoes oxidation in the presence of metal ions, such as Cu(II) and Fe(III) [46], and that the reduced metal ions can lead to the formation of ^•^OH radicals. Nonetheless, it has been shown experimentally that the rate of oxidation of ascorbate is reduced when copper is coordinated to AG, among other glycation inhibitors [9,21]. It is thus of interest to study from a theoretical perspective whether this holds true for the Cu(II) and Fe(III) sets of complexes, using our methodology. In addition to this, some organic molecules have been evaluated as potential secondary antioxidants towards the reduction reaction of Cu(II) with $O_{2}^{•-}$ and ${\mathrm{ASC}}^{-}$. These studies are of a theoretical nature and include the calculation of the rate constants of the resulting reactions [23,35,47,48,49]. Nevertheless, to the best of our knowledge, besides a recent paper from our group [24], this same type of theoretical investigation has not been carried out for the analogous Fe(III)/Fe(II) reduction, and no comparison has been made to the Cu(II)/Cu(I) system for the same set of antioxidants. But this is a relevant topic which has been explored experimentally for various compounds [50,51,52,53,54].

In order to study the secondary antioxidant activity of AG relative to the Fe(III)/Fe(II) reduction, the most stable Fe(III) complexes of each set were considered. The analogous high-spin Fe(II) complexes were optimized using, as a starting point, the corresponding Fe(III) complexes, and the rate constant for each reduction process was calculated as previously described. The 1:3 low-spin exergonic complex {15} was also considered; its low-spin Fe(II) reduction complex is {61}.

**Figure 3:**
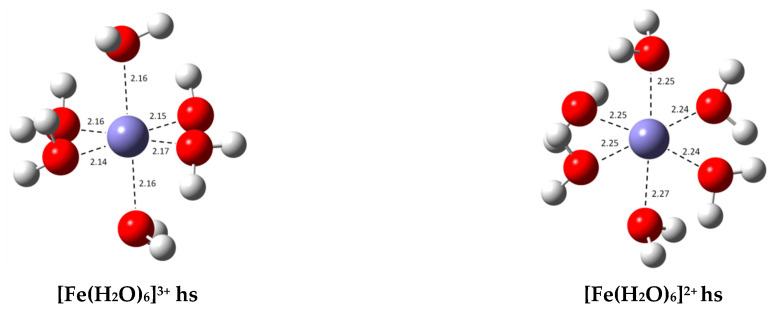
Optimized geometries of the most stable hydrated Fe(III) and Fe(II) complexes in aqueous solution (bond distances in Å); both complexes are high spin (hs).

Kinetic calculations were performed on seventeen single electron transfer (SET) reactions with both $O_{2}^{•-}$ and ${\mathrm{ASC}}^{-}$ as reducing agents. Kinetic and thermodynamic information for each set of reactions (*k*, *k_D_*, *k_app_*, ΔG^0^ and ΔG^≠^) can be found in Table S6 (reactions with $O_{2}^{•-}$) and Table S7 (reactions with ${\mathrm{ASC}}^{-}$). Fourteen of these reactions involve the complexes listed in Table 1. We were unable to include the SET reaction from complex {18} because it was not possible to optimize the geometry of its Fe(II) complex. For comparison purposes, the rate constant for the reduction of the hydrated complex from ${\left[\mathrm{Fe}{\left(H_{2}O\right)}_{6}\right]}^{3+}$ to ${\left[\mathrm{Fe}{\left(H_{2}O\right)}_{6}\right]}^{2+}$, the reference reaction, was also calculated in both cases. These structures are displayed in Figure 3.

**Figure 4:**
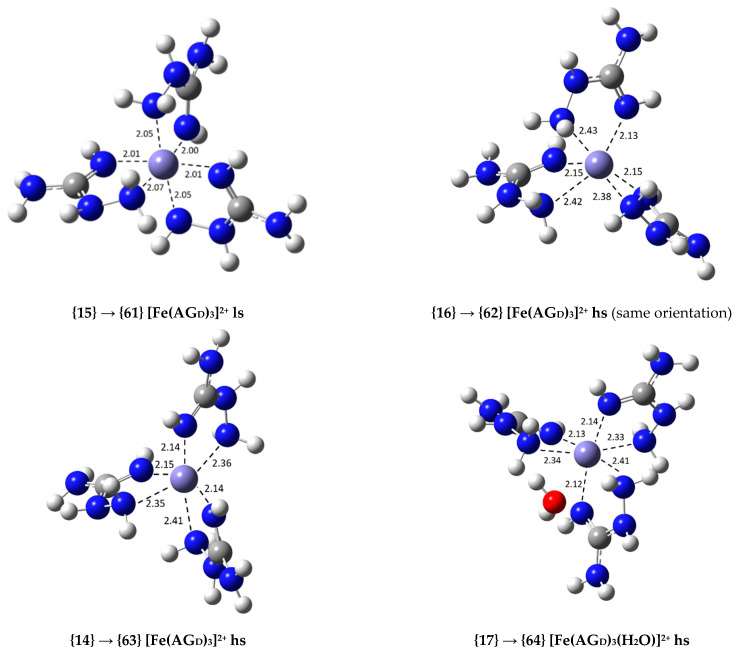
Optimized geometries of some of the Fe(II) complexes with AG in aqueous solution (indicating the Fe(III) complex used as the starting point in each case; bond distances in Å); high- (hs) and low-spin (ls) complexes are identified.

The reactions have been ordered in ascending value of *k* (and *k_app_*) relative to the reaction with ${\mathrm{ASC}}^{-}$. More compact kinetic information is reported in Table 2, including the rate constant ratio relative to the reference reaction. Since the calculated *k* values, including those of the reference reactions, were larger than 1.0 × 10^8^ in all cases, except for three reactions with ${\mathrm{ASC}}^{-}$, diffusion corrections were applied, which led to apparent rate constants in the order of 10^8^–10^9^. The structures of four of the calculated Fe(II) complexes that appear in Table 2, {61}, {62}, {63} and {64}, are shown in Figure 4. The original Fe(III) complex has been indicated for each reduced counterpart. Additional structures are displayed in Figure S3. The Fe(II) complexes have longer coordinating bond lengths than those of their corresponding Fe(III) complexes.

None of the Fe(III) complexes investigated are capable of reducing the rate constant of the reference reaction with $O_{2}^{•-}$ (7.28 × 10^9^ M^−1^ s^−1^), either before and after applying diffusion corrections. Thus, AG does not possess secondary antioxidant activity relative to the Fe(III)/Fe(II) reduction when reacting with the superoxide radical anion. Moreover, no clear trend relates the thermodynamic stability of the Fe(III) complexes to their SET rate constants (see Figure S4). The same is observed when inspecting the results of the Cu(II) complexes in Figure S5. Complex {16} is the most thermodynamically stable complex between AG and Fe(III), but its reaction with $O_{2}^{•-}$ has the third largest *k_app_* value of all those studied.

The results recently published for the Cu(II)/Cu(I) reduction with $O_{2}^{•-}$ at the same level of theory are somewhat similar to the new kinetic results reported for the Fe(III)/Fe(II) reduction [23]. The reference reaction, with a calculated *k_app_* of 7.71 × 10^9^ M^−1^ s^−1^ (which is in excellent agreement with the experimental value of (8.1 ± 0.5) × 10^9^ M^–1^ s^–1^) [55], was only reduced by 1.1 to 2.8 times (which is not significant) in three of the twelve reactions studied. Consequently, no significant secondary antioxidant activity was reported for AG relative to the Cu(II)/Cu(I) reduction with $O_{2}^{•-}.$

The kinetic results relative to the Fe(III)/Fe(II) reduction with ${\mathrm{ASC}}^{-}$ are quite different from those obtained with $O_{2}^{•-}$ All the Fe(III) complexes studied are able to reduce the rate constant of the reference reaction with ${\mathrm{ASC}}^{-}$ (7.43 × 10^9^ M^−1^ s^−1^). Even though most of the considered Fe(III) complexes only reduce this rate constant by a negligible/modest amount (1.01 to 7.6 times, see Table S7), complexes {15}, {16} and {14}, whose reduction products are displayed in Figure 3, can reduce it by 1.7 × 10^9^, 7.9 × 10^5^ and 3.8 × 10^4^ times, respectively. These are drastic rate constant reductions able to inhibit the production of ^•^OH radicals due to the Fenton reaction.

Complexes {14} and {16} are by far the most stable complexes of Fe(III) and AG, and {38} is the most stable octahedral 1:2 complex. However, {60}, the most sTable 1:2 complex overall, leads to a SET reaction that is only 2.1 times slower than the reference reaction. The situation with {15} is quite interesting. This is the only low-spin complex of AG_D_ which is slightly exergonic (see Table 1). Its relative instability is not unexpected, as the free Fe(III) aquo-complex is high spin, and the change in spin entails a large energy increase. Nonetheless, it is capable of reducing the rate constant of the reference reaction the most. It appears that the spin state of the metal center may play a role in this reaction. Not much research has been done to understand how the chelation of Fe(III) affects the rate constant of the reduction reaction with ascorbate. Nonetheless, some experimental studies point to a reduction in the value of *k* [9], which is confirmed by our calculations.

Our previous study of the AG complexes with Cu(II) reports kinetic calculations relative to the Cu(II)/Cu(I) reduction with $O_{2}^{•-}$ Identifying the copper complexes with the labels introduced in [23] and ordering the reactions as done previously (see Tables S2 and S4 in [23]), Table S8 reports equivalent thermodynamic and kinetic calculations for the SET reactions with ${\mathrm{ASC}}^{-}$. More compact kinetic information for five of the twelve reactions is reported in Table 2.

Once again, strong similarities are observed between the reduction reactions of the Cu(II) and Fe(III) complexes with ${\mathrm{ASC}}^{-}.$ Excluding the reaction of the endergonic complex {4} with protonated AG, the other reactions studied have *k* or *k_app_* values that are lower than the *k_app_* of the reference reaction (2.1 × 10^9^ M^−1^ s^−1^). More than half of these reactions have *k* values below 1.0 × 10^8^ M^−1^ s^−1^ and required no diffusion corrections. The greatest rate constant reductions relative to the reference reaction are obtained from complexes {11} (4.4 × 10^3^ times), {29} (2.6 × 10^4^ times), {25} (5.9 × 10^4^ times), {23} (3.2 × 10^7^ times) and {24} (3.5 × 10^7^ times): see Table 2. These five complexes all contain AG_D_ as a ligand. The last two are definitely able to fully inhibit the formation of ^•^OH radicals. There is no Cu(II)/Cu(I) reaction equivalent to the low-spin case previously reported for the Fe(III)/Fe(II) reduction with ${\mathrm{ASC}}^{-}.$

Complex {11} is a bidentate 1:1 Cu(II) complex with AG_D_. Further reduction of *k* can be observed when a second bidentate ligand is added. Adding an AG molecule other than AG_D_ (or adding AG_D_ in a monodentate fashion) results in a moderate *k* reduction (as seen with complexes {29} and {25}). A remarkable *k* reduction can be observed when two AG_D_ molecules coordinate in a bidentate fashion to Cu(II). Both complexes {23} and {24} can reduce the value of *k* more than 10^7^ times. Not surprisingly, these are the two most thermodynamically stable Cu(II) complexes calculated, and high concentrations of AG would be required to favor their biochemical formation [23]. These results, and the equivalent ones for the Fe(III)/Fe(II) reduction, show that AG indeed has secondary antioxidant activity when the reductant is ascorbate. This fact is in accordance with previously mentioned experimental work for the Cu(II) complexes with AG [9,21].

The Cu(II)/Cu(I) reduction reactions with $O_{2}^{•-}$ lead to slightly greater rate constant reductions relative to the reference reaction, when compared with the Fe(III)/Fe(II) reduction reactions (see Table 2), but they are smaller with ${\mathrm{ASC}}^{-}.$ In general, in the four groups of reactions studied, the more exergonic the SET reaction, the smaller its ΔG^≠^ value and the larger its rate constant. The SET reactions with ascorbate usually have less negative ΔG^0^ values, and for those with the smallest rate constants (with the largest positive ΔG^0^ values), the values of ΔG^0^ and ΔG^≠^ are very similar. This was also observed when investigating the secondary antioxidant activity of dihydrolipoic acid relative to the Fe(III)/Fe(II) reduction with ${\mathrm{ASC}}^{-}$ [24]. Finally, we have also plotted the $∆G_{f}^{{}^{\circ}}$ values of each complex with the rate constant of the SET reaction in which the complex participates. These plots can be found in Figures S4 and S5 in the Supplementary Materials. Interestingly, for the SET reactions of Cu(II) complexes with $O_{2}^{•-}$ and ${\mathrm{ASC}}^{-},$ the most stable complexes are the ones that slow the reactions the most. This holds true for the reaction of the Fe(III) complexes with ascorbate, but not with $O_{2}^{•-}$. As previously stated, there seems to be no correlation between the $∆G_{f}^{{}^{\circ}}$ of an Fe(III)-AG coordination compound and the rate constant of the reduction reaction with $O_{2}^{•-}$, but there is in the rest of the cases.

## 4. Conclusions

We have been able to demonstrate, from a theoretical chemistry standpoint, that AG can coordinate to Fe(III) to form many stable complexes with a variety of coordination numbers (4, 5 and 6). Furthermore, the calculations take into account aqueous conditions and physiological pH. This molecule tends to be protonated under physiological conditions, for the pK_a_ of AG is 11.5. Nonetheless, only the deprotonated version of this molecule is capable of forming stable complexes. Moreover, of the four conformers studied, AG_D_ (the least stable conformer of AG) forms the most stable complexes. An increase in stability is seen as bidentate AG_D_ ligands are added to the complex. For 1:1 and 1:2 complexes with AG, Cu(II) complexes tend to be more stable than their Fe(III) counterparts. Higher concentrations of AG would favor the formation of more stable complexes because the 1:3 Fe(III) complexes with bidentate AG_D_ species are much more stable than the most sTable 1:2 Cu(II) complex. In other words, from our calculations, it could be interpreted that low concentrations of AG in environments where similar biochemical concentrations of Fe(III) and Cu(II) exist would favor the formation of complexes with Cu(II). However, Fe(III) complex formation would be more probable at much greater AG concentrations.

Our kinetic calculations show that AG has no secondary antioxidant activity relative to the Fe(III)/Fe(II) (and the Cu(II)/Cu(I)) [23] reduction with the superoxide radical anion. However, a very different situation is observed relative to the reduction of both Fe(III) and Cu(II) with ascorbate because AG is able to inhibit the formation of ^•^OH radicals due to the Fenton reaction. A low-spin octahedral Fe(III) complex is able to produce a *k* value for the first step of the Haber–Weiss cycle that is 1.7 × 10^9^ times less than the *k* of the reference reaction with ascorbate. The most stable Fe(III) complex can reduce this *k* by 7.9 × 10^5^ times. Relative to the Cu(II)/Cu(I) reduction with ascorbate, the largest *k* reductions are in the order of 10^7^ times. Experimental work confirms the *k* reduction relative to reduction with ascorbate upon the complexation of AG with Cu(II) and Fe(III) ions [9,21].

## Figures and Tables

**Table 1:** Standard Gibbs free energy change ($∆G_{f}^{{}^{\circ}}$ , in kcal/mol) and formation constant ($K_{f}$, ${\mathrm{logK}}_{f}$) for the most relevant complexes of Fe(III) with AG (as per Equation (7)) in aqueous solution at 298.15 K, taking into account the $∆G^{{}^{\circ}}$ to form AG from AGH^+^ at physiological pH ^a^.

Complex ${[\mathrm{Fe}\left(\mathrm{AG}\right){\times (H}_{2}{O)}_{n}]}^{3+}$	$∆G_{f}^{{}^{\circ}}{}_{{\mathrm{Fe}}^{3+}{-\mathrm{AG}}^{}}$	$K_{f_{{\mathrm{Fe}}^{3+}{-\mathrm{AG}}^{}}}$	${\mathrm{logK}}_{f_{{\mathrm{Fe}}^{3+}{-\mathrm{AG}}^{}}}$
{14} [Fe(AG_D_)_3_]^3+^ hs (N_2_, N_4_)	−36.8	9.03 × 10^26^	26.96
{15} [Fe(AG_D_)_3_]^3+^ ls (N_2_, N_4_)	−6.7	8.22 × 10^4^	4.91
{16} [Fe(AG_D_)_3_]^3+^ hs (same orientation, N_2_, N_4_)	−37.9	5.65 × 10^27^	27.75
{17} [Fe(AG_D_)_3_(H_2_O)]^3+^ hs (N_2_, N_4_, N_2′_, N_4′_, N_2′_)	−24.1	4.96 × 10^17^	17.70
{18} [Fe(AG_D_)_3_(H_2_O)_2_]^3+^ hs (N_2_, N_4_, N_2′_, N_2′_)	−19.1	1.03 × 10^14^	14.01
			
{32} [Fe(AG_D_)_2_(H_2_O)_2_]^3+^ hs (trans, N_2_, N_4_)	−20.1	5.35 × 10^14^	14.73
{34} [Fe(AG_D_)_2_(H_2_O)_2_]^3+^ hs (mirror image, N_2_, N_4_)	−19.0	7.91 × 10^13^	13.90
{38} [Fe(AG_D_)_2_(H_2_O)_2_]^3+^ hs (cis, N_2_, N_4_)	−22.9	5.86 × 10^16^	16.77
			
{43} [Fe(AG_D_)(H_2_O)_4_]^3+^ hs (N_2_, N_4_)	−10.3	3.48 × 10^7^	7.54
			
{49} [Fe(AG_A_)_2_(H_2_O)_2_]^3+^ hs (4-coord., N_2_)	−19.6	2.20 × 10^14^	14.34
{51} [Fe(AG_B_)_2_(H_2_O)_2_]^3+^ hs (4-coord., N_2_)	−21.6	6.35 × 10^15^	15.80
{54} [Fe(AG_C_)_2_(H_2_O)_2_]^3+^ hs (4-coord., N_2_)	−20.5	1.09 × 10^15^	15.04
{57} [Fe(AG_D_)(H_2_O)_3_]^3+^ hs (5-coord., sq. pyr., N_2_, N_4_)	−15.3	1.63 × 10^11^	11.21
{59} [Fe(AG_D_)_2_]^3+^ hs (4-coord., N_2_, N_4_)	−20.5	1.02 × 10^15^	15.01
{60} [Fe(AG_D_)_2_(H_2_O)]^3+^ hs (5-coord., N_2_, N_4_)	−23.5	1.78 × 10^17^	17.25

^a^ The octahedral complexes are grouped according to the number of AG ligands present (3, 2 or 1), followed by non-octahedral complexes; coordinating atoms in the organic ligand are shown in parentheses for each complex, and high- (hs) and low-spin (ls) complexes are identified.

**Table 2:** Rate constants (in M^−1^ s^−1^) for the reduction of Fe(III) and Cu(II) complexes (with and without complexation with AG) with $O_{2}^{•-}$ and ascorbate (${\mathrm{ASC}}^{-}$,) in aqueous solution at 298.15 K and the rate constant ratios (using the reduction of ${\left[\mathrm{Fe}{\left(H_{2}O\right)}_{6}\right]}^{3+}$ or ${\left[\mathrm{Cu}{\left(H_{2}O\right)}_{4}\right]}^{2+}$ as a reference) ^a,b,c^.

	${\mathrm{Ox}}^{-}{=O}_{2}^{•-}$		${\mathrm{Ox}}^{-}={\;\mathrm{ASC}}^{-}$	
Reaction	*k_app_*	Ratio	*k_app_*	Ratio
${\left[\mathrm{Fe}{\left(H_{2}O\right)}_{6}\right]}^{3+}+Ox^{-}\to {\left[\mathrm{Fe}{\left(H_{2}O\right)}_{6}\right]}^{2+}+Ox$	7.28 × 10^9^		7.43 × 10^9^	
{15} $+\;Ox^{-}$ → {61} + Ox (ls)	7.76 × 10^9^	0.94	4.48	1.66 × 10^9^
{16} $+\;Ox^{-}$ → {62} + Ox	8.09 × 10^9^	0.90	9.47 × 10^3^	7.85 × 10^5^
{14} $+\;Ox^{-}$ → {63} + Ox	8.29 × 10^9^	0.88	1.96 × 10^5^	3.79 × 10^4^
{17} $+\;Ox^{-}$ → {64} + Ox	8.30 × 10^9^	0.88	1.53 × 10^8^	48.6
{38} $+\;Ox^{-}$ → {65} + Ox	8.08 × 10^9^	0.90	6.57 × 10^8^	11.3
${\left[\mathrm{Cu}{\left(H_{2}O\right)}_{4}\right]}^{2+}+Ox^{-}\to {\left[\mathrm{Cu}{\left(H_{2}O\right)}_{2}\right]}^{+}\cdot 2H_{2}O+Ox$	7.71 × 10^9^		2.10 × 10^9^	
$\left\{24\right\}+Ox^{-}\to \left\{42\right\}+Ox$	2.80 × 10^9^	2.75	60.9	3.45 × 10^7^
$\left\{23\right\}+Ox^{-}\to \left\{41\right\}+Ox$	3.49 × 10^9^	2.21	66.6	3.15 × 10^7^
$\left\{25\right\}+Ox^{-}\to \left\{40\right\}+Ox$	7.02 × 10^9^	1.10	3.56 × 10^4^	5.90 × 10^4^
$\left\{29\right\}+Ox^{-}\to \left\{39\right\}+Ox$	7.40 × 10^9^	1.04	8.01 × 10^4^	2.62 × 10^4^
$\left\{11\right\}+Ox^{-}\to \left\{38\right\}+Ox$	7.47 × 10^9^	1.03	4.83 × 10^5^	4.35 × 10^3^

^a^ For additional kinetic and thermodynamic information on these and other reactions studied, refer to Tables S6–S8; *k* is reported instead of *k_app_* when diffusion corrections were not necessary. ^b^ The iron complexes are high spin, unless otherwise indicated (ls = low spin); ^c^ The results for the Cu(II) reduction with $O_{2}^{•-}$ and the labeling system for these complexes have been taken from [23].

## Supplementary Materials

The supplementary materials are available online at: https://www.mdpi.com/2076-3921/9/8/756/s1.

## References

[1] Thorpe S.R., Baynes J.W. (2003). Maillard reaction products in tissue proteins: New products and new perspectives. Amino Acids.

[2] Ulrich P., Cerami A. (2001). Protein glycation, diabetes, and aging. Recent Prog. Horm. Res..

[3] Münch G., Mayer S., Michaelis J., Hipkiss A.R., Riederer P., Müller R., Neumann A., Schinzel R., Cunningham A.M. (1997). Influenced of advanced glycation end-products and AGE-inhibitors on nucleation-dependent polymerization of beta-amyloid peptide. Biochim. Biophys. Acta.

[4] Stitt A.W. (2005). The maillard reaction in eye diseases. Ann. N. Y. Acad. Sci..

[5] Colzani M., De Maddis D., Casali G., Carini M., Vistoli G., Aldini G. (2016). Reactivity, selectivity, and reaction mechanisms of aminoguanidine, hydralazine, pyridoxamine, and carnosine as sequestering agents of reactive carbonyl species: A comparative study. Chem. Med. Chem..

[6] Haber F., Weiss J. (1932). On the catalyst of hydroperoxide. Naturwissenschaften.

[7] Zhao M.J., Jung L. (1995). Kinetics of the competitive degradation of deoxyribose and other molecules by hydroxyl radicals produced by the fenton reaction in the presence of ascorbic acid. Free Radic. Res..

[8] Burkitt M.J., Gilbert B.C. (1990). Model studies of the iron-catalysed haber-weiss cycle and the ascorbate-driven fenton reaction. Free Radic. Res. Commun..

[9] Price D.L., Rhett P.M., Thorpe S.R., Baynes J.W. (2001). Chelating activity of advanced glycation end-products inhibitors. J. Biol. Chem..

[10] Rahbar S., Natarajan R., Yerneni K.K., Scott S., Gonzales N., Nadler J.L. (2000). Evidence that pioglitazone, metformin and pentoxifylline are inhibitors of glycation. Clin. Chim. Acta.

[11] Ortega-Castro J., Adrover M., Frau J., Donoso J., Muñoz F. (2008). Chelating power of LR-74, a new AGE-inhibitor. Chem. Phys. Lett..

[12] Casasnovas R., Ortega-Castro J., Donoso J., Frau F., Muñoz F. (2013). Theoretical calculations of stability constants and pKa values of metal complexes in solution: Application to pyridoxamine-copper(II) complexes and their biological implications in AGE inhibition. Phys. Chem. Chem. Phys..

[13] Abdel-Rahman E., Bolton W.K. (2002). Pimagedine: A novel therapy for diabetic nephropathy. Expert Opin. Investig. Drugs.

[14] Li Y.M., Steffes M., Donnelly T., Liu C., Fuh H., Basgen J., Bucala R., Vlassara H. (1996). Prevention of cardiovascular and renal pathology of aging by the advanced glycation inhibitor aminoguanidine. Proc. Natl. Acad. Sci. USA.

[15] Ortega-Castro J., Frau J., Casasnovas R., Fernández D., Donoso J., Muñoz F. (2012). High- and low-spin Fe(III) complexes of various AGE inhibitors. J. Phys. Chem. A.

[16] Ortega-Castro J., Adrover M., Frau J., Donoso J., Muñoz F. (2009). Cu^2+^ complexes of some AGEs inhibitors. Chem. Phys. Lett..

[17] Ortega-Castro J., Adrover M., Frau J., Salvà A., Donoso J., Muñoz F. (2010). DFT studies on schiff base formation of vitamin B6 analogues. Reaction between a pyridoxamine-analogue and carbonyl compounds. J. Phys. Chem. A.

[18] Adrover M., Vilanova B., Muñoz F., Donoso J. (2007). Pyridxamine, a scavenger agent of carbohydrates. Int. J. Chem. Kinet..

[19] Adrover M., Vilanova B., Muñoz F., Donoso J. (2005). Inhibition of glycosylation processes: The reaction between pyridoxamine and glucose. Chem. Biodivers..

[20] Solís-Calero C., Ortega-Castro J., Frau J., Muñoz F. (2015). Scavenger mechanism of methylglyoxal by metformin. A DFT study. Theor. Chem. Acc..

[21] Rahbar S., Figarola J.L. (2003). Novel inhibitors of advanced glycation endproducts. Arch. Biochem. Biophys..

[22] Ramis R., Casasnovas R., Mariño L., Frau J., Adrover M., Vilanova B., Mora-Diez N., Ortega-Castro J. (2019). A density functional theory study of the free-radical scavenging activity of aminoguanidine. Comparison with its reactive carbonyl compound and metal scavenging activities. Int. J. Quantum Chem..

[23] García-Díez G., Ramis R., Mora-Diez N. (2020). Theoretical study of the copper complexes with aminoguanidine: Investigating secondary antioxidant activity. ACS Omega.

[24] Monreal-Corona R., Ippolito A.A., Biddlecombe J.R., Mora-Diez N. (2020). Theoretical study of the iron complexes with lipoic and dihydrolipoic acids: Exploring secondary antioxidant activity. Antioxidants.

[25] Frisch M.J., Trucks G.W., Schlegel H.B., Scuseria G.E., Robb M.A., Cheeseman J.R., Scalmani G., Barone V., Mennucci B., Petersson G.A. (2010). Gaussian09, Revision B.01.

[26] Marenich A.V., Cramer C.J., Truhlar D.G. (2009). Universal solvation model based on solute electron density and on a continuum model of the solvent defined by the bulk dielectric constant and atomic surface tensions. J. Phys. Chem. B.

[27] Zhao Y., Schultz N.E., Truhlar D.G. (2005). Exchange-correlation functional with broad accuracy for metallic and non-metallic compounds, kinetics, and noncovalent interactions. J. Chem. Phys..

[28] Zhao Y., Truhlar D.G. (2008). The M06 suite of density functional for main group thermochemistry, thermochemical kinetics, noncovalent interactions, excited states, and transition metals: Two new functionals and systematic testing of four M06-class functionals and 12 other functionals. Theor. Chem. Acc..

[29] Marcus R.A. (1993). Electrons transfer reactions in chemistry. Theory and experiment. Rev. Mod. Phys..

[30] Marcus R.A. (1997). Transfer reactions in chemistry. Theory and experiment. Pure Appl. Chem..

[31] Collins F.C., Kimball G.E. (1949). Diffusion-controlled reaction rates. J. Colloid. Sci..

[32] Smoluchowskim M.Z. (1917). Versuch einer mathematischen theorie der koagulationskinetik kolloider Lösungen. J. Phys. Chem..

[33] Einstein A. (1905). Über die von der molekularkinetischen theorie der wärme geforderte bewegung von in ruhenden flüssigkeiten suspendierten teilchen. Ann. Phys..

[34] Stokes G.G. (1903). Mathematical and Physical Papers.

[35] Castañeda-Arriaga R., Alvarez-Idaboy J.R., Mora-Diez N. (2016). Theoretical study of copper complexes with lipoic and dihydrolipoic acids. RSC Adv..

[36] Castañeda-Arriaga R., Mora-Diez N., Alvarez-Idaboy J.R. (2015). Modelling the chemical repair of protein carbon-centered radicals formed via oxidative damage with dihydrolipoic acid. RCS Adv..

[37] Castañeda-Arriaga R., Domínguez-Casto A., Lee J., Alvarez-Idaboy J.R., Mora-Diez N. (2016). Chemical repair of protein-centred radicals: Long-distance dynamic factors. Can. J. Chem..

[38] Ramis R., Casasnovas R., Ortega-Castro J., Frau J., Alvarez-Idaboy J.R., Mora-Diez N. (2019). Modelling the repair of carbon-centered protein radicals by the antioxidants glutathione and Trolox. New J. Chem..

[39] Galano A., Alvarez-Idaboy J.R. (2013). A computational methodology for accurate predictions of rate constants in solution: Application to the assessment of primary antioxidant activity. J. Comput. Chem..

[40] Romeo I., Parise A., Galano A., Russo N., Alvarez-Idaboy J.R., Marino T. (2020). The antioxidant capability of higenamine: Insights from theory. Antioxidants.

[41] Goel S., Masunov A.E., Allen G., Nabrzyski J., Seidel E., van Albada G.D., Dongarra J., Sloot P.M.A. (2009). Pairwise spin-contamination correction method and dft study of mnh and h2 dissociation curves. Computational Science—ICCS 2009.

[42] Koskinen M., Mutikainen I., Tilus P., Pelttari E., Korvela M., Elo H. (1997). Structure of aminoguanidine hemioxalate. Implications for the synthesis of amidinohydrazones. Mon. Chem..

[43] Greenwood N.N., Earnshaw A. (1997). Chemistry of the Elements.

[44] Edler E., Stein M. (2014). Spin-state-dependent properties of an Iron(III) hydrogenase mimic. Eur. J. Inorg. Chem..

[45] Boldyrev V.V., Tukhtaev R.K., Gavrilov A.I., Larionov S.V., Savel’eva Z.A., Lavrenova L.G. (1998). Combustion of nickel and copper nitrate complexes of hydrazine derivatives as a method for manufacturing fine-grained and porous metals. Russ. J. Inorg. Chem..

[46] Buettner G.G. (1986). Ascorbate autoxidation in the presence of iron and copper chelates. Free Rad. Res. Commun..

[47] Francisco-Marquez M., Aguilar-Fernánde M., Galano A. (2016). Anthranilic acid as a secondary antioxidant: Implications to the inhibition of OH production and the associated oxidative stress. Comp. Theor. Chem..

[48] Martínez A., Vargas R., Galano A. (2018). Citric acid: A promising copper scavenger. Comp. Theor. Chem..

[49] Castañeda-Arriaga R., Pérez-González A., Reina M., Alvarez-Idaboy J.R., Galano A. (2018). Comprehensive investigation of the antioxidant and pro-oxidant effects of phenolic compounds: A double-edged sword in the context of oxidative stress?. J. Phys. Chem. B.

[50] Mravljak J., Jakopin Z. (2019). Iron-binding and anti-fenton properties of novel amino acid-derived cyclic imide dioximes. Antioxidants.

[51] Kubicova L., Hadacek F., Bachmann G., Weckwerth W., Chobot V. (2019). Coordination complex formation and redox properties of kynurenic and xanthurenic acid can affect brain tissue homeodynamics. Antioxidants.

[52] Brovč E.V., Pajk S., Šink R., Mravljak J. (2020). Protein formulations containing polysorbates: Are metal chelators needed at all?. Antioxidants.

[53] Wang L., Santos E., Schenk D., Rabago-Smith M. (2014). Kinetics and mechanistic studies on the reaction between cytochrome c and tea catechins. Antioxidants.

[54] Guerreiro J.F., Gomes M.A.G.B., Pagliari F., Jansen J., Marafioti M.G., Nistico C., Hanley R., Costa R.O., Ferreira S.S., Mendes F. (2020). Iron and copper complexes with antioxidant activity as inhibitors of the metastatic potential of glioma cells. RSC Adv..

[55] Bielski B.H.J., Cabelli D.E., Arudi R.L., Ross A.B. (1985). Reactivity of HO_2_/O^−^_2_ radicals in aqueous solution. J. Phys. Chem. Ref. Data.

