# Supplementary material for: Theoretical Study of the Iron Complexes with Aminoguanidine: Investigating Secondary Antioxidant Activity
Source: Antioxidants (Basel). 2020 Aug 15;9(8):756. doi: 10.3390/antiox9080756 (PMC7463863; doi:10.3390/antiox9080756)

Theoretical Study of the Iron Complexes with Aminoguanidine: Investigating Secondary Antioxidant Activity

Guillermo García-Díez, Nelaine Mora-Diez

Thompson Rivers University, Department of Chemistry, Kamloops, B.C., V2C 0C8 Canada.

Supplementary Materials

(73 pages)

Contents:

Table S1. Absolute enthalpies and Gibbs free energies of the different species considered in this study at the M05(SMD)/6-311+G(d,p) level of theory in water at 298.15 K.

Table S2. $\langle \hat{S}^2 \rangle$ values for the calculated open-shell iron complexes before and after annihilation of the first spin contaminant.

Table S3. Standard Gibbs free energy change (ΔG_f°, in kcal/mol) and formation constant (K_f, $logK_f$) for the chelation of Fe(III) with AGH^+ (as per Equation 5) in aqueous solution at 298.15 K.

Table S4. Standard Gibbs free energy change (ΔG_f°) and formation constant (K_f, $logK_f$) for the calculated Fe(III) complexes with AG (as per Equation 6) in aqueous solution at 298.15 K.

Table S5. Standard Gibbs free energy change (ΔG_f°, in kcal/mol) and formation constant (K_f, $logK_f$) for the calculated Fe(III) complexes with AG (as per Equation 7) in aqueous solution at 298.15 K, taking into account the ΔG° to form AG from AGH^+ at physiological pH.

Table S6. Standard Gibbs free energy of reaction (ΔG°, kcal/mol) and activation (ΔG≠, kcal/mol), various rate constants (k, k_D and k_{app}, M^{-1} s^{-1}) and the rate constant ratio (using k_{app} for the reduction of $[Fe(H_2O)_6]^{3+}$as reference) for the initial reaction of the Haber-Weiss cycle (with and without iron complexation with AG) with $O_2^{\bullet-}$ in aqueous solution at 298.15 K.

Table S7. Standard Gibbs free energy of reaction (ΔG°, kcal/mol) and activation (ΔG≠, kcal/mol), various rate constants (k, k_D and k_{app}, M^{-1} s^{-1}) and the rate constant ratio (using k_{app} for the reduction of $[Fe(H_2O)_6]^{3+}$ as reference) for the initial reaction of the Haber-Weiss cycle (with and without iron complexation with AG) with ascorbate (ASC^-) in aqueous solution at 298.15 K.

Table S8. Standard Gibbs free energy of reaction (ΔG°, kcal/mol) and activation (ΔG≠, kcal/mol), various rate constants (k, k_D and k_{app}, M^{-1} s^{-1}) and the rate constant ratio (using k_{app} for the reduction of $[Cu(H_2O)_4]^{2+}$as reference) for the initial reaction of the Haber-Weiss cycle (with and without copper complexation with AG or AGH^+) with ascorbate (ASC^-) in aqueous solution at 298.15 K.

Figure S1. Optimized geometries of complexes of Fe(III) with AGH^+ in aqueous solution (bond distances in Å); high- (hs) and low-spin (ls) complexes are identified.

Figure S2. Optimized geometries of the 1:3, 1:2 and 1:1 Fe(III) complexes with AG in aqueous solution that are not listed in Table 1 (bond distances in Å); high- (hs) and low-spin (ls) complexes are identified.

Figure S3. Optimized geometries of Fe(II) complexes with AG in aqueous solution that appear in Tables S6 and S7, but are not shown in Figure 3 (indicating the Fe(III) complex used as starting point in each case; bond distances in Å); these complexes are high spin (hs).

Figure S4. Plots of the apparent rate constant (k_{app}) for the SET reactions of Fe(III) complexes with $O_2^{\bullet-}$ (left axis: k_{app}; marker: circles) and ASC^- (right axis: log k_{app}; marker: triangles) *versus* the ΔG_f° of the Fe(III) complexes.

Figure S5. Plots of the apparent rate constant (k_{app}) for the SET reactions of Cu(II) complexes with $O_2^{\bullet-}$ (left axis: k_{app}; marker: circles) and ASC^- (right axis: log k_{app}; marker: triangles) *versus* the ΔG_f° of the Cu(II) complexes studied.

Appendix 1. Additional explanation on corrections to calculated Gibbs free energies in solution and the approach followed in this study.

M05(SMD)/6-311+G(d,p) Cartesian coordinates of the optimized geometries in water of the species calculated in this study.

Table S1. Absolute enthalpies and Gibbs free energies of the different species considered in this study at the M05(SMD)/6-311+G(d,p) level of theory in water at 298.15 K.

	Species	H° (au)	G° (au)
{1}	$[Fe(AGH)_3]^{6+}$ hs	-2046.147951	-2046.214534
{2}	$[Fe(AGH)_3]^{6+}$ ls	-2046.089274	-2046.151155
{3}	$[Fe(AGH)_2(H_2O)_2]^{5+}$ hs	-1938.031738	-1938.094046
{4}	$[Fe(AGH)_2(H_2O)_2]^{5+}$ ls	-1937.951636	-1938.011213
{5}	$[Fe(AGH)(H_2O)_4]^{4+}$ hs	-1829.898763	-1829.956527
{6}	$[Fe(AGH)(H_2O)_4]^{4+}$ ls	-1829.806063	-1829.859624
{7}	$[Fe(AGH)(H_2O)_5]^{4+}$ hs	-1906.329601	-1906.395120
{8}	$[Fe(AGH)_2(H_2O)_4]^{5+}$ hs	-2090.894712	-2090.970981
{9}	$[Fe(AGH)_2]^{5+}$ hs	-1785.187679	-1785.240264
{10}	$[Fe(AGH)_2]^{5+}$ ls	-1785.059791	-1785.111267
{11}	$[Fe(AG_B)_3]^{3+}$ hs	-2044.864999	-2044.930359
{12}	$[Fe(AG_B)_3]^{3+}$ hs (same orientation)	-2044.862444	-2044.927825
{13}	$[Fe(AG_C)_3]^{3+}$ hs	-2044.868200	-2044.933191
{14}	$[Fe(AG_D)_3]^{3+}$ hs	-2044.963854	-2045.036195
{15}	$[Fe(AG_D)_3]^{3+}$ ls	-2044.923115	-2044.988277
{16}	$[Fe(AG_D)_3]^{3+}$ hs (same orientation)	-2044.965806	-2045.037927
{17}	$[Fe(AG_D)_3(H_2O)]^{3+}$ hs	-2121.358608	-2121.435251
{18}	$[Fe(AG_D)_3(H_2O)_2]^{3+}$ hs	-2197.765426	-2197.844264
{19}	$[Fe(AG_A)_2(H_2O)_2]^{3+}$ ls	-1937.100324	-1937.164154
{20}	$[Fe(AG_A)_2(H_2O)_2]^{3+}$ ls (mirror image)	-1937.099724	-1937.163492
{21}	$[Fe(AG_A)_2(H_2O)_4]^{3+}$ hs	-2090.037842	-2090.116078
{22}	$[Fe(AG_B)_2(H_2O)_2]^{3+}$ hs	-1937.160171	-1937.222282
{23}	$[Fe(AG_B)_2(H_2O)_2]^{3+}$ ls	-1937.099498	-1937.157699
{24}	$[Fe(AG_B)_2(H_2O)_2]^{3+}$ hs (mirror image)	-1937.159165	-1937.221740
{25}	$[Fe(AG_B)_2(H_2O)_2]^{3+}$ ls (mirror image)	-1937.100327	-1937.158558
{26}	$[Fe(AG_B)_2(H_2O)_4]^{3+}$ hs	-2090.036344	-2090.114646
{27}	$[Fe(AG_C)_2(H_2O)_2]^{3+}$ hs	-1937.161822	-1937.222789
{28}	$[Fe(AG_C)_2(H_2O)_2]^{3+}$ ls	-1937.101219	-1937.159665
{29}	$[Fe(AG_C)_2(H_2O)_2]^{3+}$ hs (mirror image)	-1937.160976	-1937.222068
{30}	$[Fe(AG_C)_2(H_2O)_2]^{3+}$ ls (mirror image)	-1937.101379	-1937.159957
{31}	$[Fe(AG_C)_2(H_2O)_4]^{3+}$ hs	-2090.036459	-2090.115474
{32}	$[Fe(AG_D)_2(H_2O)_2]^{3+}$ hs (trans)	-1937.228087	-1937.294490
{33}	$[Fe(AG_D)_2(H_2O)_2]^{3+}$ ls	-1937.168260	-1937.229903
{34}	$[Fe(AG_D)_2(H_2O)_2]^{3+}$ hs (mirror image)	-1937.227286	-1937.292685
{35}	$[Fe(AG_D)_2(H_2O)_2]^{3+}$ ls (mirror image)	-1937.165663	-1937.226595
{36}	$[Fe(AG_C)(AG_D)(H_2O)_2]^{3+}$ hs	-1937.195093	-1937.259656
{37}	$[Fe(AG_C)(AG_D)(H_2O)_2]^{3+}$ ls	-1937.136065	-1937.196750

{38}	$[Fe(AG_D)_2(H_2O)_2]^{3+}$ hs (cis)	-1937.232179	-1937.298924
{39}	$[Fe(AG_D)_2(H_2O)_3]^{3+}$ hs	-2013.630427	-2013.702795
{40}	$[Fe(AG_D)_2(H_2O)_4]^{3+}$ hs	-2090.028602	-2090.106185
{41}	$[Fe(AG_B)(H_2O)_4]^{3+}$ hs	-1829.466860	-1829.525106
{42}	$[Fe(AG_C)(H_2O)_4]^{3+}$ hs	-1829.526437	-1829.526437
{43}	$[Fe(AG_D)(H_2O)_4]^{3+}$ hs	-1829.500673	-1829.560921
{44}	$[Fe(AG_A)(H_2O)_5]^{3+}$ hs	-1905.906769	-1905.972698
{45}	$[Fe(AG_B)(H_2O)_5]^{3+}$ hs	-1905.904931	-1905.973162
{46}	$[Fe(AG_C)(H_2O)_5]^{3+}$ hs	-1905.907643	-1905.972435
{47}	$[Fe(AG_D)(H_2O)_5]^{3+}$ hs	-1905.903343	-1905.968937
{48}	$[Fe(AG_A)_2(H_2O)_2]^{3+}$ hs (5-coord.)	-1937.193129	-1937.261388
{49}	$[Fe(AG_A)_2(H_2O)_2]^{3+}$ hs (4-coord.)	-1937.226807	-1937.293650
{50}	$[Fe(AG_A)(H_2O)_4]^{3+}$ hs (5-coord.)	-1829.495579	-1829.557475
{51}	$[Fe(AG_B)_2(H_2O)_2]^{3+}$ hs (4-coord.)	-1937.228225	-1937.296825
{52}	$[Fe(AG_B)(H_2O)_4]^{3+}$ hs (5-coord.)	-1829.498656	-1829.560988
{53}	$[Fe(AG_C)_3]^{3+}$ hs (5-coord.)	-2044.863684	-2044.928511
{54}	$[Fe(AG_C)_2(H_2O)_2]^{3+}$ hs (4-coord.)	-1937.226474	-1937.295165
{55}	$[Fe(AG_C)(H_2O)_4]^{3+}$ hs (5-coord.)	-1829.500433	-1829.562993
{56}	$[Fe(AG_D)(H_2O)_2]^{3+}$ hs (4-coord.)	-1676.671835	-1676.724558
{57}	$[Fe(AG_D)(H_2O)_3]^{3+}$ hs (5-coord., sq. pyr.)	-1753.093154	-1753.150513
{58}	$[Fe(AG_D)(H_2O)_3]^{3+}$ hs (5-coord., tr. bipy.)	-1753.085994	-1753.141910
{59}	$[Fe(AG_D)_2]^{3+}$ hs (4-coord.)	-1784.402616	-1784.458888
{60}	$[Fe(AG_D)_2(H_2O)]^{3+}$ hs (5-coord.)	-1860.822714	-1860.882948
{61}	$[Fe(AG_D)_3]^{2+}$ ls	-2045.070931	-2045.136202
{62}	$[Fe(AG_D)_3]^{2+}$ hs (same orientation)	-2045.122251	-2045.193693
{63}	$[Fe(AG_D)_3]^{2+}$ hs	-2045.123289	-2045.196321
{64}	$[Fe(AG_D)_3(H_2O)]^{2+}$ hs	-2121.531469	-2121.612006
{65}	$[Fe(AG_D)_2(H_2O)_2]^{2+}$ hs (cis)	-1937.402149	-1937.470780
{66}	$[Fe(AG_D)_2(H_2O)_2]^{2+}$ hs (mirror image)	-1937.398816	-1937.467530
{67}	$[Fe(AG_D)_2(H_2O)_2]^{2+}$ hs (trans)	-1937.398993	-1937.468307
{68}	$[Fe(AG_A)_2(H_2O)_2]^{2+}$ hs (4-coord.)	-1937.399196	-1937.472016
{69}	$[Fe(AG_B)_2(H_2O)_2]^{2+}$ hs (4-coord.)	-1937.398946	-1937.473791
{70}	$[Fe(AG_C)_2(H_2O)_2]^{2+}$ hs (4-coord.)	-1937.398728	-1937.472349
{71}	$[Fe(AG_D)(H_2O)_3]^{2+}$ hs (5-coord., sq. pyr.)	-1753.274093	-1753.336408
{72}	$[Fe(AG_D)_2(H_2O)]^{2+}$ hs (5-coord.)	-1860.997892	-1861.061384
{73}	$[Fe(AG_D)(H_2O)_5]^{2+}$ hs	-1906.081197	-1906.151981
{74}	$[Fe(AG_B)(H_2O)_5]^{2+}$ hs	-1906.083307	-1906.161481
{75}	$[Fe(AG_D)(H_2O)_4]^{2+}$ hs	-1829.679935	-1829.748090
{76}	$[Fe(AG_A)(H_2O)_4]^{2+}$ hs (5-coord.)	-1829.678804	-1829.746985
{77}	$[Fe(AG_D)_2]^{2+}$ hs (4-coord.)	-1784.583356	-1784.639416
{78}	$[Fe(H_2O)_6]^{3+}$ hs	-1721.770188	-1721.825920

{79}	$[Fe(H_2O)_6]^{3+}$ ls	-1721.662916	-1721.714536
{80}	$[Fe(H_2O)_6]^{2+}$ hs	-1721.959274	-1722.023834
{81}	AGH^+	-260.984009	-261.021567
{82}	$(AGH^+)_2$	-521.968983	-522.026679
{83}	$(AGH^+)_3$	-782.953159	-783.031044
{84}	AG_A	-260.521678	-260.556867
{85}	AG_B	-260.521390	-260.556485
{86}	AG_C	-260.521218	-260.556191
{87}	AG_D	-260.517382	-260.553434
{88}	$(AG_A)_2$	-521.049437	-521.103032
{89}	$(AG_A)_3$	-781.571327	-781.645452
{90}	ASC^-	-684.045489	-684.096816

Table S2. $\langle \hat{S}^2 \rangle$ values for the calculated open-shell iron complexes before and after annihilation of the first spin contaminant.

	Species	$\langle \hat{S}^2 \rangle$ before annihilation	$\langle \hat{S}^2 \rangle$ after annihilation
{1}	$[Fe(AGH)_3]^{6+}$ hs	8.7583	8.7500
{2}	$[Fe(AGH)_3]^{6+}$ ls	0.8801	0.7599
{3}	$[Fe(AGH)_2(H_2O)_2]^{5+}$ hs	8.7579	8.7500
{4}	$[Fe(AGH)_2(H_2O)_2]^{5+}$ ls	0.8798	0.7510
{5}	$[Fe(AGH)(H_2O)_4]^{4+}$ hs	8.7557	8.7500
{6}	$[Fe(AGH)(H_2O)_4]^{4+}$ ls	1.1173	0.8234
{7}	$[Fe(AGH)(H_2O)_5]^{4+}$ hs	8.7559	8.7500
{8}	$[Fe(AGH)_2(H_2O)_4]^{5+}$ hs	8.7567	8.7500
{9}	$[Fe(AGH)_2]^{5+}$ hs	8.7582	8.7500
{10}	$[Fe(AGH)_2]^{5+}$ ls	0.7814	0.7505
{11}	$[Fe(AG_B)_3]^{3+}$ hs	8.7597	8.7500
{12}	$[Fe(AG_B)_3]^{3+}$ hs (same orientation)	8.7600	8.7500
{13}	$[Fe(AG_C)_3]^{3+}$ hs	8.7603	8.7500
{14}	$[Fe(AG_D)_3]^{3+}$ hs	8.7593	8.7500
{15}	$[Fe(AG_D)_3]^{3+}$ ls	0.7625	0.7501
{16}	$[Fe(AG_D)_3]^{3+}$ hs (same orientation)	8.7594	8.7500
{17}	$[Fe(AG_D)_3(H_2O)]^{3+}$ hs	8.7586	8.7500
{18}	$[Fe(AG_D)_3(H_2O)_2]^{3+}$ hs	8.7577	8.7500
{19}	$[Fe(AG_A)_2(H_2O)_2]^{3+}$ ls	0.9654	0.7524
{20}	$[Fe(AG_A)_2(H_2O)_2]^{3+}$ ls (mirror image)	1.0111	0.7527
{21}	$[Fe(AG_A)_2(H_2O)_4]^{3+}$ hs	8.7575	8.7500
{22}	$[Fe(AG_B)_2(H_2O)_2]^{3+}$ hs	8.7578	8.7500

{23}	$[Fe(AG_B)_2(H_2O)_2]^{3+}$ ls	0.9149	0.7516
{24}	$[Fe(AG_B)_2(H_2O)_2]^{3+}$ hs (mirror image)	8.7583	8.7500
{25}	$[Fe(AG_B)_2(H_2O)_2]^{3+}$ ls (mirror image)	0.9306	0.7517
{26}	$[Fe(AG_B)_2(H_2O)_4]^{3+}$ hs	8.7580	8.7500
{27}	$[Fe(AG_C)_2(H_2O)_2]^{3+}$ hs	8.7580	8.7500
{28}	$[Fe(AG_C)_2(H_2O)_2]^{3+}$ ls	0.9440	0.7519
{29}	$[Fe(AG_C)_2(H_2O)_2]^{3+}$ hs (mirror image)	8.7582	8.7500
{30}	$[Fe(AG_C)_2(H_2O)_2]^{3+}$ ls (mirror image)	0.9439	0.7517
{31}	$[Fe(AG_C)_2(H_2O)_4]^{3+}$ hs	8.7574	8.7500
{32}	$[Fe(AG_D)_2(H_2O)_2]^{3+}$ hs (trans)	8.7583	8.7500
{33}	$[Fe(AG_D)_2(H_2O)_2]^{3+}$ ls	0.8154	0.7505
{34}	$[Fe(AG_D)_2(H_2O)_2]^{3+}$ hs (mirror image)	8.7580	8.7500
{35}	$[Fe(AG_D)_2(H_2O)_2]^{3+}$ ls (mirror image)	0.8304	0.7506
{36}	$[Fe(AG_C)(AG_D)(H_2O)_2]^{3+}$ hs	8.7583	8.7500
{37}	$[Fe(AG_C)(AG_D)(H_2O)_2]^{3+}$ ls	0.8681	0.7510
{38}	$[Fe(AG_D)_2(H_2O)_2]^{3+}$ hs (cis)	8.7579	8.7500
{39}	$[Fe(AG_D)_2(H_2O)_3]^{3+}$ hs	8.7576	8.7500
{40}	$[Fe(AG_D)_2(H_2O)_4]^{3+}$ hs	8.7571	8.7500
{41}	$[Fe(AG_B)(H_2O)_4]^{3+}$ hs	8.7560	8.7500
{42}	$[Fe(AG_C)(H_2O)_4]^{3+}$ hs	8.7561	8.7500
{43}	$[Fe(AG_D)(H_2O)_4]^{3+}$ hs	8.7568	8.7500
{44}	$[Fe(AG_A)(H_2O)_5]^{3+}$ hs	8.7563	8.7500
{45}	$[Fe(AG_B)(H_2O)_5]^{3+}$ hs	8.7566	8.7500
{46}	$[Fe(AG_C)(H_2O)_5]^{3+}$ hs	8.7567	8.7500
{47}	$[Fe(AG_D)(H_2O)_5]^{3+}$ hs	8.7563	8.7500
{48}	$[Fe(AG_A)_2(H_2O)_2]^{3+}$ hs (5-coord.)	8.7584	8.7500
{49}	$[Fe(AG_A)_2(H_2O)_2]^{3+}$ hs (4-coord.)	8.7583	8.7500
{50}	$[Fe(AG_A)(H_2O)_4]^{3+}$ hs (5-coord.)	8.7570	8.7500
{51}	$[Fe(AG_B)_2(H_2O)_2]^{3+}$ hs (4-coord.)	8.7585	8.7500
{52}	$[Fe(AG_B)(H_2O)_4]^{3+}$ hs (5-coord.)	8.7571	8.7500
{53}	$[Fe(AG_C)_3]^{3+}$ hs (5-coord.)	8.7603	8.7500
{54}	$[Fe(AG_C)_2(H_2O)_2]^{3+}$ hs (4-coord.)	8.7588	8.7500
{55}	$[Fe(AG_C)(H_2O)_4]^{3+}$ hs (5-coord.)	8.7570	8.7500
{56}	$[Fe(AG_D)(H_2O)_2]^{3+}$ hs (4-coord.)	8.7579	8.7500
{57}	$[Fe(AG_D)(H_2O)_3]^{3+}$ hs (5-coord., sq. pyr.)	8.7568	8.7500
{58}	$[Fe(AG_D)(H_2O)_3]^{3+}$ hs (5-coord., tr. bipy.)	8.7575	8.7500
{59}	$[Fe(AG_D)_2]^{3+}$ hs (4-coord.)	8.7592	8.7500
{60}	$[Fe(AG_D)_2(H_2O)]^{3+}$ hs (5-coord.)	8.7581	8.7500
{62}	$[Fe(AG_D)_3]^{2+}$ hs (same orientation)	6.0164	6.0000
{63}	$[Fe(AG_D)_3]^{2+}$ hs	6.0166	6.0000
{64}	$[Fe(AG_D)_3(H_2O)]^{2+}$ hs	6.0168	6.0000

{65}	$[Fe(AG_D)_2(H_2O)_2]^{2+}$ hs (cis)	6.0139	6.0000
{66}	$[Fe(AG_D)_2(H_2O)_2]^{2+}$ hs (mirror image)	6.0158	6.0000
{67}	$[Fe(AG_D)_2(H_2O)_2]^{2+}$ hs (trans)	6.0155	6.0000
{68}	$[Fe(AG_A)_2(H_2O)_2]^{2+}$ hs (4-coord.)	6.0124	6.0000
{69}	$[Fe(AG_B)_2(H_2O)_2]^{2+}$ hs (4-coord.)	6.0129	6.0000
{70}	$[Fe(AG_C)_2(H_2O)_2]^{2+}$ hs (4-coord.)	6.0125	6.0000
{71}	$[Fe(AG_D)(H_2O)_3]^{2+}$ hs (5-coord., sq. pyr.)	6.0132	6.0000
{72}	$[Fe(AG_D)_2(H_2O)]^{2+}$ hs (5-coord.)	6.0157	6.0000
{73}	$[Fe(AG_D)(H_2O)_5]^{2+}$ hs	6.0108	6.0000
{74}	$[Fe(AG_B)(H_2O)_5]^{2+}$ hs	6.0118	6.0000
{75}	$[Fe(AG_D)(H_2O)_4]^{2+}$ hs	6.0118	6.0000
{76}	$[Fe(AG_A)(H_2O)_4]^{2+}$ hs (5-coord.)	6.0114	6.0000
{77}	$[Fe(AG_D)_2]^{2+}$ hs (4-coord.)	6.0138	6.0000
{78}	$[Fe(H_2O)_6]^{3+}$ hs	8.7545	8.7500
{79}	$[Fe(H_2O)_6]^{3+}$ ls	1.5091	1.1460
{80}	$[Fe(H_2O)_6]^{2+}$ hs	6.0104	6.0000

Table S3. Standard Gibbs free energy change (ΔG°_{f}, in kcal/mol) and formation constant (K_f, $logK_f$) for the chelation of Fe(III) with AGH^+ (as per Equation 5) in aqueous solution at 298.15 K.[a,b]

COMPLEX $[Fe(AGH)_x(H_2O)_n]^{(3+x)+}$	$\Delta G^{\circ}_{f_{Fe^{3+}-AGH^+}}$	$K_{f_{Fe^{3+}-AGH^+}}$	$logK_{f_{Fe^{3+}-AGH^+}}$
{1} $[Fe(AGH)_3]^{6+}$ hs (N2, N4)	76.5	8.43×10^{-57}	-56.07
{2} $[Fe(AGH)_3]^{6+}$ ls (N2, N4)	116.3	5.94×10^{-86}	-85.23
{3} $[Fe(AGH)_2(H_2O)_2]^{5+}$ hs (N2, N4)	46.6	7.09×10^{-35}	-34.15
{4} $[Fe(AGH)_2(H_2O)_2]^{5+}$ ls (N2, N4)	98.6	5.62×10^{-73}	-72.25
{5} $[Fe(AGH)(H_2O)_4]^{4+}$ hs (N2, N4)	27.5	7.25×10^{-21}	-20.14
{6} $[Fe(AGH)(H_2O)_4]^{4+}$ ls (N2, N4)	88.3	1.94×10^{-65}	-64.71
{7} $[Fe(AGH)(H_2O)_5]^{4+}$ hs (N4)	15.7	3.03×10^{-12}	-11.52
{8} $[Fe(AGH)_2(H_2O)_4]^{5+}$ hs (N4)	21.6	1.44×10^{-16}	-15.84
{9} $[Fe(AGH)_2]^{5+}$ hs (N2, N4)	57.6	5.87×10^{-43}	-42.23
{10} $[Fe(AGH)_2]^{5+}$ ls (N2, N4)	138.6	2.72×10^{-102}	-101.57

[a] Coordinating atoms in the organic ligand are shown in parentheses for each complex, and high- (hs) and low-spin (ls) complexes are identified.
[b] Formation equilibrium considered: $x AGH^+ + [Fe(H_2O)_6]^{3+} \leftrightarrows [Fe(AGH)_x(H_2O)_n]^{(3+x)+} + (6-n)H_2O$

Table S4. Standard Gibbs free energy change (ΔG_f°) and formation constant (K_f, $logK_f$) for the calculated Fe(III) complexes with AG (as per Equation 6) in aqueous solution at 298.15 K.[a,b]

COMPLEX $[Fe(AG)_x(H_2O)_n]^{3+}$	$\Delta G^{\circ}_{f_{Fe^{3+}-AG}}$ (kcal/mol)	$K_{f_{Fe^{3+}-AG}}$	$logK_{f_{Fe^{3+}-AG}}$
{11} $[Fe(AG_B)_3]^{3+}$ hs (N_1, N_4)	12.9	3.75×10^{-10}	-9.43
{12} $[Fe(AG_B)_3]^{3+}$ hs (same orientation, N_1, N_4)	14.4	2.56×10^{-11}	-10.59
{13} $[Fe(AG_C)_3]^{3+}$ hs (N_1, N_4)	11.1	7.53×10^{-9}	-8.12
{14} $[Fe(AG_D)_3]^{3+}$ hs (N_2, N_4)	-53.6	1.80×10^{39}	39.26
{15} $[Fe(AG_D)_3]^{3+}$ ls (N_2, N_4)	-23.5	1.64×10^{17}	17.21
{16} $[Fe(AG_D)_3]^{3+}$ hs (same orientation, N_2, N_4)	-54.6	1.13×10^{40}	40.05
{17} $[Fe(AG_D)_3(H_2O)]^{3+}$ hs (N_2, N_4, N_2', N_4', N_2'')	-40.9	9.91×10^{29}	30.00
{18} $[Fe(AG_D)_3(H_2O)_2]^{3+}$ hs (N_2, N_4, N_2', N_2'')	-35.9	2.05×10^{26}	26.31
{19} $[Fe(AG_A)_2(H_2O)_2]^{3+}$ ls (N_1, N_2)	50.5	9.51×10^{-38}	-37.02
{20} $[Fe(AG_A)_2(H_2O)_2]^{3+}$ ls (mirror image, N_1, N_2)	50.9	4.72×10^{-38}	-37.33
{21} $[Fe(AG_A)_2(H_2O)_4]^{3+}$ hs (N_2)	-21.5	5.99×10^{15}	15.78
{22} $[Fe(AG_B)_2(H_2O)_2]^{3+}$ hs (N_1, N_4)	14.0	5.19×10^{-11}	-10.28
{23} $[Fe(AG_B)_2(H_2O)_2]^{3+}$ ls (N_1, N_4)	54.6	1.02×10^{-40}	-39.99
{24} $[Fe(AG_B)_2(H_2O)_2]^{3+}$ hs (mirror image, N_1, N_4)	14.4	2.92×10^{-11}	-10.53
{25} $[Fe(AG_B)_2(H_2O)_2]^{3+}$ ls (mirror image, N_1, N_4)	54.0	2.54×10^{-40}	-39.60
{26} $[Fe(AG_B)_2(H_2O)_4]^{3+}$ hs (N_2)	-20.6	1.31×10^{15}	15.12
{27} $[Fe(AG_C)_2(H_2O)_2]^{3+}$ hs (N_1, N_4)	13.7	8.88×10^{-11}	-10.05
{28} $[Fe(AG_C)_2(H_2O)_2]^{3+}$ ls (N_1, N_4)	53.3	8.19×10^{-40}	-39.09
{29} $[Fe(AG_C)_2(H_2O)_2]^{3+}$ hs (mirror image, N_1, N_4)	14.2	4.14×10^{-11}	-10.38
{30} $[Fe(AG_C)_2(H_2O)_2]^{3+}$ ls (mirror image, N_1, N_4)	53.1	1.12×10^{-39}	-38.95
{31} $[Fe(AG_C)_2(H_2O)_4]^{3+}$ hs (N_2)	-21.1	3.16×10^{15}	15.50
{32} $[Fe(AG_D)_2(H_2O)_2]^{3+}$ hs (trans, N_2, N_4)	-31.3	8.48×10^{22}	22.93
{33} $[Fe(AG_D)_2(H_2O)_2]^{3+}$ ls (N_2, N_4)	9.2	1.66×10^{-7}	-6.78
{34} $[Fe(AG_D)_2(H_2O)_2]^{3+}$ hs (mirror image, N_2, N_4)	-30.1	1.25×10^{22}	22.10
{35} $[Fe(AG_D)_2(H_2O)_2]^{3+}$ ls (mirror image, N_2, N_4)	11.3	5.00×10^{-9}	-8.30
{36} $[Fe(AG_C)(AG_D)(H_2O)_2]^{3+}$ hs (N_1, N_4, N_2', N_4')	-9.4	8.05×10^{6}	6.91
{37} $[Fe(AG_C)(AG_D)(H_2O)_2]^{3+}$ ls (N_1, N_4, N_2', N_4')	30.1	9.36×10^{-23}	-22.03
{38} $[Fe(AG_D)_2(H_2O)_2]^{3+}$ hs (cis, N_2, N_4)	-34.1	9.29×10^{24}	24.97
{39} $[Fe(AG_D)_2(H_2O)_3]^{3+}$ hs (N_2, N_4, N_2')	-24.7	1.34×10^{18}	18.13
{40} $[Fe(AG_D)_2(H_2O)_4]^{3+}$ hs (N_2)	-15.3	1.69×10^{11}	11.23
{41} $[Fe(AG_B)(H_2O)_4]^{3+}$ hs (N_1, N_4)	6.6	1.47×10^{-5}	-4.83
{42} $[Fe(AG_C)(H_2O)_4]^{3+}$ hs (N_1, N_4)	5.8	6.02×10^{-5}	-4.22
{43} $[Fe(AG_D)(H_2O)_4]^{3+}$ hs (N_2, N_4)	-15.9	4.38×10^{11}	11.64
{44} $[Fe(AG_A)(H_2O)_5]^{3+}$ hs (N_2)	-10.8	8.47×10^{7}	7.93
{45} $[Fe(AG_B)(H_2O)_5]^{3+}$ hs (N_2)	-11.1	1.38×10^{8}	8.14
{46} $[Fe(AG_C)(H_2O)_5]^{3+}$ hs (N_2)	-10.7	6.41×10^{7}	7.81
{47} $[Fe(AG_D)(H_2O)_5]^{3+}$ hs (N_2)	-8.5	1.58×10^{6}	6.20

{48} $[Fe(AG_A)_2(H_2O)_2]^{3+}$ hs (5-coord., N_1, N_2, N_2')	-10.5	5.04×10^{7}	7.70
{49} $[Fe(AG_A)_2(H_2O)_2]^{3+}$ hs (4-coord., N_2)	-30.8	3.48×10^{22}	22.54
{50} $[Fe(AG_A)(H_2O)_4]^{3+}$ hs (5-coord., N_2)	-13.7	1.14×10^{10}	10.06
{51} $[Fe(AG_B)_2(H_2O)_2]^{3+}$ hs (4-coord., N_2)	-32.7	1.01×10^{24}	24.00
{52} $[Fe(AG_B)(H_2O)_4]^{3+}$ hs (5-coord., N_2)	-15.9	4.70×10^{11}	11.67
{53} $[Fe(AG_C)_3]^{3+}$ hs (5-coord., N_1, N_4, N_1', N_4', N_4'')	14.0	5.30×10^{-11}	-10.28
{54} $[Fe(AG_C)_2(H_2O)_2]^{3+}$ hs (4-coord., N_2)	-31.7	1.73×10^{23}	23.24
{55} $[Fe(AG_C)(H_2O)_4]^{3+}$ hs (5-coord., N_2)	-17.2	3.93×10^{12}	12.59
{56} $[Fe(AG_D)(H_2O)_2]^{3+}$ hs (4-coord., N_2, N_4)	-16.4	9.92×10^{11}	12.00
{57} $[Fe(AG_D)(H_2O)_3]^{3+}$ hs (5-coord., sq. pyr., N_2, N_4)	-20.9	2.06×10^{15}	15.31
{58} $[Fe(AG_D)(H_2O)_3]^{3+}$ hs (5-coord., tr. bipy., N_2, N_4)	-15.5	2.27×10^{11}	11.36
{59} $[Fe(AG_D)_2]^{3+}$ hs (4-coord., N_2, N_4)	-31.7	1.62×10^{23}	23.21
{60} $[Fe(AG_D)_2(H_2O)]^{3+}$ hs (5-coord., N_2, N_4)	-34.7	2.82×10^{25}	25.45

[a] The octahedral complexes are grouped according to the number of AG ligands present (3, 2 or 1), followed by the non-octahedral complexes calculated; coordinating atoms in the organic ligand are shown in parentheses for each complex, and high- (hs) and low-spin (ls) complexes are identified.

[b] Formation equilibrium considered: xAG + $[Fe(H_2O)_6]^{3+}$ ⇆ $[Fe(AG)_x(H_2O)_n]^{3+}$ + (6-n)H_2O

Table S5. Standard Gibbs free energy change (ΔG_f°, in kcal/mol) and formation constant (K_f, $logK_f$) for the calculated Fe(III) complexes with AG (as per Equation 7) in aqueous solution at 298.15 K, taking into account the ΔG° to form AG from AGH^+ at physiological pH.[a,b]

COMPLEX $[Fe(AG)_x(H_2O)_n]^{3+}$	$\Delta G^{\circ}_{f_{Fe^{3+}-AG}}$	$K_{f_{Fe^{3+}-AG}}$	$logK_{f_{Fe^{3+}-AG}}$
{11} $[Fe(AG_B)_3]^{3+}$ hs (N_1, N_4)	29.6	1.88×10^{-22}	-21.73
{12} $[Fe(AG_B)_3]^{3+}$ hs (same orientation, N_1, N_4)	31.2	1.28×10^{-23}	-22.89
{13} $[Fe(AG_C)_3]^{3+}$ hs (N_1, N_4)	27.9	3.78×10^{-21}	-20.42
{14} $[Fe(AG_D)_3]^{3+}$ hs (N_2, N_4)	-36.8	9.03×10^{26}	26.96
{15} $[Fe(AG_D)_3]^{3+}$ ls (N_2, N_4)	-6.7	8.22×10^{4}	4.91
{16} $[Fe(AG_D)_3]^{3+}$ hs (same orientation, N_2, N_4)	-37.9	5.65×10^{27}	27.75
{17} $[Fe(AG_D)_3(H_2O)]^{3+}$ hs (N_2, N_4, N_2', N_4', N_2'')	-24.1	4.96×10^{17}	17.70
{18} $[Fe(AG_D)_3(H_2O)_2]^{3+}$ hs (N_2, N_4, N_2', N_2'')	-19.1	1.03×10^{14}	14.01
{19} $[Fe(AG_A)_2(H_2O)_2]^{3+}$ ls (N_1, N_2)	61.7	6.00×10^{-46}	-45.22
{20} $[Fe(AG_A)_2(H_2O)_2]^{3+}$ ls (mirror image, N_1, N_2)	62.1	2.98×10^{-46}	-45.53
{21} $[Fe(AG_A)_2(H_2O)_4]^{3+}$ hs (N_2)	-10.3	3.78×10^{7}	7.58
{22} $[Fe(AG_B)_2(H_2O)_2]^{3+}$ hs (N_1, N_4)	25.2	3.27×10^{-19}	-18.48
{23} $[Fe(AG_B)_2(H_2O)_2]^{3+}$ ls (N_1, N_4)	65.7	6.44×10^{-49}	-48.19
{24} $[Fe(AG_B)_2(H_2O)_2]^{3+}$ hs (mirror image, N_1, N_4)	25.6	1.84×10^{-19}	-18.73
{25} $[Fe(AG_B)_2(H_2O)_2]^{3+}$ ls (mirror image, N_1, N_4)	65.2	1.60×10^{-48}	-47.80

{26} $[Fe(AG_B)_2(H_2O)_4]^{3+}$ hs (N_2)	-9.4	8.29 x 10^{6}	6.92
{27} $[Fe(AG_C)_2(H_2O)_2]^{3+}$ hs (N_1, N_4)	24.9	5.60 x 10^{-19}	-18.25
{28} $[Fe(AG_C)_2(H_2O)_2]^{3+}$ ls (N_1, N_4)	64.5	5.17 x 10^{-48}	-47.29
{29} $[Fe(AG_C)_2(H_2O)_2]^{3+}$ hs (mirror image, N_1, N_4)	25.4	2.61 x 10^{-19}	-18.58
{30} $[Fe(AG_C)_2(H_2O)_2]^{3+}$ ls (mirror image, N_1, N_4)	64.3	7.04 x 10^{-48}	-47.15
{31} $[Fe(AG_C)_2(H_2O)_4]^{3+}$ hs (N_2)	-10.0	1.99 x 10^{7}	7.30
{32} $[Fe(AG_D)_2(H_2O)_2]^{3+}$ hs (trans, N_2, N_4)	-20.1	5.35 x 10^{14}	14.73
{33} $[Fe(AG_D)_2(H_2O)_2]^{3+}$ ls (N_2, N_4)	20.4	1.05 x 10^{-15}	-14.98
{34} $[Fe(AG_D)_2(H_2O)_2]^{3+}$ hs (mirror image, N_2, N_4)	-19.0	7.91 x 10^{13}	13.90
{35} $[Fe(AG_D)_2(H_2O)_2]^{3+}$ ls (mirror image, N_2, N_4)	22.5	3.15 x 10^{-17}	-16.50
{36} $[Fe(AG_C)(AG_D)(H_2O)_2]^{3+}$ hs (N_1, N_4, N_2', N_4')	1.8	5.08 x 10^{-2}	-1.29
{37} $[Fe(AG_C)(AG_D)(H_2O)_2]^{3+}$ ls (N_1, N_4, N_2', N_4')	41.2	5.91 x 10^{-31}	-30.23
{38} $[Fe(AG_D)_2(H_2O)_2]^{3+}$ hs (cis, N_2, N_4)	-22.9	5.86 x 10^{16}	16.77
{39} $[Fe(AG_D)_2(H_2O)_3]^{3+}$ hs (N_2, N_4, N_2')	-13.5	8.45 x 10^{9}	9.93
{40} $[Fe(AG_D)_2(H_2O)_4]^{3+}$ hs (N_2)	-4.1	1.06 x 10^{3}	3.03
{41} $[Fe(AG_B)(H_2O)_4]^{3+}$ hs (N_1, N_4)	12.2	1.17 x 10^{-9}	-8.93
{42} $[Fe(AG_C)(H_2O)_4]^{3+}$ hs (N_1, N_4)	11.4	4.78 x 10^{-9}	-8.32
{43} $[Fe(AG_D)(H_2O)_4]^{3+}$ hs (N_2, N_4)	-10.3	3.48 x 10^{7}	7.54
{44} $[Fe(AG_A)(H_2O)_5]^{3+}$ hs (N_2)	-5.2	6.73 x 10^{3}	3.83
{45} $[Fe(AG_B)(H_2O)_5]^{3+}$ hs (N_2)	-5.5	1.10 x 10^{4}	4.04
{46} $[Fe(AG_C)(H_2O)_5]^{3+}$ hs (N_2)	-5.1	5.09 x 10^{3}	3.71
{47} $[Fe(AG_D)(H_2O)_5]^{3+}$ hs (N_2)	-2.9	1.25 x 10^{2}	2.10
{48} $[Fe(AG_A)_2(H_2O)_2]^{3+}$ hs (5-coord., N_1, N_2, N_2')	0.7	3.18 x 10^{-1}	-0.50
{49} $[Fe(AG_A)_2(H_2O)_2]^{3+}$ hs (4-coord., N_2)	-19.6	2.20 x 10^{14}	14.34
{50} $[Fe(AG_A)(H_2O)_4]^{3+}$ hs (5-coord., N_2)	-8.1	9.04 x 10^{5}	5.96
{51} $[Fe(AG_B)_2(H_2O)_2]^{3+}$ hs (4-coord., N_2)	-21.6	6.35 x 10^{15}	15.80
{52} $[Fe(AG_B)(H_2O)_4]^{3+}$ hs (5-coord., N_2)	-10.3	3.73 x 10^{7}	7.57
{53} $[Fe(AG_C)_3]^{3+}$ hs (5-coord., N_1, N_4, N_1', N_4', N_4'')	30.8	2.66 x 10^{-23}	-22.58
{54} $[Fe(AG_C)_2(H_2O)_2]^{3+}$ hs (4-coord., N_2)	-20.5	1.09 x 10^{15}	15.04
{55} $[Fe(AG_C)(H_2O)_4]^{3+}$ hs (5-coord., N_2)	-11.6	3.12 x 10^{8}	8.49
{56} $[Fe(AG_D)(H_2O)_2]^{3+}$ hs (4-coord., N_2, N_4)	-10.8	7.88 x 10^{7}	7.90
{57} $[Fe(AG_D)(H_2O)_3]^{3+}$ hs (5-coord., sq. pyr., N_2, N_4)	-15.3	1.63 x 10^{11}	11.21
{58} $[Fe(AG_D)(H_2O)_3]^{3+}$ hs (5-coord., tr. bipy., N_2, N_4)	-9.9	1.80 x 10^{7}	7.26
{59} $[Fe(AG_D)_2]^{3+}$ hs (4-coord., N_2, N_4)	-20.5	1.02 x 10^{15}	15.01
{60} $[Fe(AG_D)_2(H_2O)]^{3+}$ hs (5-coord., N_2, N_4)	-23.5	1.78 x 10^{17}	17.25

[a] The octahedral complexes are grouped according to the number of AG ligands present (3, 2 or 1), followed by the non-octahedral complexes calculated; coordinating atoms in the organic ligand are shown in parentheses for each complex, and high- (hs) and low-spin (ls) complexes are identified.

[b] Formation equilibrium considered: $x AGH^{+} + [Fe(H_2O)_6]^{3+} \leftrightarrows [Fe(AG)_x(H_2O)_n]^{3+} + (6-n)H_2O + xH^{+}$

Table S6. Standard Gibbs free energy of reaction (ΔG°, kcal/mol) and activation (ΔG≠, kcal/mol), various rate constants (k, k_D and k_{app}, M^{-1} s^{-1}) and the rate constant ratio (using k_{app} for the reduction of $[Fe(H_2O)_6]^{3+}$ as reference) for the initial reaction of the Haber-Weiss cycle (with and without iron complexation with AG) with $O_2^{\bullet-}$ in aqueous solution at 298.15 K.[a]

Reaction	ΔG⁰	ΔG≠	k	k_D	k_{app}	Ratio
$[Fe(H_2O)_6]^{3+} + O_2^{\bullet-} \rightarrow [Fe(H_2O)_6]^{2+} + O_2$	-37.9	2.2	1.42 x 10^{11}	7.67 x 10^9	7.28 x 10^9	
{15}$[Fe(AG_D)_3]^{3+} + O_2^{\bullet-} \rightarrow$ {61}$[Fe(AG_D)_3]^{2+} + O_2$ (ls)	-6.5	2.0	2.16 x 10^{11}	8.05 x 10^9	7.76 x 10^9	0.94
{16}$[Fe(AG_D)_3]^{3+} + O_2^{\bullet-} \rightarrow$ {62}$[Fe(AG_D)_3]^{2+} + O_2$	-11.5	1.0	1.12 x 10^{12}	8.15 x 10^9	8.09 x 10^9	0.90
{14}$[Fe(AG_D)_3]^{3+} + O_2^{\bullet-} \rightarrow$ {63}$[Fe(AG_D)_3]^{2+} + O_2$	-14.2	0.8	1.61 x 10^{12}	8.33 x 10^9	8.29 x 10^9	0.88
{17}$[Fe(AG_D)_3(H_2O)]^{3+} + O_2^{\bullet-} \rightarrow$ {64}$[Fe(AG_D)_3(H_2O)]^{2+} + O_2$	-24.6	0.4	3.16 x 10^{12}	8.32 x 10^9	8.30 x 10^9	0.88
{38}$[Fe(AG_D)_2(H_2O)_2]^{3+} + O_2^{\bullet-} \rightarrow$ {65}$[Fe(AG_D)_2(H_2O)_2]^{2+} + O_2$	-21.6	0.001	6.21 x 10^{12}	8.09 x 10^9	8.08 x 10^9	0.90
{34}$[Fe(AG_D)_2(H_2O)_2]^{3+} + O_2^{\bullet-} \rightarrow$ {66}$[Fe(AG_D)_2(H_2O)_2]^{2+} + O_2$	-23.4	0.01	6.10 x 10^{12}	8.03 x 10^9	8.02 x 10^9	0.91
{32}$[Fe(AG_D)_2(H_2O)_2]^{3+} + O_2^{\bullet-} \rightarrow$ {67}$[Fe(AG_D)_2(H_2O)_2]^{2+} + O_2$	-22.8	0.008	6.13 x 10^{12}	8.00 x 10^9	7.99 x 10^9	0.91
{49}$[Fe(AG_A)_2(H_2O)_2]^{3+} + O_2^{\bullet-} \rightarrow$ {68}$[Fe(AG_A)_2(H_2O)_2]^{2+} + O_2$	-25.7	0.04	5.77 x 10^{12}	8.07 x 10^9	8.06 x 10^9	0.90
{51}$[Fe(AG_B)_2(H_2O)_2]^{3+} + O_2^{\bullet-} \rightarrow$ {69}$[Fe(AG_B)_2(H_2O)_2]^{2+} + O_2$	-24.8	0.1	5.14 x 10^{12}	7.90 x 10^9	7.89 x 10^9	0.92
{54}$[Fe(AG_C)_2(H_2O)_2]^{3+} + O_2^{\bullet-} \rightarrow$ {70}$[Fe(AG_C)_2(H_2O)_2]^{2+} + O_2$	-24.9	0.3	3.93 x 10^{12}	8.12 x 10^9	8.10 x 10^9	0.90
{57}$[Fe(AG_D)(H_2O)_3]^{3+} + O_2^{\bullet-} \rightarrow$ {71}$[Fe(AG_D)(H_2O)_3]^{2+} + O_2$	-30.4	0.6	2.43 x 10^{12}	7.70 x 10^9	7.67 x 10^9	0.95
{60}$[Fe(AG_D)_2(H_2O)]^{3+} + O_2^{\bullet-} \rightarrow$ {72}$[Fe(AG_D)_2(H_2O)]^{2+} + O_2$	-25.7	0.3	3.52 x 10^{12}	8.11 x 10^9	8.09 x 10^9	0.90
{47}$[Fe(AG_D)(H_2O)_5]^{3+} + O_2^{\bullet-} \rightarrow$ {73}$[Fe(AG_D)(H_2O)_5]^{2+} + O_2$	-28.6	0.4	2.93 x 10^{12}	7.94 x 10^9	7.92 x 10^9	0.92
{45}$[Fe(AG_B)(H_2O)_5]^{3+} + O_2^{\bullet-} \rightarrow$ {74}$[Fe(AG_B)(H_2O)_5]^{2+} + O_2$	-31.9	0.3	3.84 x 10^{12}	8.07 x 10^9	8.05 x 10^9	0.90
{43}$[Fe(AG_D)(H_2O)_4]^{3+} + O_2^{\bullet-} \rightarrow$ {75}$[Fe(AG_D)(H_2O)_4]^{2+} + O_2$	-31.2	0.5	2.52 x 10^{12}	7.96 x 10^9	7.94 x 10^9	0.92
{50}$[Fe(AG_A)(H_2O)_4]^{3+} + O_2^{\bullet-} \rightarrow$ {76}$[Fe(AG_A)(H_2O)_4]^{2+} + O_2$	-32.6	0.5	2.71 x 10^{12}	7.93 x 10^9	7.91 x 10^9	0.92
{59}$[Fe(AG_D)_2]^{3+} + O_2^{\bullet-} \rightarrow$ {77}$[Fe(AG_D)_2]^{2+} + O_2$	-27.0	1.8	2.96 x 10^{11}	7.96 x 10^9	7.75 x 10^9	0.94

[a] The iron complexes are high-spin, unless otherwise indicated (ls = low spin)

Table S7. Standard Gibbs free energy of reaction (ΔG°, kcal/mol) and activation (ΔG≠, kcal/mol), various rate constants (k, k_D and k_{app}, M^{-1} s^{-1}) and the rate constant ratio (using k_{app} for the reduction of $[Fe(H_2O)_6]^{3+}$ as reference) for the initial reaction of the Haber-Weiss cycle (with and without iron complexation with AG) with ascorbate (ASC^-) in aqueous solution at 298.15 K.[a]

Reaction	ΔG⁰	ΔG≠	k	k_D	k_{app}	Ratio
$[Fe(H_2O)_6]^{3+} + ASC^- \rightarrow [Fe(H_2O)_6]^{2+} + ASC^\bullet$	-14.8	0.3	3.71 x 10^{12}	7.45 x 10^9	7.43 x 10^9	
**{15}**$[Fe(AG_D)_3]^{3+} + ASC^- \rightarrow$ **{61}**$[Fe(AG_D)_3]^{2+} + ASC^\bullet$ (ls)	16.5	16.6	4.48			1.66 x 10^9
**{16}**$[Fe(AG_D)_3]^{3+} + ASC^- \rightarrow$ **{62}**$[Fe(AG_D)_3]^{2+} + ASC^\bullet$	11.6	12.0	9.47 x 10^3			7.85 x 10^5
**{14}**$[Fe(AG_D)_3]^{3+} + ASC^- \rightarrow$ **{63}**$[Fe(AG_D)_3]^{2+} + ASC^\bullet$	8.9	10.2	1.96 x 10^5			3.79 x 10^4
**{17}**$[Fe(AG_D)_3(H_2O)]^{3+} + ASC^- \rightarrow$ **{64}**$[Fe(AG_D)_3(H_2O)]^{2+} + ASC^\bullet$	-1.6	6.3	1.59 x 10^8	4.11 x 10^9	1.53 x 10^8	48.6
**{38}**$[Fe(AG_D)_2(H_2O)_2]^{3+} + ASC^- \rightarrow$ **{65}**$[Fe(AG_D)_2(H_2O)_2]^{2+} + ASC^\bullet$	1.5	5.3	7.89 x 10^8	3.91 x 10^9	6.57 x 10^8	11.3
**{34}**$[Fe(AG_D)_2(H_2O)_2]^{3+} + ASC^- \rightarrow$ **{66}**$[Fe(AG_D)_2(H_2O)_2]^{2+} + ASC^\bullet$	-0.4	5.0	1.31 x 10^9	3.86 x 10^9	9.81 x 10^8	7.57
**{32}**$[Fe(AG_D)_2(H_2O)_2]^{3+} + ASC^- \rightarrow$ **{67}**$[Fe(AG_D)_2(H_2O)_2]^{2+} + ASC^\bullet$	0.3	4.7	2.21 x 10^9	7.43 x 10^9	1.70 x 10^9	4.37
**{49}**$[Fe(AG_A)_2(H_2O)_2]^{3+} + ASC^- \rightarrow$ **{68}**$[Fe(AG_A)_2(H_2O)_2]^{2+} + ASC^\bullet$	-2.6	3.8	1.05 x 10^{10}	3.90 x 10^9	2.84 x 10^9	2.62
**{51}**$[Fe(AG_B)_2(H_2O)_2]^{3+} + ASC^- \rightarrow$ **{69}**$[Fe(AG_B)_2(H_2O)_2]^{2+} + ASC^\bullet$	-1.7	3.7	1.24 x 10^{10}	3.74 x 10^9	2.87 x 10^9	2.59
**{54}**$[Fe(AG_C)_2(H_2O)_2]^{3+} + ASC^- \rightarrow$ **{70}**$[Fe(AG_C)_2(H_2O)_2]^{2+} + ASC^\bullet$	-1.8	3.3	2.50 x 10^{10}	3.94 x 10^9	3.40 x 10^9	2.18
**{57}**$[Fe(AG_D)(H_2O)_3]^{3+} + ASC^- \rightarrow$ **{71}**$[Fe(AG_D)(H_2O)_3]^{2+} + ASC^\bullet$	-7.3	1.9	2.47 x 10^{11}	3.53 x 10^9	3.48 x 10^9	2.13
**{60}**$[Fe(AG_D)_2(H_2O)]^{3+} + ASC^- \rightarrow$ **{72}**$[Fe(AG_D)_2(H_2O)]^{2+} + ASC^\bullet$	-2.6	3.0	4.04 x 10^{10}	3.93 x 10^9	3.59 x 10^9	2.07
**{47}**$[Fe(AG_D)(H_2O)_5]^{3+} + ASC^- \rightarrow$ **{73}**$[Fe(AG_D)(H_2O)_5]^{2+} + ASC^\bullet$	-5.5	2.3	1.28 x 10^{11}	7.42 x 10^9	7.01 x 10^9	1.06
**{45}**$[Fe(AG_B)(H_2O)_5]^{3+} + ASC^- \rightarrow$ **{74}**$[Fe(AG_B)(H_2O)_5]^{2+} + ASC^\bullet$	-8.8	2.1	1.72 x 10^{11}	7.44 x 10^9	7.13 x 10^9	1.04
**{43}**$[Fe(AG_D)(H_2O)_4]^{3+} + ASC^- \rightarrow$ **{75}**$[Fe(AG_D)(H_2O)_4]^{2+} + ASC^\bullet$	-8.1	1.8	2.79 x 10^{11}	7.42 x 10^9	7.23 x 10^9	1.03
**{50}**$[Fe(AG_A)(H_2O)_4]^{3+} + ASC^- \rightarrow$ **{76}**$[Fe(AG_A)(H_2O)_4]^{2+} + ASC^\bullet$	-9.6	1.7	3.35 x 10^{11}	7.42 x 10^9	7.26 x 10^9	1.02
**{59}**$[Fe(AG_D)_2]^{3+} + ASC^- \rightarrow$ **{77}**$[Fe(AG_D)_2]^{2+} + ASC^\bullet$	-3.9	1.5	5.19 x 10^{11}	7.42 x 10^9	7.32 x 10^9	1.01

[a] The iron complexes are high-spin, unless otherwise indicated (ls = low spin)

Table S8. Standard Gibbs free energy of reaction (ΔG°, kcal/mol) and activation (ΔG≠, kcal/mol), various rate constants (k, k_D and k_{app}, M^{-1} s^{-1}) and the rate constant ratio (using k_{app} for the reduction of $[Cu(H_2O)_4]^{2+}$ as reference) for the initial reaction of the Haber-Weiss cycle (with and without copper complexation with AG or AGH^+) with ascorbate (ASC^-) in aqueous solution at 298.15 K.[a]

Reaction	ΔG⁰	ΔG≠	k	k_D	k_{app}	Ratio
$[Cu(H_2O)_4]^{2+} + ASC^- \rightarrow [Cu(H_2O)_2]^+ \cdot 2H_2O + ASC^\bullet$	-9.5	4.5	2.92 x 10^9	7.43 x 10^9	2.10 x 10^9	1
{4}$[Cu(AGH)(H_2O)_3]^{3+} + ASC^- \rightarrow$ {31}$[Cu(AGH)(H_2O)]^{2+} \cdot 2H_2O + ASC^\bullet$	-10.3	3.9	8.84 x 10^9	7.44 x 10^9	4.04 x 10^9	0.520
{9}$[Cu(AG_C)(H_2O)_2]^{2+} + ASC^- \rightarrow$ {32}$[Cu(AG_C)(H_2O)]^+ \cdot H_2O + ASC^\bullet$	-2.6	6.1	1.99 x 10^8	7.44 x 10^9	1.94 x 10^8	10.8
{10}$[Cu(AG_C)(H_2O)_3]^{2+} + ASC^- \rightarrow$ {33}$[Cu(AG_C)(H_2O)]^+ \cdot 2H_2O + ASC^\bullet$	-8.3	5.5	5.58 x 10^8	7.42 x 10^9	5.19 x 10^8	4.05
{22}$[Cu(AG_C)_2(H_2O)_2]^{2+} + ASC^- \rightarrow$ {34}$[Cu(AG_C)_2]^+ \cdot 2H_2O + ASC^\bullet$	-6.3	5.9	2.92 x 10^8	7.43 x 10^9	2.81 x 10^8	7.47
{14}$[Cu(AG_A)_2(H_2O)_2]^{2+} + ASC^- \rightarrow$ {35}$[Cu(AG_A)_2]^+ \cdot 2H_2O + ASC^\bullet$	-7.9	5.8	3.56 x 10^8	7.42 x 10^9	3.40 x 10^8	6.18
{20}$[Cu(AG_C)_2]^{2+} + ASC^- \rightarrow$ {36}$[Cu(AG_C)_2]^+ + ASC^\bullet$ [a]	-1.2	7.2	3.47 x 10^7			60.5
{19}$[Cu(AG_C)_2]^{2+} + ASC^- \rightarrow$ {37}$[Cu(AG_C)_2]^+ + ASC^\bullet$	-2.0	7.1	3.81 x 10^7			55.1
{11}$[Cu(AG_D)(H_2O)_2]^{2+} + ASC^- \rightarrow$ {38}$[Cu(AG_D)(H_2O)]^+ \cdot H_2O + ASC^\bullet$	3.0	9.7	4.83 x 10^5			4.35 x 10^3
{29}$[Cu(AG_C)(AG_D)]^{2+} + ASC^- \rightarrow$ {39}$[Cu(AG_C)(AG_D)]^+ + ASC^\bullet$	4.9	10.8	8.01 x 10^4			2.62 x 10^4
{25}$[Cu(AG_D)_2(H_2O)]^{2+} + ASC^- \rightarrow$ {40}$[Cu(AG_D)_2]^+ \cdot H_2O + ASC^\bullet$	3.9	11.2	3.56 x 10^4			5.90 x 10^4
{23}$[Cu(AG_D)_2]^{2+} + ASC^- \rightarrow$ {41}$[Cu(AG_D)_2]^+ + ASC^\bullet$	12.0	15.0	66.6			3.15 x 10^7
{24}$[Cu(AG_D)_2]^{2+} + ASC^- \rightarrow$ {42}$[Cu(AG_D)_2]^+ + ASC^\bullet$	11.5	15.0	60.9			3.45 x 10^7

[a] The labelling system for the copper complexes has been taken from Reference 23.

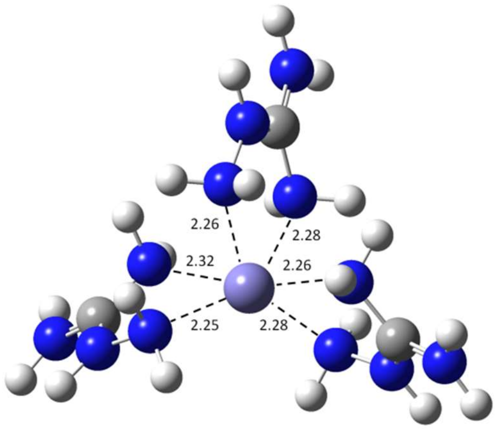

{1} $[Fe(AGH)_3]^{6+}$ hs

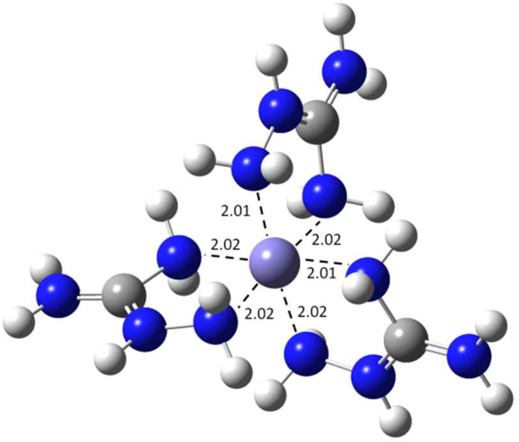

{2} $[Fe(AGH)_3]^{6+}$ ls

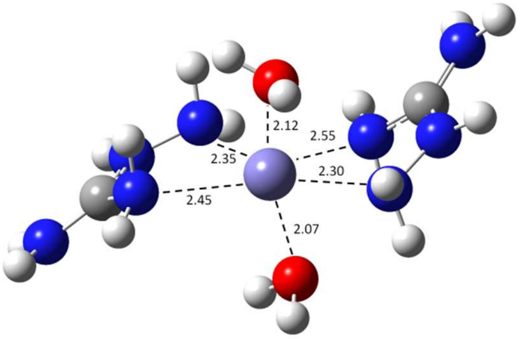

{3} $[Fe(AGH)_2(H_2O)_2]^{5+}$ hs

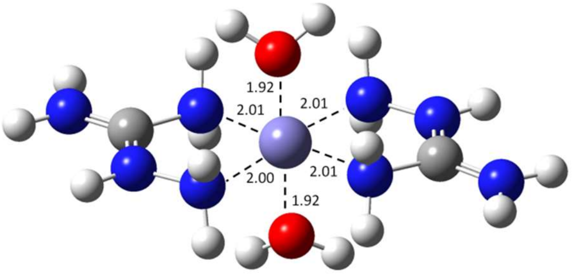

{4} $[Fe(AGH)_2(H_2O)_2]^{5+}$ ls

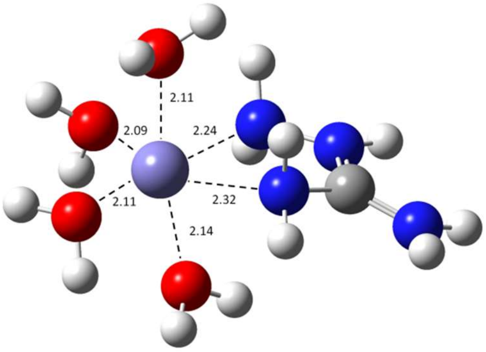

{5} $[Fe(AGH)(H_2O)_4]^{4+}$ hs

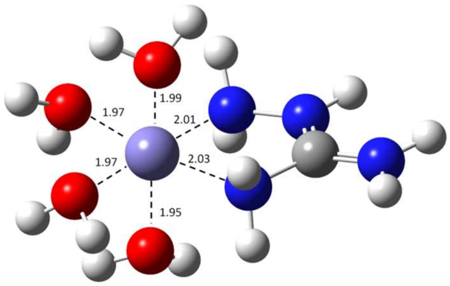

{6} $[Fe(AGH)(H_2O)_4]^{4+}$ ls

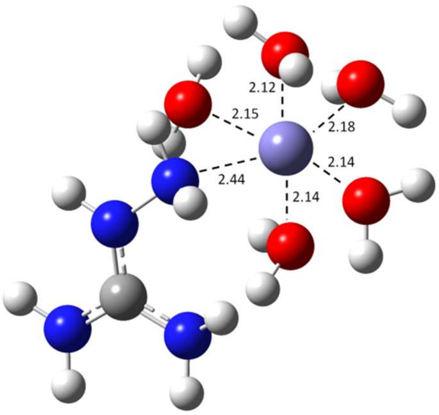

{7} $[Fe(AGH)(H_2O)_5]^{4+}$ hs

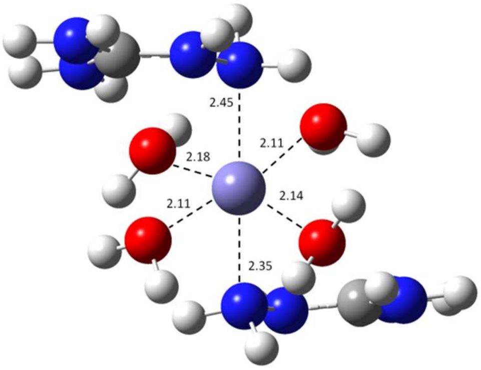

{8} $[Fe(AGH)_2(H_2O)_4]^{5+}$ hs

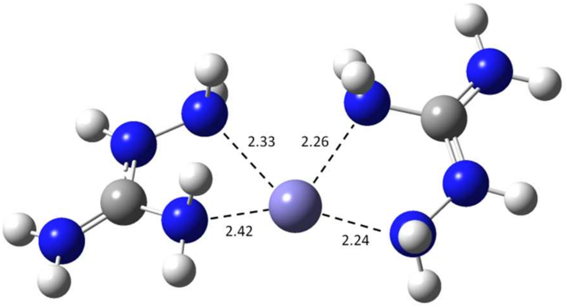

{9} $[Fe(AGH)_2]^{5+}$ hs

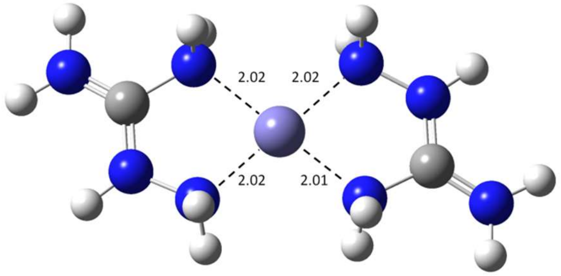

{10} $[Fe(AGH)_2]^{5+}$ ls

Figure S1. Optimized geometries of complexes of Fe(III) with AGH^{+} in aqueous solution (bond distances in Å); high- (hs) and low-spin (ls) complexes are identified.

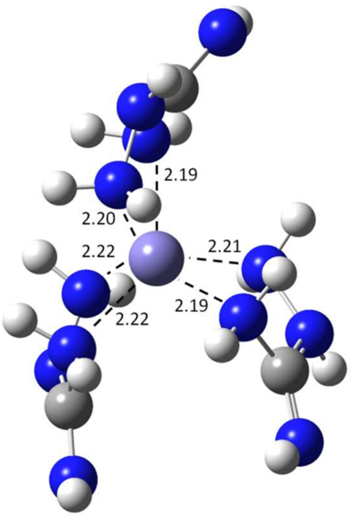

{11} $[Fe(AG_B)_3]^{3+}$ hs

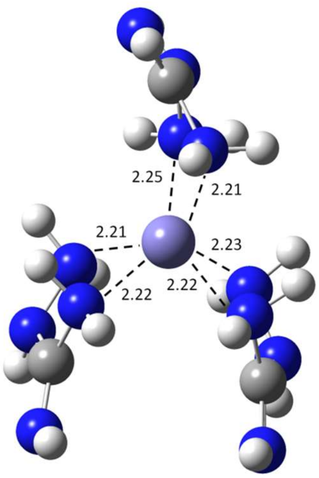

{12} $[Fe(AG_B)_3]^{3+}$ hs (same orientation)

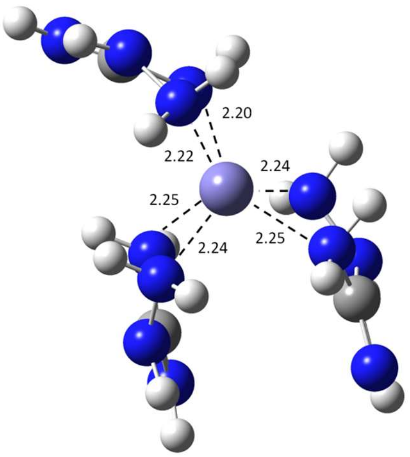

{13} $[Fe(AG_C)_3]^{3+}$ hs

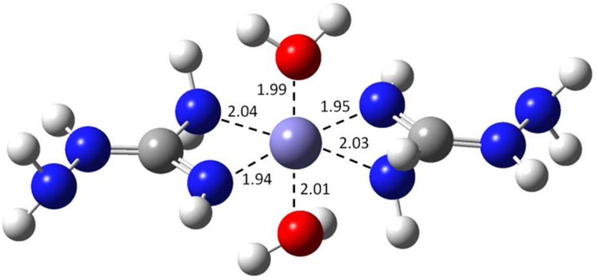

{19} $[Fe(AG_A)_2(H_2O)_2]^{3+}$ ls

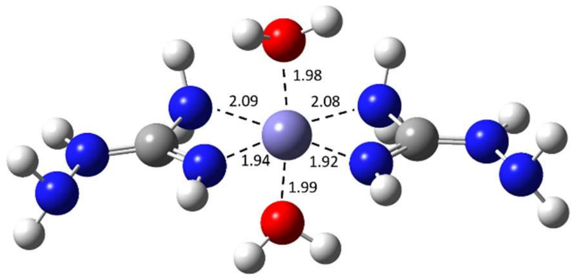

{20} $[Fe(AG_A)_2(H_2O)_2]^{3+}$ ls (mirror image)

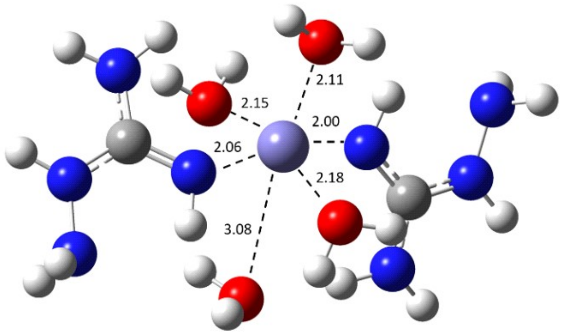

{21} $[Fe(AG_A)_2(H_2O)_4]^{3+}$ hs

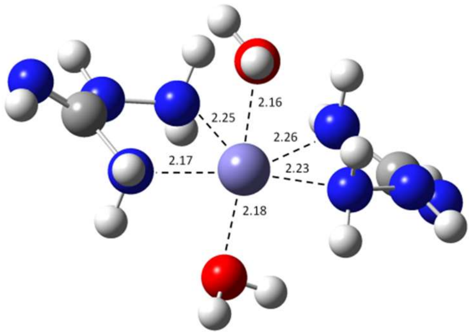

{22} $[Fe(AG_B)_2(H_2O)_2]^{3+}$ hs

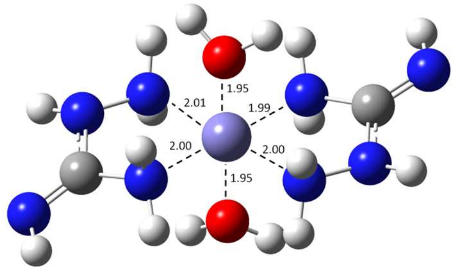

{23} $[Fe(AG_B)_2(H_2O)_2]^{3+}$ ls

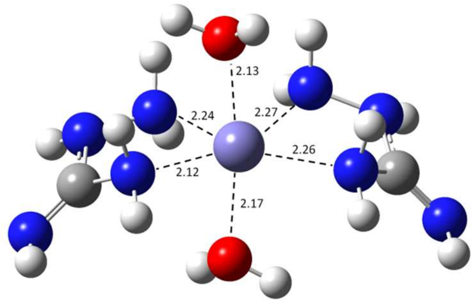

{24} $[Fe(AG_B)_2(H_2O)_2]^{3+}$ hs (mirror image)

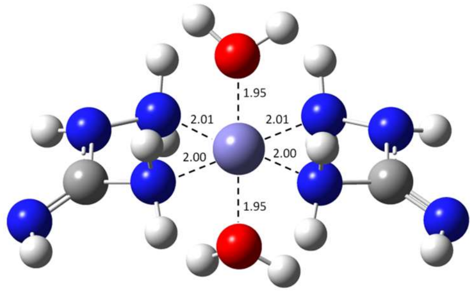

{25} $[Fe(AG_B)_2(H_2O)_2]^{3+}$ ls (mirror image)

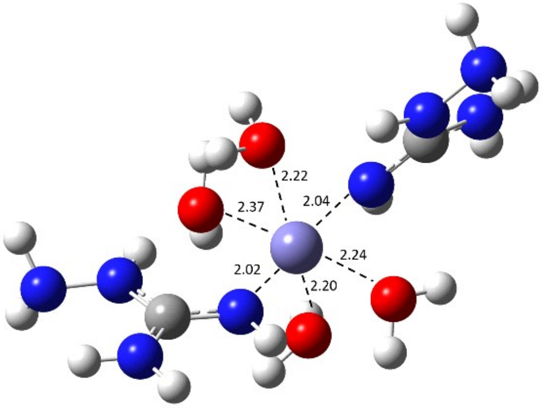

{26} $[Fe(AG_B)_2(H_2O)_4]^{3+}$ hs

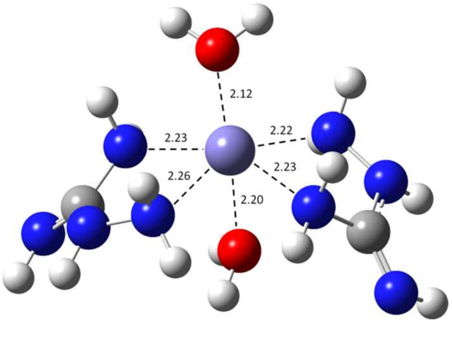

{27} $[Fe(AG_C)_2(H_2O)_2]^{3+}$ hs

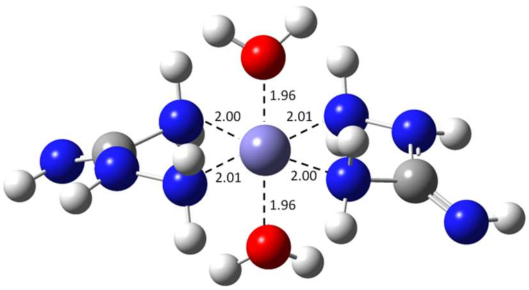

{28} $[Fe(AG_C)_2(H_2O)_2]^{3+}$ ls

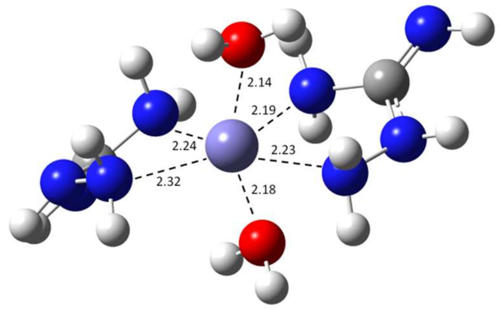

{29} $[Fe(AG_C)_2(H_2O)_2]^{3+}$ hs (mirror image)

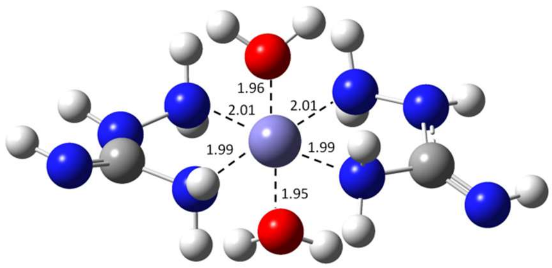

{30} $[Fe(AG_C)_2(H_2O)_2]^{3+}$ ls (mirror image)

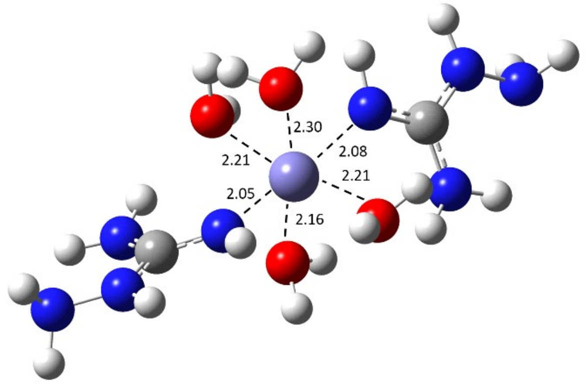

{31} $[Fe(AG_C)_2(H_2O)_4]^{3+}$ hs

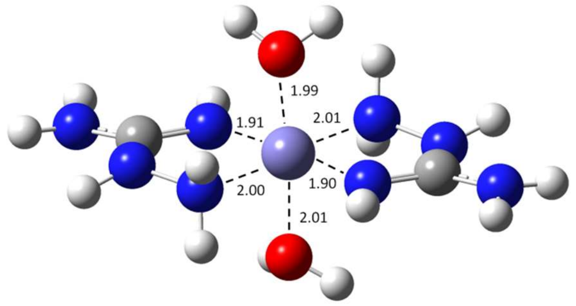

{33} $[Fe(AG_D)_2(H_2O)_2]^{3+}$ ls

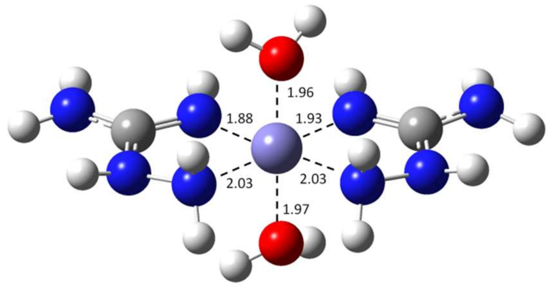

{35} $[Fe(AG_D)_2(H_2O)_2]^{3+}$ ls (mirror image)

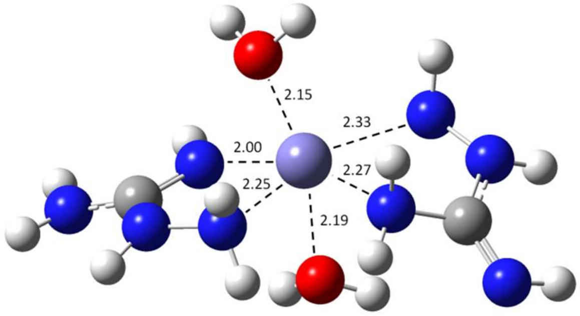

{36} $[Fe(AG_C)(AG_D)(H_2O)_2]^{3+}$ hs

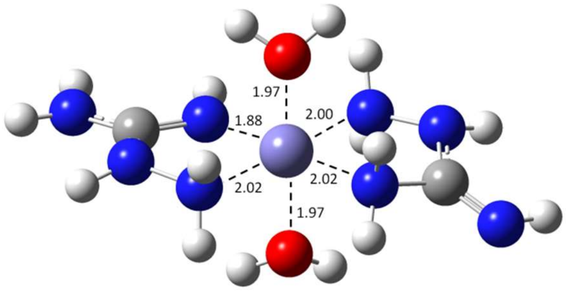

{37} $[Fe(AG_C)(AG_D)(H_2O)_2]^{3+}$ ls

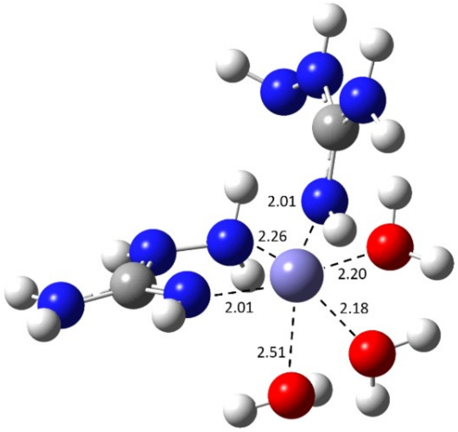

{39} $[Fe(AG_D)_2(H_2O)_3]^{3+}$ hs

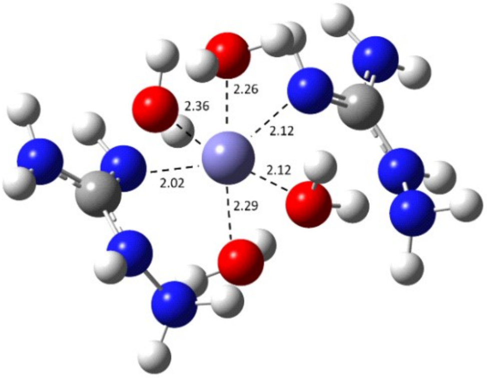

{40} $[Fe(AG_D)_2(H_2O)_4]^{3+}$ hs

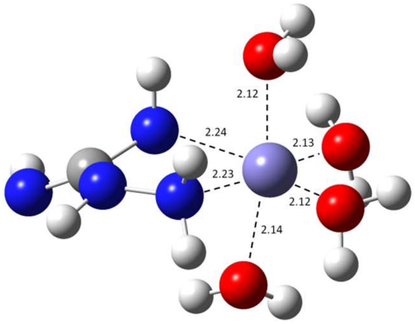

{41} $[Fe(AG_B)(H_2O)_4]^{3+}$ hs

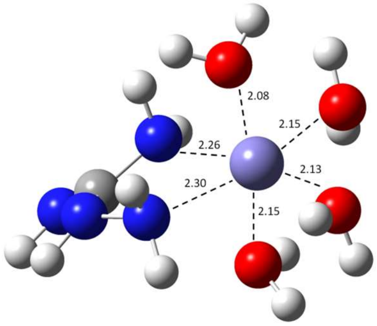

{42} $[Fe(AG_C)(H_2O)_4]^{3+}$ hs

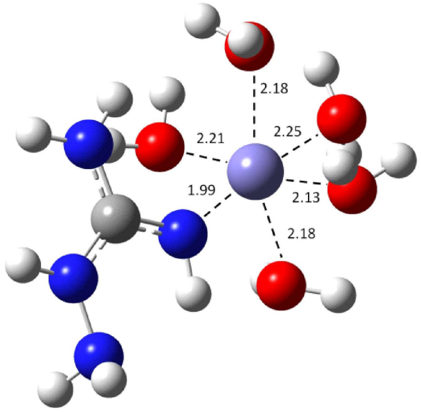

{44} $[Fe(AG_A)(H_2O)_5]^{3+}$ hs

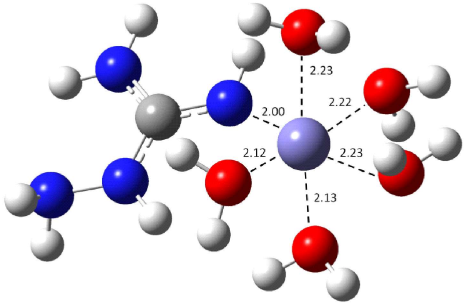

{45} $[Fe(AG_B)(H_2O)_5]^{3+}$ hs

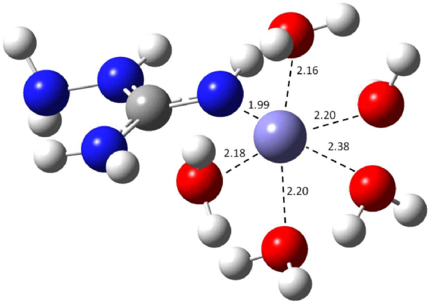

{46} $[Fe(AG_C)(H_2O)_5]^{3+}$ hs

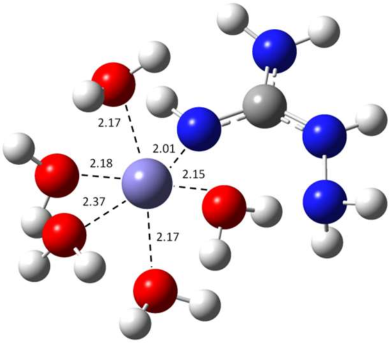

{47} $[Fe(AG_D)(H_2O)_5]^{3+}$ hs

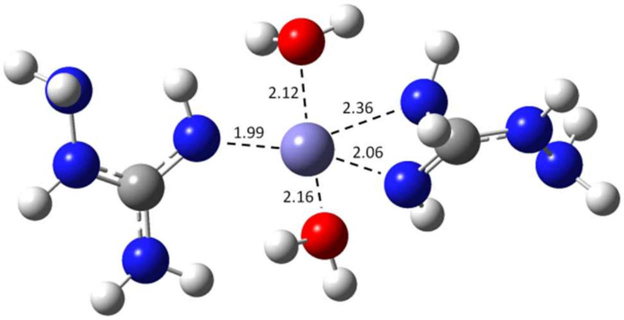

{48} $[Fe(AG_A)_2(H_2O)_2]^{3+}$ hs (5-coord.)

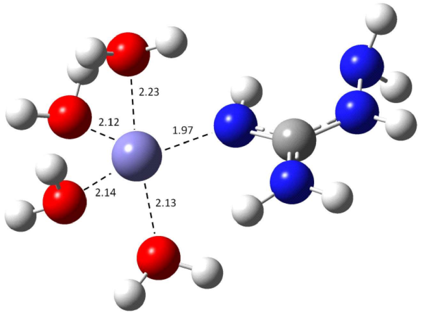

{50} $[Fe(AG_A)(H_2O)_4]^{3+}$ hs (5-coord.)

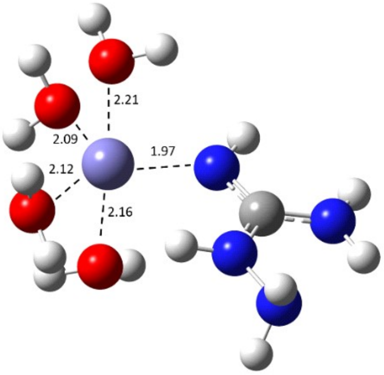

{52} $[Fe(AG_B)(H_2O)_4]^{3+}$ hs (5-coord.)

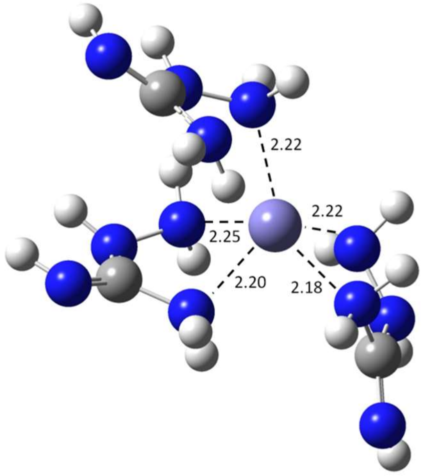

{53} $[Fe(AG_C)_3]^{3+}$ hs (5-coord.)

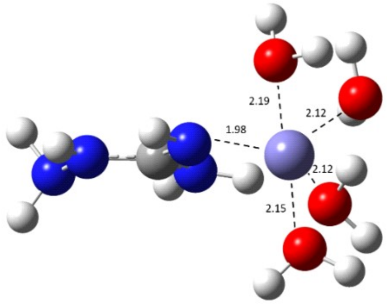

{55} $[Fe(AG_C)(H_2O)_4]^{3+}$ hs (5-coord.)

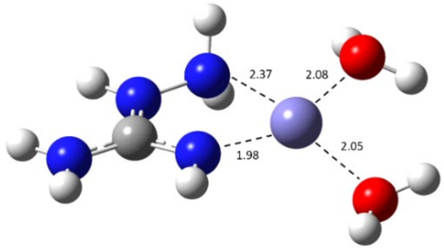

{56} $[Fe(AG_D)(H_2O)_2]^{3+}$ hs (4-coord.)

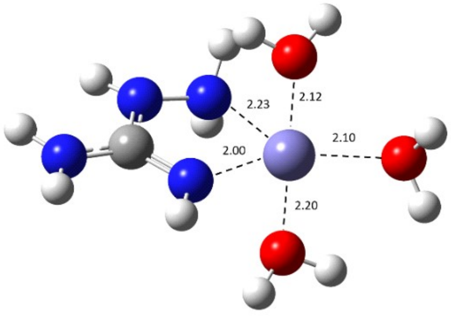

{58} $[Fe(AG_D)(H_2O)_3]^{3+}$ hs (5-coord., tr. bipy.)

Figure S2. Optimized geometries of the 1:3, 1:2 and 1:1 Fe(III) complexes with AG in aqueous solution that are not listed in Table 1 (bond distances in Å); high- (hs) and low-spin (ls) complexes are identified.

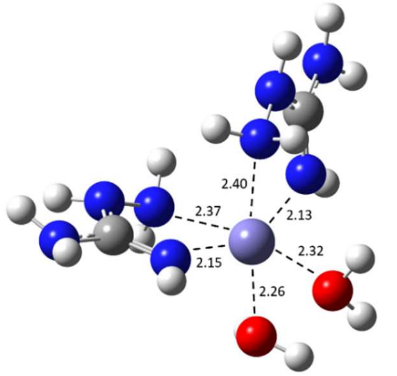

{38} → {65} $[Fe(AG_D)_2(H_2O)_2]^{2+}$ hs (cis)

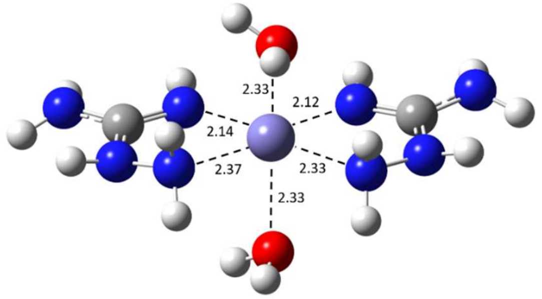

{34} → {66} $[Fe(AG_D)_2(H_2O)_2]^{2+}$ hs (mirror)

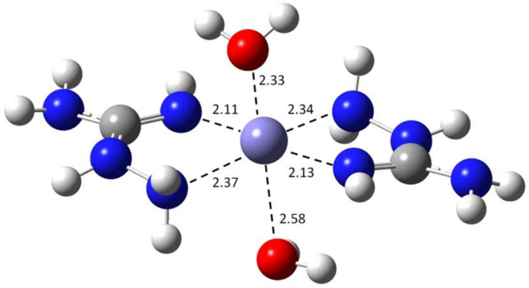

{32} → {67} $[Fe(AG_D)_2(H_2O)_2]^{2+}$ hs (trans)

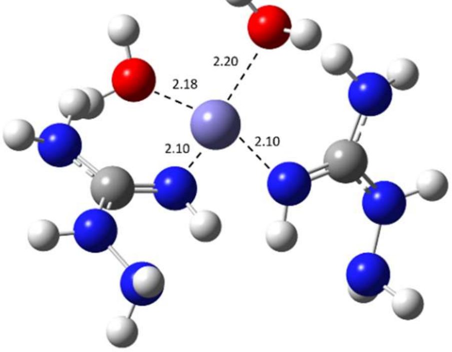

{49} → {68} $[Fe(AG_A)_2(H_2O)_2]^{2+}$ hs (4-coord.)

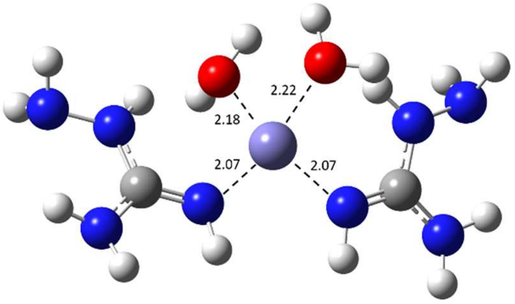

{51} → {69} $[Fe(AG_B)_2(H_2O)_2]^{2+}$ hs (4-coord.)

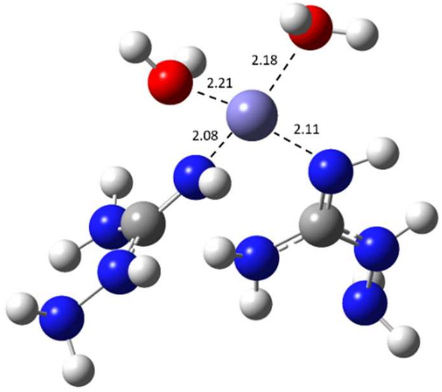

{54} → {70} $[Fe(AG_C)_2(H_2O)_2]^{2+}$ hs (4-coord.)

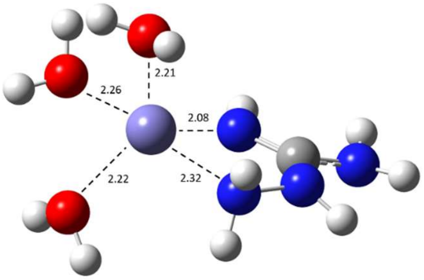

{57} → {71} $[Fe(AG_D)(H_2O)_3]^{2+}$ hs (5-coord., sq. pyr.)

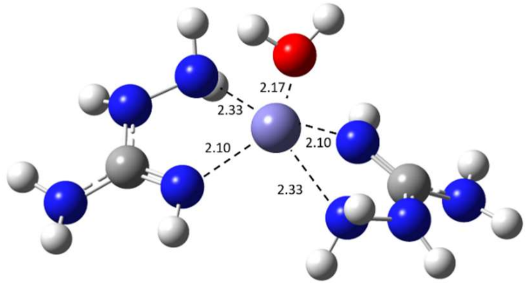

{60} → {72} $[Fe(AG_D)_2(H_2O)]^{2+}$ hs (5-coord.)

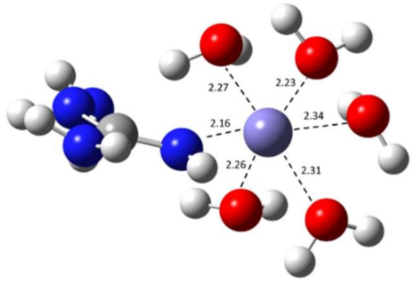

{47} → {73} $[Fe(AG_D)(H_2O)_5]^{2+}$ hs

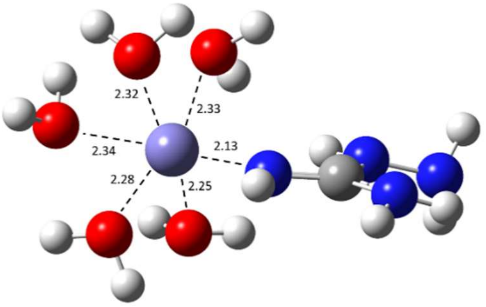

{45} → {74} $[Fe(AG_B)(H_2O)_5]^{2+}$ hs

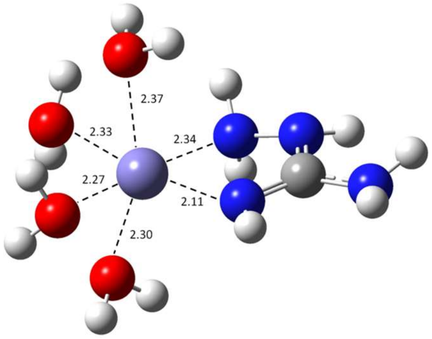

{43} → {75} $[Fe(AG_D)(H_2O)_4]^{2+}$ hs

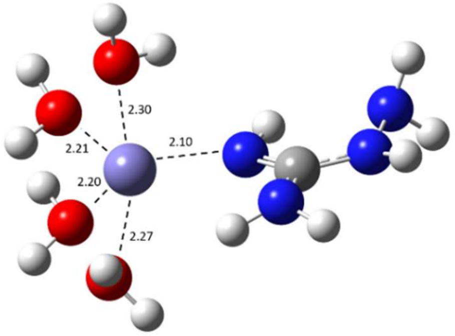

{50} → {76} $[Fe(AG_A)(H_2O)_4]^{2+}$ hs (5-coord.)

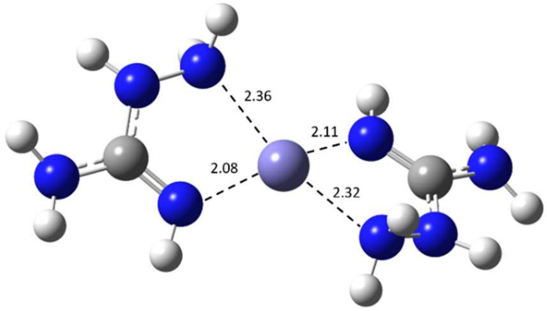

{59} → {77} $[Fe(AG_D)_2]^{2+}$ hs (4-coord.)

Figure S3. Optimized geometries of Fe(II) complexes with AG in aqueous solution that appear in Tables S6 and S7, but are not shown in Figure 3 (indicating the Fe(III) complex used as starting point in each case; bond distances in Å); these complexes are high spin (hs).

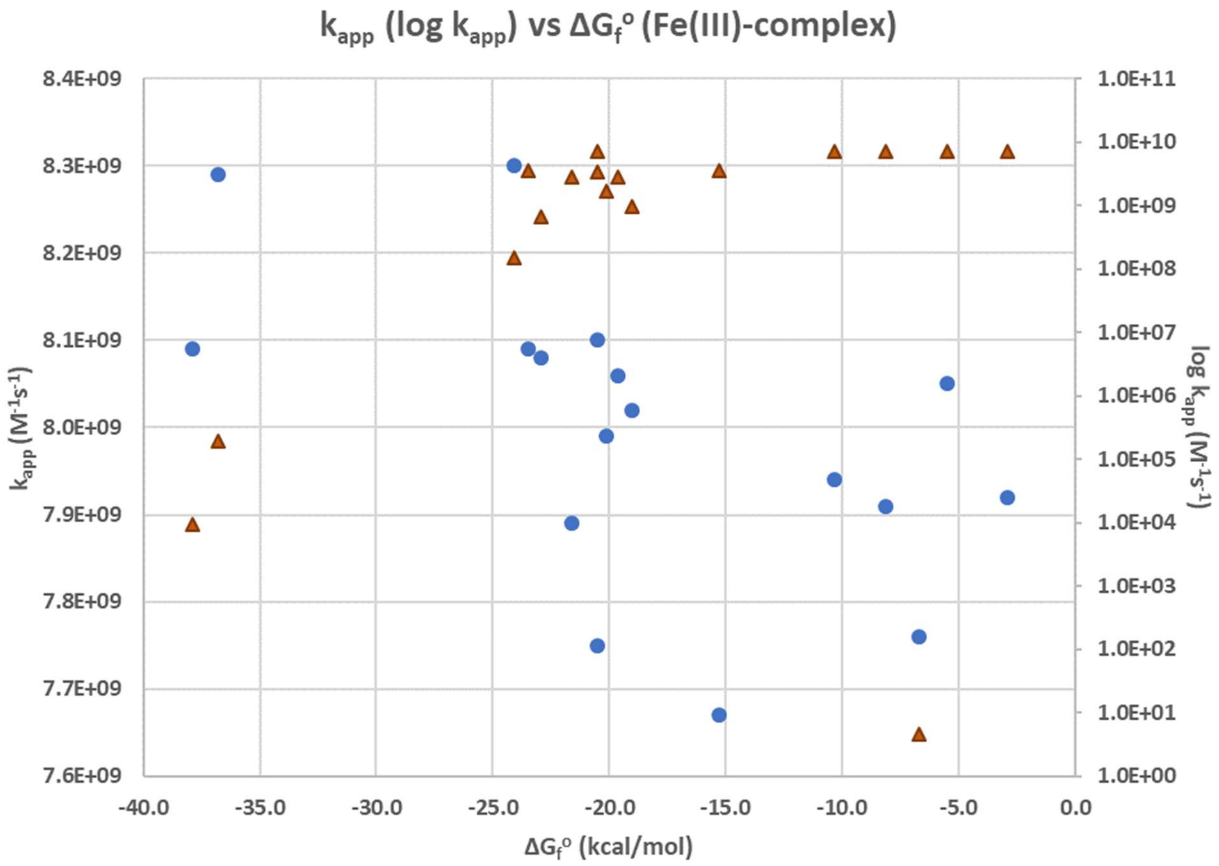

Figure S4. Plots of the apparent rate constant (k_{app}) for the SET reactions of Fe(III) complexes with $O_2^{\bullet-}$ (left axis: k_{app}; marker: circles) and ASC^- (right axis: log k_{app}; marker: triangles) *versus* the ΔG_f° of the Fe(III) complexes.

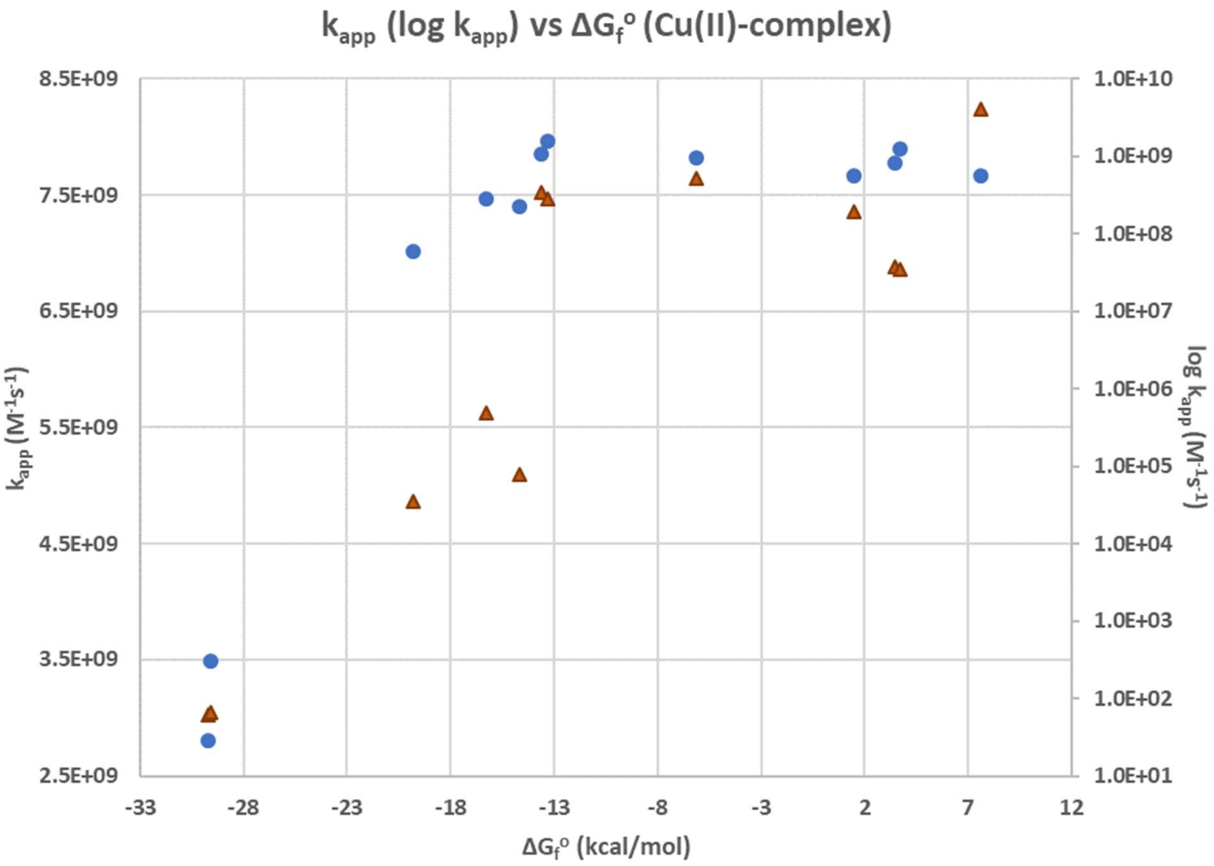

Figure S5. Plots of the apparent rate constant (k_{app}) for the SET reactions of Cu(II) complexes with $O_2^{\bullet-}$ (left axis: k_{app}; marker: circles) and ASC^- (right axis: log k_{app}; marker: triangles) *versus* the ΔG_f° of the Cu(II) complexes studied.

Appendix 1. Additional explanation on corrections to calculated Gibbs free energies in solution and the approach followed in this study.

When evaluating the change in Gibbs free energy in solution for thermodynamic or kinetic purposes, two types of corrections should be made. One of them deals with the change in the reference state. Since a Gaussian output file provides a standard Gibbs free energy value for the 1 atm reference state, even when calculations have been performed applying a continuum solvation method to simulate the solvent effect, this value should be converted to the 1 M reference state. This way, the calculated ΔG° for an equilibrium mathematically connects with a K_C-type of equilibrium constant. In addition, the free volume correction to liquid phase proposed by Benson (S. W. Benson, *The Foundations of Chemical Kinetics*, Krieger: Florida, **1982**) should also be included. Okuno was the first one to apply this correction (Y. Okuno, *Chem. – Eur. J.*, **1997**, *3*, 212–218). Benson's correction is a way to take into account the limitation to the free movement of species in the liquid phase (cage effect) in comparison to what happens in the gas phase (ideal gas model). Its use could be considered a way to account for the entropic effect of the presence of solvent molecules around a solute. For details on the mathematical expressions to be applied when making these corrections, see for example Ref. 39. As noted in Ref. 39, if simultaneously ignoring these two corrections in kinetic calculations, rate constants would be underestimated by about 1800 times for bimolecular reactions at room temperature. Large errors would also accompany thermodynamic calculations in solution. These two corrections are regularly applied in kinetic studies in solution involving a transition state such as the evaluation of primary antioxidant activity. See, for example, Refs. 22, 36-40, 50.

Because Benson's correction is accounted for in an approximate way, and to mathematically simplify our way of calculating the ΔG_f^0 of the various complexes, we have conceived each complex formation equilibrium having the same number of species in the reactant and product sides. In doing so, none of these two corrections are required for each species as they cancel out. In other words, clustering equivalent reactant or product species avoids corrections that when accounted for in an approximate way, could introduce additional errors in the calculation of the ΔG_f^0 of the various complexes and could affect our thermodynamic comparison of complexes with the same central ion. This is the way we have chosen to proceed as part of the methodology followed, which is consistent with previous publications from our group and others dealing with

thermodynamic studies of complex formation related to the evaluation of secondary antioxidant activity. See, for example, Refs. 23, 24, 35, 48-50.

Proceeding one way or another might affect the thermodynamic comparison between the various complexes with the same central ion, but it would not affect the comparison between equivalent Fe(III) and Cu(II) complexes or the kinetic results we have reported, since we have evaluated a significant set of Fe(III) and Cu(II) complexes and there is a clear relationship between the thermodynamic stability of these complexes (using the ΔG_f^0 values calculated as described), and the rate constant of their SET reactions. More importantly, we need to have a unified methodology if aiming at properly comparing the same set of antioxidants relative to their secondary antioxidant activity with respect to the Fe(III)/Fe(II) and Cu(II)/Cu(I) reductions. We have attempted to do so for lipoic and dihydrolipoic acids (see Refs. 24, 35) and for AG (see Ref. 23, and the current study).

In this study, we have successfully applied the methodology described to show that, in agreement with experimental results, complexes between AG and Cu(II) are able to slow down the rate constant of the oxidation of ascorbate. From our calculations, the same would be expected with Fe(III) complexes (see Table 2), and we have shown that the most stable complexes with Fe(III) and Cu(II) are the ones that regularly lead to very large rate constant reductions. The same was concluded in the study reported for dihydrolipoic acid in Ref. 24.

M05(SMD)/6-311+G(d,p) Cartesian coordinates of the optimized geometries in water of the species calculated in this study.

{1} $[Fe(AGH)_3]^{6+}$ hs

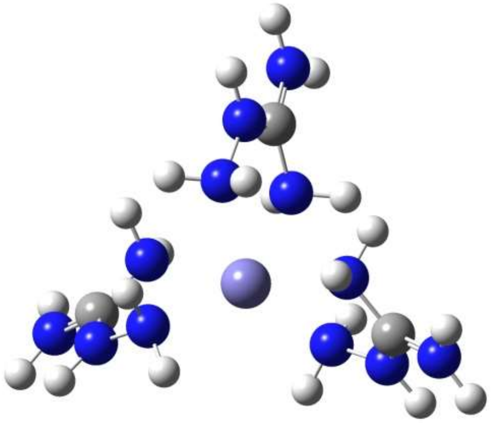

```
Charge =  6 Multiplicity = 6
 Fe,0,-0.0667535769,-0.4160784778,0.1222137425
 N,0,-0.001309834,1.288208787,1.6026530091
 H,0,-0.9140253525,1.3806458204,2.0550262482
 H,0,0.6704176443,1.0690326393,2.3411573672
 N,0,0.3286814018,2.5229419637,1.0581449955
 H,0,0.2954207793,3.3343302436,1.6702038619
 C,0,0.5351936637,2.6597247972,-0.2364712906
 N,0,0.7030772762,3.8216996512,-0.7945832773
 H,0,0.6477164766,4.6732212021,-0.2478420451
 H,0,0.9246377494,3.885642236,-1.7794394331
 N,0,0.5875393849,1.4661191536,-0.9926406441
 H,0,1.5579953311,1.3388180489,-1.2999345556
 N,0,1.0615142725,-1.3978212278,-1.5976227971
 H,0,1.2411819942,-0.7818121798,-2.3905558395
 H,0,0.4459800629,-2.1449351714,-1.9230111956
 N,0,2.2538223298,-1.9639071991,-1.1690452167
 C,0,2.7445759166,-1.6722476203,0.0200960938
 H,0,2.6988035366,-2.6580717502,-1.7633854876
 N,0,3.7690671968,-2.2957742442,0.5224207446
 H,0,4.2035213527,-3.0639795167,0.025190136
 H,0,4.1833998588,-1.9816165311,1.3900551172
 N,0,2.0972312415,-0.6383844602,0.7271039975
 H,0,2.1810301021,-0.7973737078,1.7332516292
 N,0,-1.6458571305,-1.3134946773,1.4457429228
 H,0,-1.7596466121,-0.8376322316,2.3418708596
 H,0,-1.3168644538,-2.2612186392,1.6359736579
 N,0,-2.8681017872,-1.399157394,0.7922752832
 H,0,-3.5318752136,-2.1055775045,1.0956639834
 C,0,-3.1205691721,-0.6153374241,-0.2400125402
 N,0,-4.1840002754,-0.750563139,-0.9767262995
 H,0,-4.844729868,-1.4979208059,-0.8025335019
 H,0,-4.3887565454,-0.0770107556,-1.702337959
 N,0,-2.1627556242,0.3725725987,-0.5040389558
 H,0,-2.360964293,1.1875891954,0.0842749749
 H,0,0.0462143683,1.5832885429,-1.8542475238
 H,0,2.5909367584,0.2391660567,0.5356713407
 H,0,-2.2162699596,0.679858721,-1.4755114027
```

{2} $[Fe(AGH)_3]^{6+}$ ls

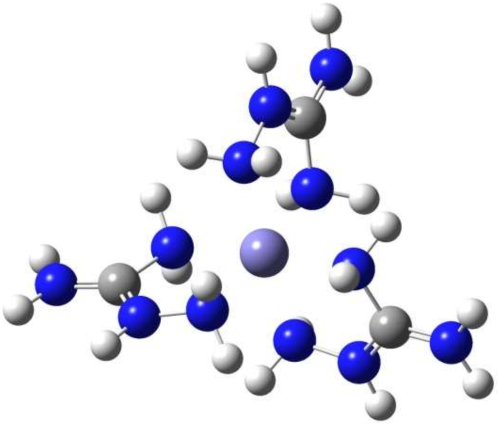

```
Charge =  6 Multiplicity = 2
 Fe,0,0.0768347002,0.2957481902,-0.0029903331
 N,0,-0.0472423312,2.3065923263,-0.0464477696
 H,0,0.4465587733,2.7517536267,0.732955287
 H,0,0.3848290793,2.6641615541,-0.9051092075
 N,0,-1.3770255072,2.7291924429,-0.0176268171
 H,0,-1.5579147488,3.7303810087,0.0004941659
 C,0,-2.3502119918,1.8444658794,-0.0053324216
 N,0,-3.6078045848,2.1548523061,0.008961633
 H,0,-3.9061336554,3.1239338007,0.0136297775
 H,0,-4.311805772,1.4273450976,0.035148328
 N,0,-1.938738902,0.4796419763,-0.01741415
 H,0,-2.3916216348,0.0105359187,-0.8121292236
 N,0,0.0648024929,-1.7190810289,-0.1147431567
```

```
 H,0,-0.7624064191,-2.0861845643,0.36751443
 H,0,0.8784296392,-2.145789613,0.3384386968
 N,0,0.0267074524,-2.1467002938,-1.4427972616
 C,0,0.0271374002,-1.2615498252,-2.416313737
 H,0,-0.0190312288,-3.1475160683,-1.6203102919
 N,0,-0.0418013745,-1.5644181012,-3.6735113562
 H,0,-0.1099551654,-2.5300925363,-3.9748551335
 H,0,-0.0297074704,-0.8336879638,-4.3750536399
 N,0,0.1105673998,0.1029298008,-2.0083631885
 H,0,0.9766330723,0.4989292597,-2.3969594545
 N,0,2.0884293969,0.2636142637,0.1704002607
 H,0,2.4879713793,1.1000215317,-0.2692590496
 H,0,2.5150171947,-0.5403850015,-0.2996883967
 N,0,2.4720043438,0.245908663,1.5121553203
 H,0,3.4671048666,0.2174072931,1.7232183599
 C,0,1.5600088064,0.3379856742,2.4570321908
 N,0,1.8341503726,0.4144582017,3.7202948373
 H,0,2.7942716156,0.4213927083,4.0456529376
 H,0,1.0876695001,0.4488147066,4.4037963069
 N,0,0.205557303,0.345568275,2.0093116409
 H,0,-0.277217381,1.1473784229,2.4327586944
 H,0,-2.3299026476,0.0173084132,0.8133024525
 H,0,-0.6407685595,0.6253748404,-2.47478019
 H,0,-0.2643064145,-0.4798821856,2.405180459
```

{3} $[Fe(AGH)_2(H_2O)_2]^{5+}$ hs

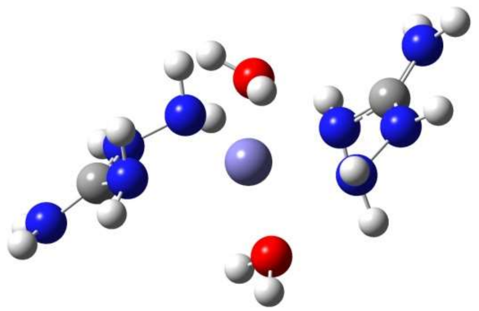

```
Charge =  5 Multiplicity = 6
 Fe,0,-0.0177556243,-0.5268425945,0.1570538044
 N,0,2.0732397747,-1.4455678143,-0.1368881387
 H,0,2.1622581235,-2.1870541398,0.5565394333
 H,0,2.1393769446,-1.8700955794,-1.0625029348
 N,0,1.728439618,1.0502070165,-0.8274549509
 H,0,1.5572389042,2.05058015,-0.8791876386
 C,0,2.9097338739,0.7392368888,-0.1919223896
 N,0,3.1122008268,-0.5457948498,0.0575834326
 H,0,3.9759234762,-0.8808869262,0.4699837061
 N,0,3.763995261,1.6562662286,0.1779421487
 H,0,3.6541026462,2.609334871,-0.1382553624
 H,0,4.5770356801,1.4153269323,0.728745165
 N,0,-1.1819906129,1.5170048994,0.1002137494
 H,0,-1.0359263489,1.9597145934,1.0087003397
 H,0,-0.7978550977,2.1316442387,-0.6144927491
 N,0,-2.5409304946,1.373641892,-0.1508507228
 C,0,-3.1288597955,0.2022860804,0.0330293707
 N,0,-2.3938358348,-0.750127941,0.7075366387
 N,0,-4.3297636051,-0.0456776312,-0.4161052422
 H,0,-4.8113961139,-0.8880216986,-0.1341128506
 H,0,-4.8290333631,0.6404299572,-0.966630265
 H,0,-2.803537325,-1.6786941251,0.662055061
 H,0,-3.0369891749,2.1513425065,-0.5727664125
 H,0,-2.2480982662,-0.4857789986,1.6841769459
 H,0,1.6706952835,0.6378728729,-1.7583689512
 O,0,-0.1278489164,-1.146139632,-1.8180346039
 H,0,-0.2783988231,-2.0970973999,-1.9190008105
 H,0,-0.8770352564,-0.7089651503,-2.2496070755
 O,0,0.6416493782,-0.5475165901,2.172621938
 H,0,0.8331115228,-1.4314475688,2.5164906372
 H,0,-0.0631576706,-0.1966924878,2.7353907176
```

{4} $[Fe(AGH)_2(H_2O)_2]^{5+}$ ls

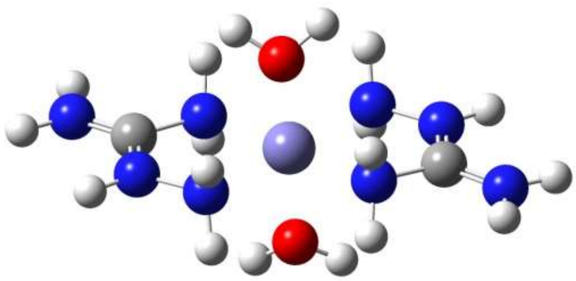

```
Charge =  5 Multiplicity = 2
 Fe,0,0.01351863,-0.0126231738,0.0369896039
 N,0,-1.2551671666,-1.5685274175,0.0504599133
 H,0,-1.0822980515,-2.1680840585,-0.7602281819
 H,0,-1.1229340757,-2.1303484211,0.8952693962
 N,0,-1.7012252912,1.0280717652,0.1234753933
 H,0,-1.7400966848,1.7441540641,-0.6094672513
 C,0,-2.832445421,0.1729319052,-0.00148231
 N,0,-2.5763416494,-1.1193524798,0.0017377876
 H,0,-3.3164148036,-1.8133930314,-0.0709913901
 N,0,-4.0255379203,0.6694996393,-0.1071624141
 H,0,-4.1647126944,1.6719626006,-0.0879229601
 H,0,-4.8338822333,0.0670840333,-0.2100547082
 N,0,1.2717085771,1.5451324759,0.0103304136
 H,0,1.1117711568,2.108985224,-0.8280563137
 H,0,1.1179039833,2.1383320829,0.8292061922
 N,0,2.5966159724,1.1055643372,0.0178773173
 C,0,2.8628474463,-0.1837810027,-0.0063022772
 N,0,1.7305297694,-1.0471997156,-0.0531623101
 N,0,4.0640442282,-0.6711043467,0.0144828294
 H,0,4.2096054443,-1.6728189611,-0.0009340919
 H,0,4.8729122803,-0.0621531201,0.0556257545
 H,0,1.7865653206,-1.7117737926,0.7265948338
 H,0,3.3344240899,1.8052922338,0.0469591219
 H,0,1.7770616074,-1.6107974422,-0.9093804986
 H,0,-1.7595135344,1.5241592042,1.0202352829
 O,0,0.0711964691,0.0530452138,1.9582193365
 H,0,-0.6405258651,-0.2878819905,2.5197434378
 H,0,0.898963179,-0.0156403279,2.4560685916
 O,0,-0.0470659792,0.0907924797,-1.8828407801
 H,0,-0.8242537721,-0.1960821477,-2.3858805281
 H,0,0.7311220084,-0.1119846397,-2.4214065803
```

{5} $[Fe(AGH)(H_2O)_4]^{4+}$ hs

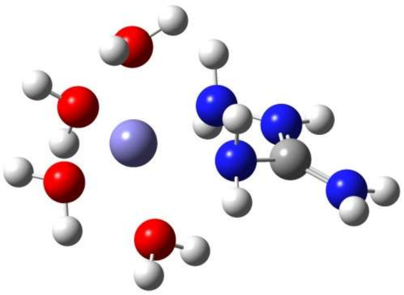

```
Charge =  4 Multiplicity = 6
 Fe,0,0.1904932031,-0.1422467883,-0.008365483
 N,0,-1.3098129268,1.4985374787,-0.3214160716
 H,0,-1.0927464603,2.233548309,0.3515205545
 H,0,-1.1724976828,1.8790319701,-1.2583652542
 N,0,-2.6394527011,1.1256321205,-0.1611505572
 C,0,-2.9442201047,-0.1373773249,0.0868133963
 N,0,-1.9308480666,-1.0701237975,-0.1424105018
 N,0,-4.1194204024,-0.4972974151,0.5204301201
 H,0,-4.3558009132,-1.4779192872,0.5859605874
 H,0,-4.8289785988,0.1910654724,0.7369556014
 H,0,-1.942662225,-1.3494164457,-1.1268741695
 H,0,-3.3360916821,1.8568642149,-0.0563469211
 O,0,1.4471358075,1.5303502461,-0.0687629501
 H,0,2.2671846697,1.3259012024,-0.5384972849
 O,0,1.4044584997,-1.8362720185,0.2959073586
 H,0,2.3400926037,-1.5991932137,0.2425789166
 H,0,1.7071936263,1.8035657503,0.8214620642
 H,0,1.2717085996,-2.1747792791,1.1918586454
 H,0,-2.0566504805,-1.9055835415,0.426157889
 O,0,-0.3152647919,0.0590453677,2.0608341594
 H,0,-0.2904833126,-0.7967997849,2.5081625063
 O,0,0.2920916072,-0.3359675211,-2.1033455347
 H,0,0.578553182,-1.2319683478,-2.3281256637
 H,0,-1.0874282853,0.5241646048,2.407149369
 H,0,-0.4803781648,-0.1558489717,-2.6561807764
```

{6} $[Fe(AGH)(H_2O)_4]^{4+}$ ls

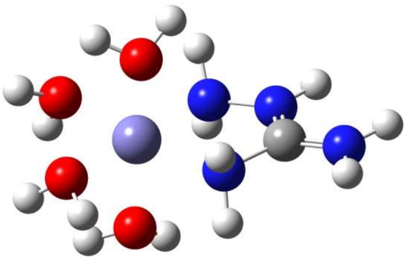

```
Charge =  4 Multiplicity = 2
 Fe,0,-0.0776748273,-0.0611423879,0.0706628739
 N,0,-1.339455201,1.5062878305,0.0135804438
 H,0,-1.2203632636,2.0968597307,0.8401983346
 H,0,-1.117811314,2.0729629206,-0.8085848886
 N,0,-2.6701240169,1.0964512516,-0.0697404926
 C,0,-2.9545407036,-0.1874768902,-0.0241617497
 N,0,-1.8472677298,-1.045375461,0.1915628655
 N,0,-4.1506886639,-0.6703276779,-0.1584731194
 H,0,-4.302227523,-1.669065961,-0.0958103017
 H,0,-4.9439956895,-0.0623915503,-0.3243816311
 H,0,-1.8684701937,-1.8163976863,-0.482186157
 H,0,-3.3871517072,1.8061353905,-0.2000027334
 O,0,1.4940787059,1.0982241152,-0.2086201588
 H,0,2.2527751068,0.6358122559,-0.5922394867
 O,0,1.1423789245,-1.6114101676,0.118528436
 H,0,1.9147598174,-1.4119963354,0.6682306228
 H,0,1.8053344211,1.5055834902,0.6125344415
 H,0,0.7661547971,-2.4248303649,0.4849405025
 H,0,-1.929585738,-1.4670978334,1.1234030045
 O,0,0.0230874224,-0.0064475091,2.0167272515
 H,0,0.9379422361,0.0463834809,2.3293276498
 O,0,-0.2604160555,-0.4437549685,-1.8691256216
 H,0,0.6146600333,-0.460440744,-2.2839776422
 H,0,-0.4847103102,0.6415383761,2.5254247452
 H,0,-0.8005105272,0.1488286949,-2.4118601884
```

{7} $[Fe(AGH)(H_2O)_5]^{4+}$ hs

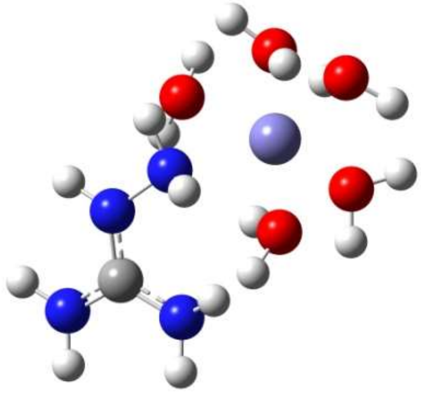

```
Charge =  4 Multiplicity = 6
 N,0,-0.5525099209,1.3854528962,0.0347201634
 H,0,-0.7171833608,1.7804269594,0.9618951895
 H,0,-0.1881770985,2.145296584,-0.5345723248
 N,0,-1.7633092392,1.0121717357,-0.5443064336
 C,0,-2.4918765693,-0.0101269446,-0.0877066953
 N,0,-2.1892713942,-0.5878060278,1.0802309847
 N,0,-3.4988859301,-0.4539737158,-0.822373212
 H,0,-4.1593035069,-1.1073493648,-0.430307866
 H,0,-3.6986691527,-0.0467325074,-1.7228274096
 H,0,-2.8807428734,-1.2100602068,1.4744646433
 H,0,-1.9316410569,1.3554625605,-1.4808845208
 O,0,3.182387248,-1.1068527692,-0.7091814665
 H,0,3.4638008086,-1.7313587516,-0.0280361238
 H,0,2.9540162882,-1.6521657559,-1.4730088806
 H,0,-1.6363253756,-0.078356156,1.7536552546
 O,0,1.5804513033,-0.1317589048,2.1012568742
 H,0,2.43311792,-0.5164925509,2.3433173513
 O,0,0.7716137133,0.1166680678,-2.0825871968
 H,0,1.5387725526,-0.051863234,-2.6457909787
 H,0,0.9185933084,-0.6988266097,2.5180997087
 H,0,0.1248309434,-0.5582435588,-2.3267415577
 Fe,0,1.351189123,-0.1369925601,-0.0307338779
 O,0,2.322713747,1.7473127522,0.0380055523
 H,0,2.1941101656,2.2390618624,0.8588707666
 H,0,2.1634339729,2.3654580814,-0.6857288678
 O,0,0.357107627,-2.0264410198,0.0395491589
 H,0,-0.5412606383,-1.9199703492,0.3868662616
 H,0,0.2542716453,-2.3931396923,-0.8478809468
```

{8} $[Fe(AGH)_2(H_2O)_4]^{5+}$ hs

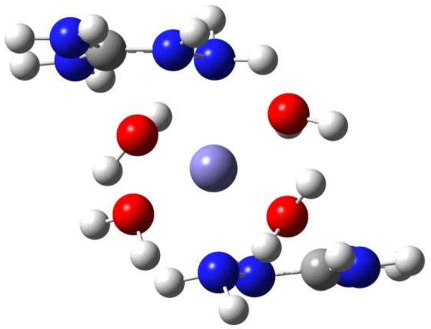

```
Charge =  5 Multiplicity = 6
 N,0,-1.9730555651,1.1404956451,-0.9195297298
 H,0,-1.6697605761,2.1065334362,-0.8284273201
 H,0,-2.0622965915,0.9446037933,-1.9172662915
 N,0,-3.3506197944,-1.2506288662,-0.6309339526
 H,0,-3.8297584864,-2.1136560331,-0.4246244986
 C,0,-3.8616605256,-0.1208635528,-0.1627512439
 N,0,-3.2056769815,1.0407679194,-0.2810946847
 H,0,-3.6998818894,1.901784923,-0.0884428069
 N,0,-5.0603704998,-0.1046671472,0.408273664
 H,0,-5.5722877856,-0.9635558572,0.5284966364
 H,0,-5.4590565992,0.7534623288,0.7541268319
 N,0,1.8940333462,-0.9454277705,1.0063332428
 H,0,2.0902868177,-0.5627037122,1.9331227829
 H,0,1.6319969202,-1.9185901519,1.1516274626
 N,0,3.0612056243,-0.9624577017,0.2437339386
 C,0,3.742569885,0.1446290663,-0.0908177021
 N,0,3.4512573085,1.3227426545,0.4413765525
 N,0,4.7522961583,0.0130566269,-0.9434278025
 H,0,5.3457161896,0.8039289299,-1.1385468869
 H,0,5.0698432126,-0.9017102019,-1.2236986042
 H,0,3.9825538231,2.1252708409,0.1391173559
 H,0,3.231714254,-1.8155540022,-0.2709504062
 H,0,2.559490038,1.499685159,0.8873244745
 H,0,-2.3845913556,-1.3163221533,-0.9221960687
 O,0,0.5444446091,1.6164722206,1.3770731275
 H,0,0.085030316,2.4336241076,1.1404112031
 O,0,-0.33307345,-1.7844241637,-1.1111133662
 H,0,-0.0682905311,-2.5923367512,-0.6522328438
 H,0,0.2945982718,1.4246677414,2.2913055458
 H,0,0.1646069618,-1.766616456,-1.9383270527
 O,0,0.7852031503,1.0224932207,-1.5950995723
 H,0,1.4862689403,0.5093595891,-2.0190466073
 H,0,1.1931642885,1.8570848648,-1.3288056438
 O,0,-0.9996254944,-1.0211528835,1.6265948084
 H,0,-0.6877114199,-0.7970996375,2.5131953991
 H,0,-0.9456992947,-1.9825484829,1.5465327177
 Fe,0,-0.0276332451,0.0211758374,0.070322241
```

{9} $[Fe(AGH)_2]^{5+}$ hs

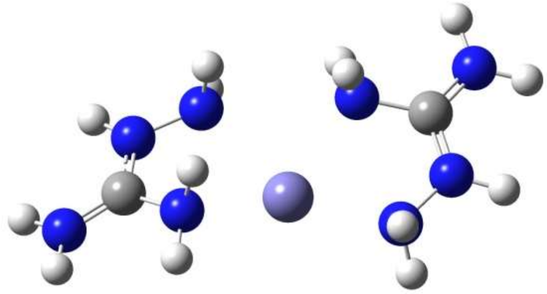

```
Charge =  5 Multiplicity = 6
 Fe,0,-0.0409423382,-0.6152364159,-0.5176831242
 N,0,1.8186053518,-1.8315975234,-0.1948729513
 H,0,1.7301854734,-2.3504188141,0.6805540977
 H,0,1.8634832173,-2.499478491,-0.9655750277
 N,0,1.6186798212,0.7535103241,0.1629033226
 H,0,1.4485484894,0.7518017038,1.1761440022
 C,0,2.900923297,0.2395179565,-0.0853043552
 N,0,2.978046077,-1.0727207364,-0.1882416289
 H,0,3.8697295664,-1.5458755606,-0.3039191656
 N,0,3.9326563354,1.022745082,-0.1994207155
 H,0,3.8405357593,2.0151045126,-0.0274925022
 H,0,4.8510037628,0.6436801928,-0.3958957834
 N,0,-1.2201067594,1.3418992613,-0.0447350952
 H,0,-1.0137924522,1.676325997,0.8984686843
 H,0,-0.925175444,2.0525009537,-0.7143338319
 N,0,-2.5794798085,1.1227352459,-0.2010606607
 C,0,-3.0611947512,-0.0878972673,0.036575487
 N,0,-2.1620696532,-0.9862448066,0.5884420328
 N,0,-4.2834995109,-0.4256402901,-0.2624597654
```

H,0,-4.6441785205,-1.3280836336,0.0156633986
H,0,-4.8991026551,0.223659078,-0.7359988347
H,0,-2.5004159954,-1.9456203838,0.5814608281
H,0,-3.1553283286,1.8681618858,-0.5788198996
H,0,-1.8932130419,-0.721098699,1.5380350121
H,0,1.5388141083,1.7183154282,-0.1610535238

{10} $[Fe(AGH)_2]^{5+}$ ls

Charge = 5 Multiplicity = 2
Fe,0,0.0023972755,-0.0084770256,-0.1305024917
N,0,1.325219098,-1.5353920635,-0.0968728995
H,0,1.0675663757,-2.2038702516,0.6348691675
H,0,1.2617578173,-2.0047168516,-1.0079405476
N,0,1.7436067226,1.0050636243,-0.2939877609
H,0,1.7196515296,1.8489689061,0.2894917976
C,0,2.8683140208,0.2039150127,0.0477973027
N,0,2.6207272194,-1.0867394926,0.1222890565
H,0,3.3454909772,-1.7773144177,0.3077945022
N,0,4.0269979926,0.7370514181,0.2630699814
H,0,4.1494222866,1.7402785376,0.1946276602
H,0,4.8268703983,0.1633532985,0.5070810845
N,0,-1.3131013487,1.5269023069,-0.166287632
H,0,-1.0502813667,2.1723401469,0.585924136
H,0,-1.2360705998,2.0156299158,-1.0637090087
N,0,-2.6210770809,1.1000843822,0.0321185576
C,0,-2.8720897975,-0.1876243016,0.1151509711
N,0,-1.7290932729,-1.0289991345,0.0142762092
N,0,-4.0515564796,-0.6968999987,0.2701464539
H,0,-4.1722048556,-1.7013330723,0.3202828927
H,0,-4.8701546197,-0.1025868532,0.3384072005
H,0,-1.8136973608,-1.6091325128,-0.8313356969
H,0,-3.3513378446,1.8056936272,0.1051308122
H,0,-1.6942299985,-1.6725127796,0.8137695604
H,0,1.8295839119,1.316362579,-1.2702103087

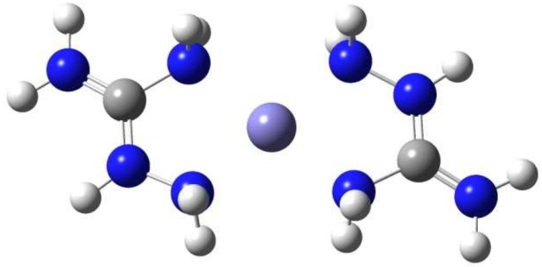

{11} $[Fe(AG_B)_3]^{3+}$ hs

Charge = 3 Multiplicity = 6
N,0,1.9230973503,-0.0699459217,-0.5861920749
H,0,2.7438063602,-0.3505399586,-0.0496216461
H,0,1.7932596278,-0.7674281224,-1.3174054853
N,0,1.1766255006,3.2601160975,-1.4575541258
H,0,0.7153971607,3.9876202593,-0.9185039206
C,0,1.4198170003,2.2486070079,-0.723760078
N,0,2.156038276,1.1816765922,-1.1804643901
H,0,2.3187360416,1.1561420573,-2.17809594
N,0,-1.0552865284,-1.1147022814,-0.7432469679
H,0,-0.4593175684,-1.5022701104,-1.4732484371
H,0,-1.515532987,-1.9027226944,-0.2875904201
N,0,-2.0174419049,-0.2793317951,-1.3357598723
C,0,-2.1887223734,0.9685054278,-0.7870414039
N,0,-2.6270138676,1.9526513928,-1.4646405532
H,0,-2.7870380274,2.7545814153,-0.8613133755
H,0,-2.0622858504,-0.3314587474,-2.3445665305
N,0,-1.8244744491,0.9616282029,0.5972957554
H,0,-1.8898159489,1.8954420917,0.994194663
H,0,-2.4421384688,0.3443934807,1.1274806134
N,0,1.0124547627,2.048295744,0.6323829953
H,0,1.8238883888,2.011992469,1.250614119
H,0,0.4050506616,2.8031454827,0.9401071518

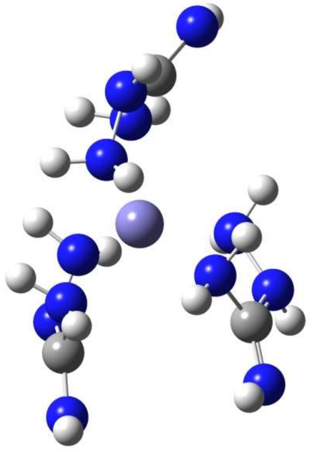

```
Fe,0,0.1439313091,0.0070073633,0.737194291
N,0,0.1259517193,0.1682455963,2.9365526929
H,0,0.9721966014,0.6215788554,3.2781038296
H,0,-0.6622103413,0.7507138395,3.2168956644
N,0,-0.5714071883,-3.2786486619,2.976173636
H,0,-0.3328229691,-4.0030610606,2.3048326019
C,0,0.0267252064,-2.192522536,2.6897144516
N,0,-0.0041716708,-1.1011046992,3.5251305063
H,0,-0.6713665482,-1.1488427136,4.2838177827
N,0,0.8178531663,-1.9235657245,1.5255374078
H,0,1.7934917343,-1.7765659588,1.7894381148
H,0,0.7792490847,-2.7068195594,0.8778462544
```

{12} $[Fe(AG_B)_3]^{3+}$ hs (same orientation)

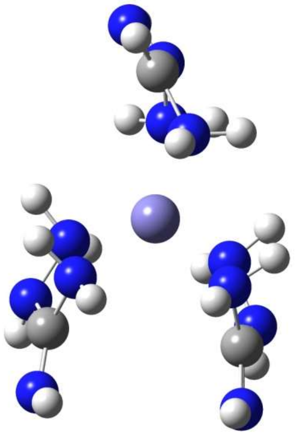

```
Charge =  3 Multiplicity = 6
 N,0,0.5645399829,-1.5435165497,-1.4241962495
 H,0,-0.0428677497,-2.3611342345,-1.4766793888
 H,0,0.6141757014,-1.1543826405,-2.3649852417
 N,0,3.546024656,-1.3605770776,0.4352289468
 H,0,3.6911704157,-1.1334965239,1.4148004115
 C,0,2.3050428707,-1.4992801377,0.1880546069
 N,0,1.8522880284,-1.9440628674,-1.0299393385
 H,0,2.5382698533,-1.9681664884,-1.7724642169
 N,0,0.1201788618,1.5582546154,-1.4967010996
 H,0,0.3597108841,1.167264869,-2.4060572199
 H,0,-0.7142038177,2.128621568,-1.6311791344
 N,0,1.1781789851,2.3730376967,-1.0608769305
 C,0,1.6178941952,2.2053942908,0.2301071826
 N,0,2.798394416,2.5216218711,0.5864332542
 H,0,2.9085939405,2.4158896062,1.5910881439
 H,0,1.8934539696,2.5514441735,-1.7529563527
 N,0,0.5815854313,1.6616934635,1.0522726746
 H,0,0.9286490577,1.4621572128,1.9868103536
 H,0,-0.184065425,2.3329343742,1.1385051901
 N,0,1.2130519591,-1.2605593512,1.0769451314
 H,0,0.7256407463,-2.1343127543,1.282877474
 H,0,1.5381466861,-0.8657186023,1.9553463161
 Fe,0,-0.298158208,-0.0660723504,-0.0227448041
 N,0,-2.1525431027,-0.3331151994,-1.2649926715
 H,0,-2.4023553161,0.5281973583,-1.7482350062
 H,0,-2.010379325,-1.0385158685,-1.986119586
 N,0,-3.7555718959,-1.5792985339,1.6256216532
 H,0,-3.5606005434,-1.4070242799,2.6078573312
 C,0,-3.0576622162,-0.8067526301,0.8925455651
 N,0,-3.2392878944,-0.7301019627,-0.4645409591
 H,0,-3.8397996683,-1.4347741856,-0.8707113137
 N,0,-2.036227111,0.0965784877,1.3253907899
 H,0,-2.3437545942,1.0617899954,1.1921517466
 H,0,-1.8262117839,-0.0355893647,2.3113027314
```

{13} $[Fe(AG_C)_3]^{3+}$ hs

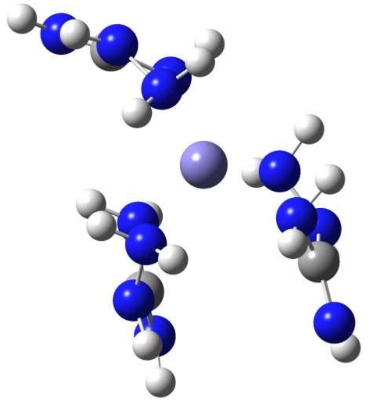

```
Charge =  3 Multiplicity = 6
 N,0,-0.8579399795,-1.4702339153,-1.4035607167
 H,0,-0.5886804331,-1.0795313849,-2.3043649763
 H,0,-0.5876859885,-2.4536554696,-1.41658705
 N,0,-2.2529493983,-1.3684840311,-1.2662872416
 C,0,-2.7285013562,-0.9485300959,-0.0498526534
 N,0,-3.855571568,-0.4189841439,0.2201334647
 N,0,-1.7771963508,-1.190373804,0.9754483465
 H,0,-1.605176338,-2.1894608528,1.090467541
 H,0,-2.1150746853,-0.8216417202,1.8592532556
 H,0,-4.3824646166,-0.2568499875,-0.6356873773
 H,0,-2.7443313282,-1.0448854246,-2.0888839596
 N,0,1.9083238691,-0.7816965439,1.6602988999
 H,0,1.8634080591,-0.1085379025,2.4238100239
 H,0,1.8424662572,-1.7108700447,2.0722498019
 N,0,3.1436813118,-0.6403191886,1.0020182534
 C,0,3.1314546271,-0.4447791919,-0.3573700058
 N,0,4.0135145737,0.1197417177,-1.0822420535
 N,0,1.9601521776,-1.0033564203,-0.944550745
 H,0,1.9783486205,-2.0215498837,-0.8654307157
 H,0,1.9285002821,-0.7672037202,-1.9324784243
 H,0,4.7473630077,0.5169670628,-0.4986879514
 H,0,3.8490339408,-0.1381107408,1.5249986232
 Fe,0,0.2139371689,-0.4271692913,0.2703081784
 N,0,-0.2620912649,1.439583831,1.4123153261
 H,0,-0.8347401536,1.2030884108,2.2211359933
 H,0,0.5852748499,1.8801977031,1.7693893329
 N,0,-0.9657807853,2.3564085106,0.6096096485
 C,0,-0.9614352857,2.1284139445,-0.7455270588
 N,0,-1.7937275085,2.5343234093,-1.6203706568
 N,0,0.1584115178,1.3381452743,-1.1201472398
 H,0,1.0363418603,1.8190253953,-0.9184211408
 H,0,0.1229290955,1.1350890899,-2.1149029515
 H,0,-2.5677120353,3.0214897761,-1.1724566591
 H,0,-1.8239356434,2.7072167223,1.014004888
```

{14} $[Fe(AG_D)_3]^{3+}$ hs

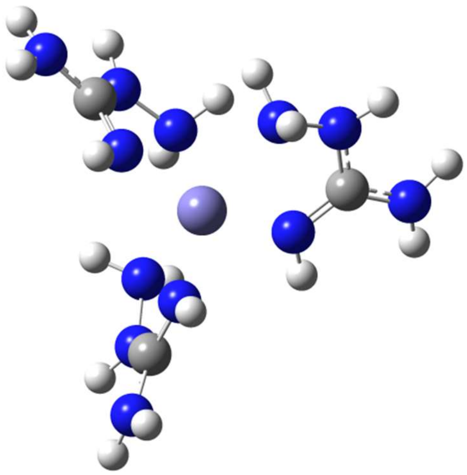

```
Charge =  3 Multiplicity = 6
 Fe,0,-0.4119278836,0.1318198589,0.1077681968
 N,0,-0.2083324727,2.3597979529,-0.2627742679
 H,0,0.3102885949,2.7709240839,0.5122272739
 H,0,0.3100427498,2.5408670973,-1.1184947318
 N,0,-1.470680711,2.9424358697,-0.3597158304
 H,0,-1.5221862665,3.9480946313,-0.2592666952
 C,0,-2.5063840941,2.1848883374,0.0907512452
 N,0,-3.6878002036,2.7867969339,0.2346417775
 H,0,-3.8143237959,3.7468822575,-0.0437269372
 H,0,-4.5023996961,2.2361717509,0.4504643733
 N,0,-2.2751908258,0.9158218787,0.3319498822
 H,0,-3.1006931977,0.3978413443,0.6076020642
 N,0,0.2589778978,-2.0580655522,-0.1529450844
 H,0,-0.4984849907,-2.6654400691,0.1552954701
 H,0,1.0738939448,-2.2623970128,0.4186667512
 N,0,0.5551024788,-2.2978112126,-1.4919616038
 C,0,0.1572909013,-1.3445882885,-2.3757812736
 H,0,0.6030742407,-3.2615552719,-1.796631651
 N,0,0.2308339868,-1.6438433673,-3.6749172367
 H,0,0.6541723498,-2.5056552458,-3.9809679369
 H,0,0.0910755717,-0.9149720067,-4.3550900177
 N,0,-0.2662721922,-0.1994051617,-1.8936357356
```

```
 H,0,-0.4933924395,0.4794430539,-2.6103121674
 N,0,1.9295839896,0.4277789412,0.1359084326
 H,0,2.2403071092,1.2879407848,-0.3075419853
 H,0,2.3661319249,-0.3406139869,-0.3683726072
 N,0,2.351473824,0.4213408229,1.4606792179
 H,0,3.3365886859,0.3126600844,1.6586723664
 C,0,1.4262511359,0.1579512681,2.4111463772
 N,0,1.8615326728,0.0173194653,3.6676034344
 H,0,2.8087041648,0.2652992922,3.9071398555
 H,0,1.185784747,-0.0269755434,4.4125570792
 N,0,0.1670120146,0.0648431896,2.0429272218
 H,0,-0.4683292158,-0.0686341802,2.8201247426
```

{15} [Fe(AG$_D$)$_3$]$^{3+}$ ls

```
Charge =  3 Multiplicity = 2
 Fe,0,0.0017507802,0.3471512863,-0.0277292907
 N,0,-0.0874893983,2.3581915942,-0.1367708406
 H,0,0.3061153542,2.7699696317,0.7082726961
 H,0,0.4303492503,2.7254649052,-0.932740553
 N,0,-1.4318572651,2.7424775705,-0.2678884468
 H,0,-1.6531019377,3.7055748685,-0.0509066863
 C,0,-2.3381142765,1.7679890863,-0.0025824665
 N,0,-3.6197418795,2.1093680856,0.1192295158
 H,0,-3.9201743275,3.0495070339,-0.0846852083
 H,0,-4.3175232509,1.3842144757,0.1626232204
 N,0,-1.8738181941,0.542299991,0.0968229417
 H,0,-2.5632780646,-0.1835584522,0.2485481708
 N,0,-0.0981197746,-1.6641984506,-0.0597591784
 H,0,-0.9005466506,-1.9887382615,0.475539921
 H,0,0.728543855,-2.0992887155,0.3479082942
 N,0,-0.244447597,-2.090797783,-1.3893940279
 C,0,-0.0024155377,-1.1473914106,-2.3346754858
 H,0,-0.0478225242,-3.0641974806,-1.5816913806
 N,0,0.0855103894,-1.5386100418,-3.6062185251
 H,0,-0.1286524419,-2.4886636335,-3.8644600334
 H,0,0.1098452298,-0.8412401478,-4.3326190473
 N,0,0.1153525641,0.0934524399,-1.9146888715
 H,0,0.2283128469,0.7927630466,-2.6376817212
 N,0,2.0626387551,0.3161643562,0.1214067357
 H,0,2.4486226079,1.1973517394,-0.2169535325
 H,0,2.4486846432,-0.42233746,-0.4626203709
 N,0,2.4676641384,0.0946734731,1.4490204132
 H,0,3.412305015,0.3809163444,1.6732614545
 C,0,1.4863949744,0.3060114327,2.3762033536
 N,0,1.8573382408,0.3836675946,3.6642374688
 H,0,2.7770396416,0.0600343093,3.9219047556
 H,0,1.1363665416,0.2847432866,4.361579661
 N,0,0.269450931,0.4118498183,1.9257623922
 H,0,-0.4534556387,0.5121464669,2.6257336722
```

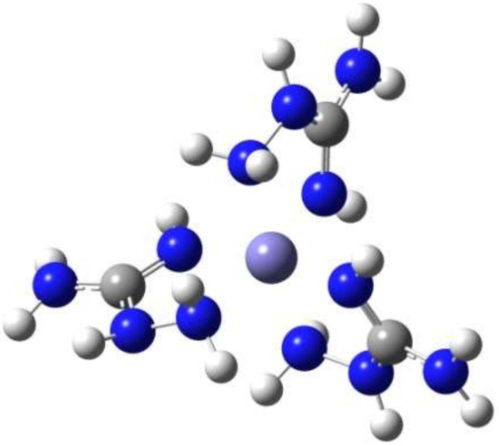

{16} $[Fe(AG_D)_3]^{3+}$ hs (same orientation)

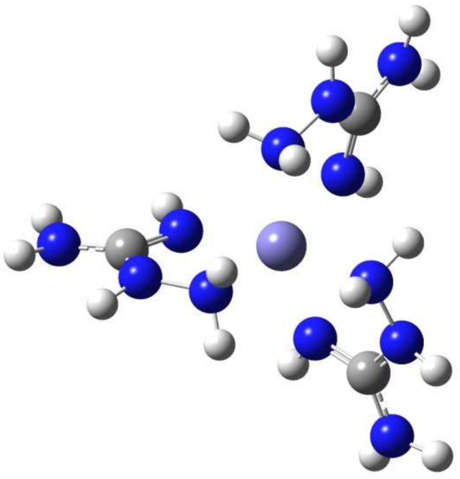

```
Charge =  3 Multiplicity = 6
 Fe,0,-0.0662117452,-0.0289469165,-0.1879240991
 N,0,-0.2705580486,1.7282483338,1.2686198592
 H,0,-1.2321188227,2.0596710569,1.209995999
 H,0,-0.0866220121,1.4798444446,2.2375111961
 N,0,0.6095616658,2.735527059,0.8802425381
 H,0,0.4865892085,3.6556427984,1.281914151
 C,0,1.1851742141,2.6045476462,-0.3405633682
 N,0,1.8932947361,3.6338277742,-0.8075034643
 H,0,2.0730517013,4.4419184368,-0.2331329095
 H,0,2.4249535862,3.5199425072,-1.6547970123
 N,0,1.0174128605,1.4656042225,-0.9775814394
 H,0,1.530414815,1.4071079087,-1.8486011421
 N,0,1.60245049,-0.6356427651,1.2649196556
 H,0,1.2667449043,-0.9002704588,2.1892257114
 H,0,2.2279664702,0.1583737031,1.3802552463
 N,0,2.3073578022,-1.6920832625,0.6974919149
 C,0,1.7909060996,-2.2649399993,-0.4134106452
 H,0,2.9683525547,-2.1950987197,1.2736536988
 N,0,2.360332098,-3.3924080939,-0.8472985659
 H,0,3.2210757498,-3.717551082,-0.4351588637
 H,0,2.1134794417,-3.7410245941,-1.7591639134
 N,0,0.7641193195,-1.6770519598,-0.9913540512
 H,0,0.4387765579,-2.1579228332,-1.8209778619
 N,0,-1.4007606034,-1.0852580019,1.3325668637
 H,0,-1.2774420616,-0.7535023515,2.2862469748
 H,0,-1.1528510213,-2.0732789966,1.3126691255
 N,0,-2.730449789,-0.916281369,0.9598490195
 H,0,-3.427646699,-1.5079614045,1.3916860576
 C,0,-2.9649479489,-0.3637667406,-0.254357875
 N,0,-4.2253965115,-0.3463629087,-0.6902907206
 H,0,-4.9811155135,-0.6480689402,-0.0958668144
 H,0,-4.4490846282,0.1515898433,-1.5359789292
 N,0,-1.9366726842,0.1246648043,-0.915342449
 H,0,-2.1902118563,0.5801942987,-1.7835031971
```

{17} $[Fe(AG_D)_3(H_2O)]^{3+}$ hs

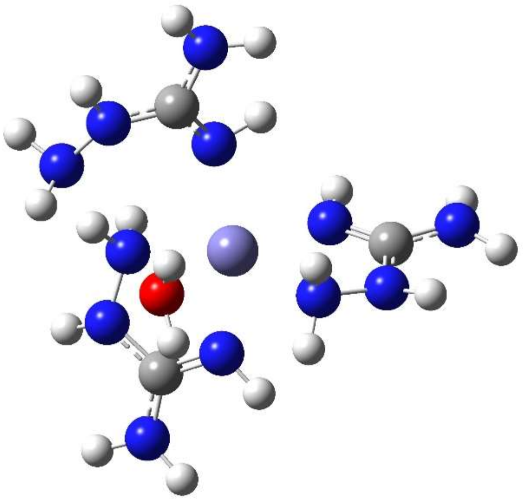

```
Charge =  3 Multiplicity = 6
 Fe,0,0.1507919974,-0.1911082483,-0.0649888756
 N,0,1.6046944293,-0.5085011851,1.6669643496
 H,0,1.7749589536,0.3832969848,2.1278336298
 H,0,1.2005325902,-1.1449822888,2.3483157186
 N,0,2.7934006399,-1.0411953536,1.179249318
 H,0,3.6002164268,-1.0329253033,1.7890232481
 C,0,2.9668502703,-0.9969388488,-0.1651971109
 N,0,4.1851889474,-1.2733476043,-0.6413676782
 H,0,4.8924843195,-1.6450086754,-0.0267084902
 H,0,4.2905131413,-1.4416009806,-1.6289589261
 N,0,1.9274188178,-0.6829309193,-0.9041891502
 H,0,2.1158009013,-0.6981386709,-1.8993628091
 N,0,-1.8219533689,-0.2831444587,-1.0906353847
 H,0,-1.6979039419,-0.3087198588,-2.1007349439
 H,0,-2.2650793757,0.6019272449,-0.8254965428
 N,0,-2.5496061705,-1.3836239567,-0.6541371084
 C,0,-1.8267862557,-2.3563895858,-0.0367098346
 H,0,-3.3629373551,-1.6453176981,-1.1950593708
 N,0,-2.4244365751,-3.5343463294,0.1672154673
 H,0,-3.4035341908,-3.6511242149,-0.0410215356
 H,0,-1.9843788326,-4.2103510932,0.7696722707
 N,0,-0.5979378745,-2.0576859429,0.3147561698
 H,0,-0.1189673081,-2.8027170898,0.805939439
```

```
 N,0,-2.3743603311,2.4771944471,-0.2067760503
 H,0,-2.8671120096,2.3193809899,0.6661057642
 H,0,-3.0004233941,2.9817694843,-0.826981996
 N,0,-1.2710020054,3.2813828561,0.0552062432
 H,0,-1.4485800968,4.2430310133,0.3123893443
 C,0,-0.0209655604,2.9853683228,-0.360078009
 N,0,0.8254830381,4.0335597103,-0.4183354581
 H,0,0.5215038025,4.9474114769,-0.1206686283
 H,0,1.8157356167,3.8511304047,-0.401112991
 N,0,0.375114697,1.7681951985,-0.6959574416
 H,0,1.30701478,1.8227052224,-1.0921899613
 O,0,-1.1316435824,0.4786058652,1.7780988097
 H,0,-1.1325733303,-0.1701284867,2.4913341455
 H,0,-0.7866565499,1.285842782,2.1769029791
```

{18} $[Fe(AG_D)_3(H_2O)_2]^{3+}$ hs

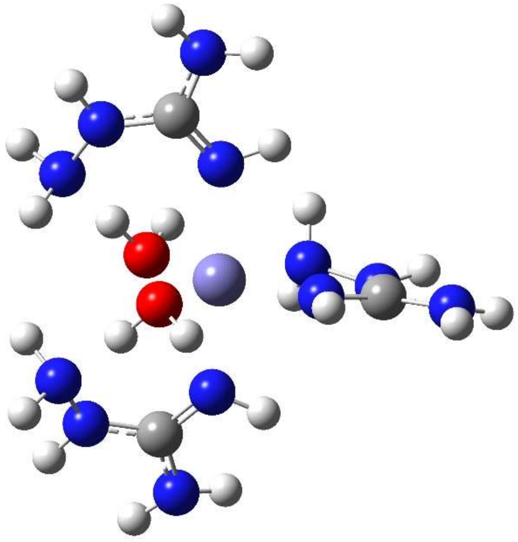

```
Charge =  3 Multiplicity = 6
 Fe,0,0.6407533253,0.2338778002,0.0692653879
 N,0,2.7164605426,0.3605595476,-0.8745215964
 H,0,2.7714966005,-0.2880662606,-1.6547733806
 H,0,2.8681647971,1.301613859,-1.2326973432
 N,0,3.6919943335,0.0376102908,0.0642088295
 H,0,4.6530941076,0.2671664732,-0.1519393286
 C,0,3.2840061036,0.0024522158,1.3592399658
 N,0,4.22396749,-0.1049248896,2.3024289188
 H,0,5.1869881308,-0.2628066075,2.05078807
 H,0,3.9457683868,-0.2782434686,3.2544279874
 N,0,1.9948989319,0.0676859744,1.589547183
 H,0,1.7461496126,-0.0133425072,2.5679845078
 N,0,-2.127473486,-1.5886627078,-0.6442887242
 H,0,-2.4974835778,-0.8982302974,-1.2912990347
 H,0,-2.9152744036,-2.1146280981,-0.27804967
 N,0,-1.3070118731,-2.4659702498,-1.344625721
 C,0,-0.0016583209,-2.65470531,-1.0544847871
 H,0,-1.7527222571,-3.1286507906,-1.963202972
 N,0,0.5452482861,-3.778904072,-1.575477383
 H,0,0.0110971492,-4.3158498284,-2.2415594821
 H,0,1.5430471844,-3.7779537139,-1.7160854571
 N,0,0.6913793236,-1.8294939084,-0.2996695672
 H,0,1.6135623068,-2.2119152574,-0.1203676871
 N,0,-1.970822267,2.0854965832,-1.0288227517
 H,0,-2.6441289506,1.4628271508,-0.5916677679
 H,0,-2.432864206,2.5317009809,-1.8154605832
 N,0,-1.6044589646,3.0720826699,-0.1203380442
 H,0,-2.2721936732,3.8055967285,0.0725601104
 C,0,-0.3339623487,3.2416589182,0.3062756898
 N,0,-0.0764916347,4.4338123995,0.8942565664
 H,0,-0.8441448519,5.0556336625,1.0953847065
 H,0,0.6984822646,4.4723798226,1.5372330776
 N,0,0.5988167956,2.3272776694,0.1532109899
 H,0,1.4873034373,2.6785574332,0.4932559472
 O,0,-1.038070072,-0.0076159694,1.4028124177
 H,0,-1.5352520553,-0.6418457396,0.8313111981
 H,0,-0.8251854145,-0.4932564305,2.2081839595
 O,0,0.0776494276,0.4544763914,-2.0259183174
 H,0,0.694615832,0.9299744106,-2.5931083377
 H,0,-0.6587360126,1.0911421245,-1.8579155768
```

{19} $[Fe(AG_A)_2(H_2O)_2]^{3+}$ ls

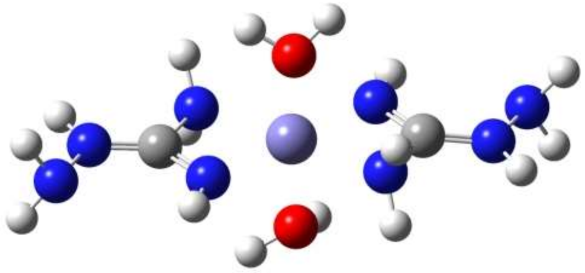

```
Charge =  3 Multiplicity = 2
 N,0,-4.5471385338,-0.6560255755,0.0061817291
 H,0,-5.1550629771,-0.615729857,-0.8051582528
 H,0,-5.1274784556,-0.5943338933,0.8361887481
 N,0,-3.7048149619,0.4477616159,-0.0199889713
 C,0,-2.3995108943,0.2660563441,-0.0535742207
 N,0,-1.6948914293,-0.8178258203,-0.0830994463
 N,0,-1.494767507,1.3734617652,-0.040373921
 H,0,-1.6345809123,1.9838424326,0.7636153989
 H,0,-1.5501785772,1.9282667146,-0.8937943517
 H,0,-2.1384812178,-1.7280470275,-0.0668143721
 H,0,-4.0725404956,1.3930460075,0.0028362887
 N,0,4.6360827125,0.6778322637,-0.1177995813
 H,0,5.165177666,0.6149911899,-0.9815096377
 H,0,5.2915635211,0.6191421274,0.6545174361
 N,0,3.7797578627,-0.4133541573,-0.0513002253
 C,0,2.4814586167,-0.2127803041,0.0559867503
 N,0,1.7960998239,0.8822988581,0.1304973413
 N,0,1.5624353701,-1.3075532718,0.0814881415
 H,0,1.6329404362,-1.8524822337,0.9395250777
 H,0,1.6647050105,-1.9237920574,-0.7229855247
 H,0,2.2542528204,1.7846583549,0.0927560467
 H,0,4.1313726357,-1.3640165018,-0.0951172186
 O,0,-0.0081717715,-0.092607671,2.0191684948
 H,0,0.7286252127,0.3499926452,2.4622205698
 H,0,-0.8047991758,0.191856304,2.4866991916
 O,0,0.2042239326,0.0433128907,-1.9625814125
 H,0,0.7318530723,0.7793263197,-2.3005422562
 H,0,-0.6247021771,0.0655982365,-2.4595039098
 Fe,0,0.0437645329,0.0399295304,0.0379511174
```

{20} $[Fe(AG_A)_2(H_2O)_2]^{3+}$ ls (mirror image)

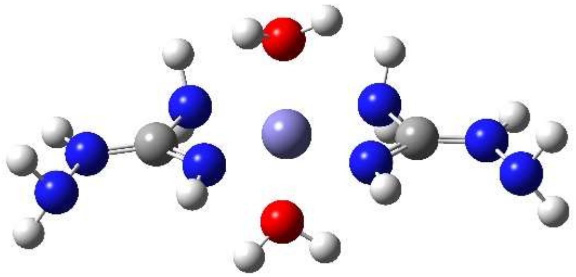

```
Charge =  3 Multiplicity = 2
 N,0,4.404135536,-0.4070606968,-1.2155986261
 H,0,5.0027682168,-1.1873112133,-0.9645761992
 H,0,4.9933500978,0.3321667034,-1.5842444911
 N,0,3.7888635158,0.0617345324,-0.0623415182
 C,0,2.4741394054,0.0312866385,0.0289980393
 N,0,1.5805923835,-0.3801957136,-0.8103885182
 N,0,1.7960432607,0.5044202637,1.1953258819
 H,0,1.9852389617,1.4883054846,1.3812650165
 H,0,2.0297341193,-0.040121467,2.0244933816
 H,0,1.8294960144,-0.7505515501,-1.7194016504
 H,0,4.3294942171,0.409207215,0.7226640305
 N,0,-4.3840163183,-0.3645167971,-1.2255216669
 H,0,-4.9339317846,0.4084841643,-1.5853538083
 H,0,-5.0174145713,-1.1281155103,-1.0128728661
 N,0,-3.7727497982,0.0430841713,-0.047153132
 C,0,-2.4589583371,-0.0347011863,0.0600613766
 N,0,-1.5719608992,-0.4560445316,-0.7783844059
 N,0,-1.7811002346,0.4079130373,1.2391110759
 H,0,-1.9699630497,-0.1940165438,2.0390535028
 H,0,-2.0070359261,1.3708462809,1.4817164789
 H,0,-1.8306460861,-0.7795565583,-1.7024051558
 H,0,-4.3128755053,0.3917109696,0.7370281582
 O,0,0.0136245055,-1.7111082089,1.1720057247
 H,0,-0.6669976004,-2.3298552355,0.8738426691
```

```
 H,0,0.8357088849,-2.2198562145,1.1942505964
 O,0,-0.1172472916,1.8927394394,-0.4353818786
 H,0,-0.7695302725,2.0221355218,-1.136868645
 H,0,0.6788743839,2.3546528882,-0.7306671352
 Fe,0,0.0148143494,0.0273667092,0.2327526499
```

{21} $[Fe(AG_A)_2(H_2O)_4]^{3+}$ ls

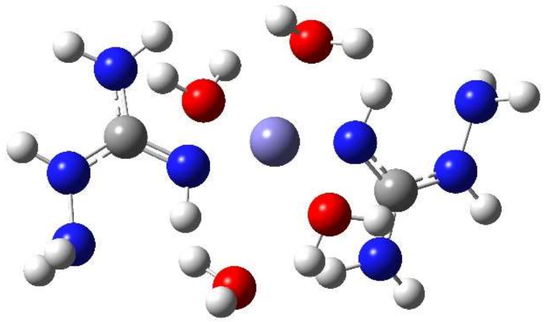

```
Charge =  3 Multiplicity = 6
 N,0,-4.4665522978,-2.4411502494,0.0700532249
 H,0,-5.0759921655,-2.5384466139,-0.7340156554
 H,0,-4.9053875581,-2.8952783989,0.8634952105
 N,0,-4.2819031011,-1.097753178,0.3492267079
 C,0,-3.0399084608,-0.5718988496,0.3656441312
 N,0,-1.9879157386,-1.3168844951,0.0610921776
 N,0,-2.9082163466,0.7106055891,0.7162834911
 H,0,-3.7318526466,1.2780607031,0.8364750321
 H,0,-2.0231954554,1.1699949414,0.5225827432
 H,0,-2.3043106753,-2.2541953291,-0.1812522323
 H,0,-5.0732012119,-0.5096860855,0.5758475457
 N,0,3.966268593,1.3437855726,0.3582118737
 H,0,4.0648207183,2.135076129,-0.2684321023
 H,0,4.7312747548,1.3656638842,1.0231556916
 N,0,4.0300017875,0.1693322144,-0.3735364835
 C,0,2.9970090783,-0.6912728144,-0.3598237503
 N,0,1.9438606675,-0.5107419398,0.4076500074
 N,0,3.0623701875,-1.7511167457,-1.2088512091
 H,0,3.9474167377,-1.9179063625,-1.6652145898
 H,0,2.6083381033,-2.5918436822,-0.8803593559
 H,0,2.0742538762,0.3276014872,0.9693403262
 H,0,4.8506400565,-0.0433688702,-0.926783693
 Fe,0,0.0046886246,-1.1713591431,0.2343682754
 O,0,0.2653389449,-1.1528855854,-1.9016186782
 H,0,1.1627811485,-1.4102217457,-2.1646851524
 H,0,-0.327925539,-1.7666036953,-2.3508124509
 O,0,-0.1675194245,-0.5021400912,2.3043115047
 H,0,-1.0492773632,-0.6182940525,2.6774754296
 H,0,0.0315157561,0.4361889569,2.4105912911
 O,0,0.0239095822,-3.2798312161,0.3928416894
 H,0,0.9250934767,-3.6213011492,0.4582373777
 H,0,-0.4296525733,-3.599282473,1.1836186553
 O,0,-0.3387860867,1.877541904,-0.0068105945
 H,0,0.3661635337,2.0001146412,0.636070847
 H,0,0.1165509871,1.7115996929,-0.8375488454
```

{22} $[Fe(AG_B)_2(H_2O)_2]^{3+}$ hs

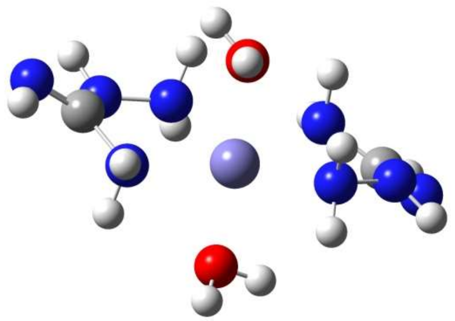

```
Charge =  3 Multiplicity = 6
 N,0,-1.6848315918,-1.3364497849,0.9361169654
 H,0,-1.4988433793,-2.3000304015,0.6669435795
 H,0,-1.7227230809,-1.29803262,1.9537362548
 N,0,-3.9157490571,1.0063325183,-0.4024575994
 H,0,-3.7871003731,1.7166232362,-1.1179491275
 C,0,-2.9161361929,0.218397607,-0.3707837075
 N,0,-2.9158136848,-0.9217309684,0.4005408479
 H,0,-3.6945396579,-0.998152452,1.0416683635
 N,0,1.1073976827,1.1344673218,-1.2499116712
 H,0,0.9186571304,0.7384726041,-2.1704406877
 H,0,0.7440219322,2.085764865,-1.2378464242
 N,0,2.4889679848,1.1665929692,-1.0078687899
 C,0,3.0070000865,0.2192684286,-0.1589925878
 N,0,4.1749386561,-0.2604960925,-0.3144937671
```

```
H,0,4.4320872008,-0.8365287991,0.482502262
H,0,3.051968172,1.3509459025,-1.8286438681
N,0,2.0955212061,-0.077501646,0.9118316345
H,0,2.3452805355,-0.963774097,1.3448018668
H,0,2.1793942112,0.6469361469,1.6275619812
N,0,-1.7009030718,0.3436911682,-1.1057157091
H,0,-1.5895084768,-0.4270403284,-1.7637282188
H,0,-1.6807906634,1.2201999462,-1.6193347916
O,0,0.37430982,-1.6442590192,-1.0257511401
H,0,1.055745729,-1.5663606141,-1.7060638764
H,0,0.5683278657,-2.4745871923,-0.5711708586
O,0,-0.2680044017,1.6704003872,1.6989654835
H,0,-0.1024233567,1.3798593173,2.6060170502
H,0,-1.2082394615,1.8940871313,1.6709269186
Fe,0,0.0011084869,-0.0090799443,0.3353197572
```

{23} $[Fe(AG_B)_2(H_2O)_2]^{3+}$ ls

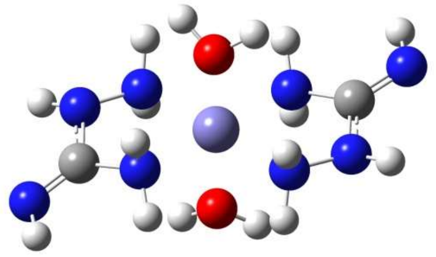

```
Charge =  3 Multiplicity = 2
N,0,-1.2548119923,1.5888104016,0.0308853047
H,0,-0.9920866125,2.2840093465,0.7284213767
H,0,-1.2078363309,2.0447784508,-0.8828846252
N,0,-3.9659239763,-0.600091646,-0.3097380896
H,0,-3.985455461,-1.6168474132,-0.300250684
C,0,-2.8316884507,-0.169845715,0.0636451517
N,0,-2.5696906165,1.1573095782,0.2908368156
H,0,-3.2859664668,1.8037411141,-0.0182347977
N,0,1.2867030333,-1.5102830462,0.0547700706
H,0,1.2700543998,-1.9602197688,0.97189502
H,0,0.9844781986,-2.1991100666,-0.6329136397
N,0,2.5913875185,-1.0953440196,-0.2610991865
C,0,2.8902075033,0.2292772337,-0.0906623363
N,0,4.0648013033,0.6513050041,0.141770408
H,0,4.0924707788,1.6673383338,0.1185099991
H,0,3.3183967272,-1.75530384,-0.0126284202
N,0,1.7075517214,1.0561205924,-0.2884337643
H,0,1.784434905,1.8943759296,0.2878507336
H,0,1.6958753258,1.3709376439,-1.2613890641
N,0,-1.6669219559,-0.9748100457,0.3760747765
H,0,-1.6847013999,-1.2026838609,1.3726711095
H,0,-1.7149665105,-1.8548382417,-0.1360967851
O,0,0.2362229922,0.2421214429,1.9700196769
H,0,1.1088856044,0.1784218956,2.3835203153
H,0,-0.2361077162,0.9449077746,2.4375274334
O,0,-0.2344275593,-0.2945668739,-1.8612093659
H,0,0.5190839443,-0.3746838506,-2.4626196104
H,0,-0.9357830468,0.1489090438,-2.3576899013
Fe,0,0.0232373298,0.0412444127,0.0435966785
```

{24} $[Fe(AG_B)_2(H_2O)_2]^{3+}$ hs (mirror image)

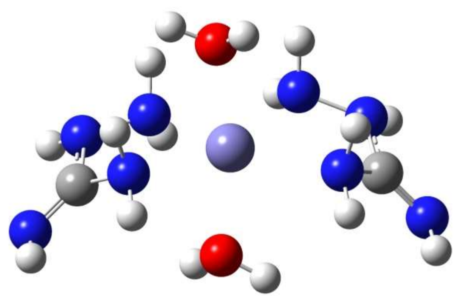

```
Charge =  3 Multiplicity = 6
N,0,-1.3444010435,1.4805093854,-0.1155476384
H,0,-1.2607488998,2.2225578196,0.5762033561
H,0,-1.0404000305,1.8555761354,-1.014166235
N,0,-3.8707449302,-0.7938314097,-0.9011609071
H,0,-3.9669463062,-1.7836469545,-0.690227166
C,0,-2.9236520023,-0.2849942798,-0.2207321402
N,0,-2.6863738434,1.0706018413,-0.2032392638
H,0,-3.2330968439,1.595510392,-0.8744070173
N,0,1.5491138657,1.499723759,0.2448698381
```

H,0,1.2404450716,2.2033344826,-0.4222557973
H,0,1.5971273143,1.9449327926,1.1589894737
N,0,2.8255528301,1.0512780119,-0.1332983141
C,0,2.9900974153,-0.2944001864,-0.3580221285
N,0,3.8412117142,-0.7302398835,-1.1994714703
H,0,3.9107694152,-1.7422758648,-1.1433241403
H,0,3.3021783521,1.6536991795,-0.7912950332
N,0,2.1338775667,-1.0704200683,0.4763933195
H,0,2.1771350266,-2.0554080414,0.2319051736
H,0,2.3920964533,-0.9594886382,1.4574017109
N,0,-2.0072171449,-0.98076728,0.6310125325
H,0,-2.2681236612,-0.8343275029,1.6060369881
H,0,-2.0376280923,-1.9792972106,0.4411729614
O,0,-0.159324367,0.6774248025,2.3596600993
H,0,0.3099759924,0.1461275859,3.0177408377
H,0,-1.035411933,0.8397293297,2.7341541214
O,0,-0.1913040709,-1.1137199641,-1.522645472
H,0,0.6412216587,-1.3780141598,-1.936439106
H,0,-0.6395633307,-0.5783379019,-2.1917933485
Fe,0,0.0487665435,-0.1895386216,0.4257855258

{25} $[Fe(AG_B)_2(H_2O)_2]^{3+}$ ls (mirror image)

Charge = 3 Multiplicity = 2
N,0,-1.509751072,1.3450307894,-0.6066871681
H,0,-1.4473848536,2.2747380517,-0.1935941266
H,0,-1.4314552409,1.4555430982,-1.6196472186
N,0,-3.7826514342,-1.2920287106,-0.1632399104
H,0,-3.637862537,-2.2127456872,0.2423495491
C,0,-2.7723342119,-0.5494528146,0.033245491
N,0,-2.7551465571,0.7856913196,-0.2720455484
H,0,-3.528985201,1.1212243939,-0.8325824034
N,0,1.5135682159,1.3445078707,-0.5827618273
H,0,1.454815859,1.4203054398,-1.6001440105
H,0,1.4278981636,2.2826751224,-0.1935968145
N,0,2.7537366927,0.8054503997,-0.2004431851
C,0,2.7817153503,-0.5284032845,0.1128598255
N,0,3.8137553394,-1.2516385483,-0.0371296929
H,0,3.6674937269,-2.175106399,0.3617339767
H,0,3.5415917722,1.1496850844,-0.7357567757
N,0,1.5132177974,-0.9490865665,0.6866955961
H,0,1.3678490307,-1.9387258144,0.486897008
H,0,1.5807804686,-0.8628588947,1.7033502533
N,0,-1.5251025469,-0.9446628188,0.6631906183
H,0,-1.6198342106,-0.8257025401,1.6742168618
H,0,-1.3594495892,-1.9361633128,0.4926601611
O,0,-0.0155159005,1.0620754808,1.8023902515
H,0,0.7982997057,1.4420636656,2.1628004695
H,0,-0.7340369666,1.6640978351,2.0423153901
O,0,0.0003465765,-0.8468796846,-1.5930403276
H,0,0.8165802006,-1.0643239558,-2.0657821068
H,0,-0.682230115,-0.7096268463,-2.2645582758
Fe,0,0.0008035471,0.1902853368,0.0564349297

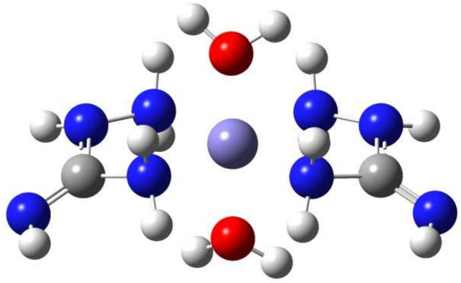

{26} $[Fe(AG_B)_2(H_2O)_4]^{3+}$ hs

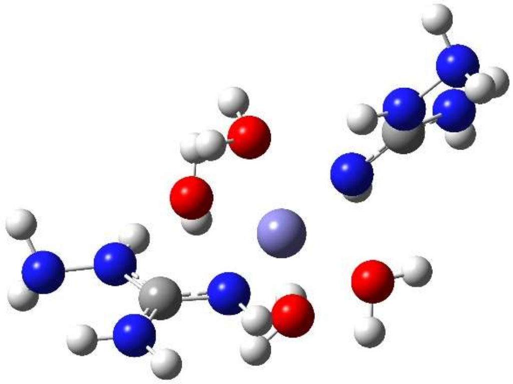

```
Charge =  3 Multiplicity = 6
 N,0,2.6303233743,-3.0946811502,-1.2421578343
 H,0,2.0497450681,-3.4282607282,-2.003435215
 H,0,2.6352919244,-3.8046811514,-0.5177495697
 N,0,2.2445691557,0.3099900395,-0.192137576
 H,0,2.9013791162,1.082328683,-0.1666491056
 C,0,2.8078630654,-0.7982200957,-0.6648315904
 N,0,2.0799621237,-1.927046576,-0.735052797
 H,0,1.1596191404,-1.9290881963,-0.3142119457
 N,0,-3.0019506231,2.07102886,3.2022904056
 H,0,-2.7876965539,3.024365903,3.4764307814
 H,0,-2.8646036261,1.4763026245,4.0115139161
 N,0,-2.1230037225,1.6789737049,2.2013665561
 C,0,-2.5790282585,1.396215196,0.9693956371
 N,0,-1.7090188702,1.1139128983,-0.001224083
 H,0,-2.1796762201,0.8948095984,-0.8708642522
 H,0,-1.1188349683,1.7396682723,2.3438789005
 N,0,-3.8971622448,1.4011198225,0.770374898
 H,0,-4.271813323,1.2548782984,-0.1512811949
 H,0,-4.4979264279,1.6941638971,1.5250238546
 N,0,4.0838769307,-0.8490847478,-1.0563012098
 H,0,4.4178416421,-1.7018467902,-1.4786048051
 H,0,4.5976812472,0.0062513969,-1.1840132439
 O,0,0.8010736479,2.876109981,-0.5793829821
 H,0,0.1094908372,3.5088212938,-0.3518593279
 H,0,1.6045907391,3.224962233,-0.175739275
 O,0,0.0117549862,-0.9991257428,1.2971676112
 H,0,-0.8664504667,-1.0635148172,1.6911181813
 H,0,0.625910157,-1.0002448499,2.0415348768
 Fe,0,0.2937403139,0.841346717,0.0789558831
 O,0,0.8378842418,1.7819774883,2.1825256031
 H,0,1.4277376944,1.1811984992,2.6518897415
 H,0,1.3331145481,2.6024216625,2.081322251
 O,0,-0.0489378956,0.330695079,-2.0770754922
 H,0,0.7772418033,0.2300949646,-2.563371307
 H,0,-0.5170959767,1.0393861623,-2.5344583607
```

{27} $[Fe(AG_C)_2(H_2O)_2]^{3+}$ hs

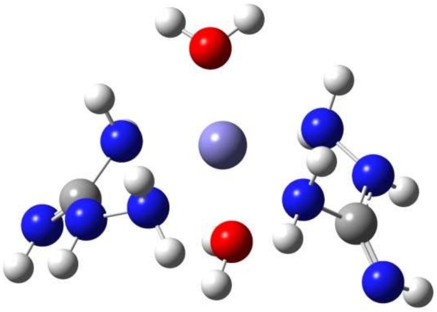

```
Charge =  3 Multiplicity = 6
 N,0,-1.3163675815,-0.4277457518,1.5194464956
 H,0,-0.8407544692,-1.2704740668,1.8373828511
 H,0,-1.4600231505,0.1710267202,2.330995003
 N,0,-2.558292882,-0.7758908569,0.9662957947
 C,0,-2.764458938,-0.5432820309,-0.3709665799
 N,0,-3.5441867504,-1.167728948,-1.1609331526
 N,0,-2.0265826866,0.5872200331,-0.8271961359
 H,0,-2.4657893806,1.4437853772,-0.4864223953
 H,0,-2.0334391838,0.6145173561,-1.8426617171
 H,0,-3.9535727814,-1.9693280764,-0.6839521217
 H,0,-2.9583928042,-1.6294937182,1.3347750244
 N,0,1.8778183058,0.958651326,-1.0945818356
 H,0,1.6275994958,0.7314965824,-2.0565913997
 H,0,2.1704284127,1.9333591349,-1.0690709917
 N,0,2.9296518986,0.1310410141,-0.6762491024
 C,0,2.6935035853,-0.7113255332,0.3819998319
 N,0,3.2757666122,-1.8091360232,0.6591409237
 N,0,1.6974504778,-0.1846422301,1.2525999784
 H,0,2.0608137161,0.6372577015,1.7385780974
 H,0,1.4653806945,-0.8813530658,1.9546166269
 H,0,3.918043969,-2.0664358669,-0.0879264015
 H,0,3.4900217307,-0.2493845641,-1.4283494006
```

```
 O,0,-0.2477907683,2.6711362613,0.8016731231
 H,0,-1.0643092173,3.1192620902,0.545045357
 H,0,0.4733505522,3.2417045557,0.5036536816
 O,0,0.192888886,-1.3027701159,-0.8207781287
 H,0,-0.0467517959,-2.1109611128,-0.3502145603
 H,0,-0.1530878304,-1.4046569311,-1.7161652434
 Fe,0,0.0115218833,0.6929317195,0.0808783776
```

{28} $[Fe(AG_C)_2(H_2O)_2]^{3+}$ ls

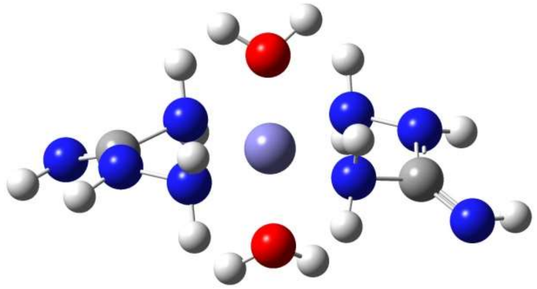

```
Charge =  3 Multiplicity = 2
 N,0,1.3403606588,-1.4816442655,-0.1854354426
 H,0,1.1798543921,-2.0483149069,0.649443691
 H,0,1.17833418,-2.0736075384,-0.9993424979
 N,0,2.6714534434,-1.026634306,-0.2110594988
 C,0,2.8993964726,0.275930767,0.1444147111
 N,0,3.9493487655,0.8095922964,0.6183222909
 N,0,1.7532587298,1.1041378016,-0.1709029875
 H,0,1.8882338823,1.5019773102,-1.1031790776
 H,0,1.7298318844,1.8908484729,0.4766186766
 H,0,4.6535775586,0.0990980805,0.8101147067
 H,0,3.3528878392,-1.7111561461,0.0955014972
 N,0,-1.2708356779,1.5746757404,-0.2129364964
 H,0,-1.0959236506,2.1699661099,0.5987234141
 H,0,-1.1380970036,2.1480567611,-1.0456005975
 N,0,-2.5971512813,1.1051622799,-0.1860593866
 C,0,-2.791330717,-0.2018768562,0.1734121309
 N,0,-3.808766198,-0.7502967966,0.6986292017
 N,0,-1.6512645807,-1.0073511333,-0.203655627
 H,0,-1.7917711257,-1.344896209,-1.1582394038
 H,0,-1.6145167238,-1.8291673788,0.3975948963
 H,0,-4.5108074435,-0.0493457257,0.9300930561
 H,0,-3.2718836673,1.7793426625,0.1547933797
 O,0,0.044625606,-0.0120928439,-2.1461986325
 H,0,0.867222722,-0.021249803,-2.6547809715
 H,0,-0.6019981372,0.49205927,-2.6588273614
 O,0,-0.0032917515,-0.0413420103,1.7640934251
 H,0,0.8138980312,-0.0291348788,2.2822237714
 H,0,-0.665856353,0.4377961188,2.2810122678
 Fe,0,0.0455355454,0.0527175874,-0.1914262353
```

{29} $[Fe(AG_C)_2(H_2O)_2]^{3+}$ hs (mirror image)

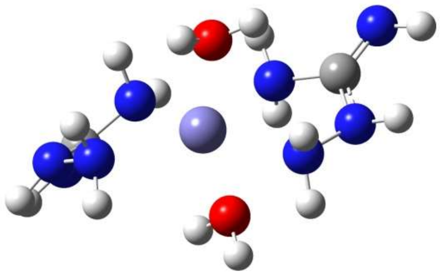

```
Charge =  3 Multiplicity = 6
 N,0,-1.9207448401,1.4629841962,-0.3310680866
 H,0,-2.1882730919,2.0796631026,0.4356065029
 H,0,-1.8242211686,2.0296196069,-1.1701200849
 N,0,-2.918446483,0.4982427659,-0.5386517319
 C,0,-2.703211431,-0.7789178259,-0.0843745592
 N,0,-3.5686748122,-1.6464484602,0.2596363334
 N,0,-1.3105781305,-1.1203682529,-0.0922393676
 H,0,-1.0599150215,-1.4333683409,-1.0311285151
 H,0,-1.1668985918,-1.9081905248,0.5346895216
 H,0,-4.5029542875,-1.2407692068,0.2465691708
 H,0,-3.8648520425,0.8447167615,-0.4407142917
 N,0,2.1612429333,1.3843896989,0.6912106206
 H,0,2.2346265726,2.2528428254,0.1651299637
 H,0,2.2469414747,1.6044895469,1.6805145205
 N,0,3.1700753302,0.4993148107,0.2978363168
 C,0,2.7857833945,-0.7007634745,-0.2433540037
 N,0,3.4291620041,-1.435461485,-1.0615320184
 N,0,1.5126613431,-1.1161172192,0.2477195455
```

H,0,1.5643098502,-1.3106297808,1.2488779778
H,0,1.2409086843,-1.9714843012,-0.2272352431
H,0,4.2815891033,-0.9630676659,-1.3582218509
H,0,3.9849361433,0.9371153479,-0.1120111735
Fe,0,0.0404553807,0.5711670966,0.2375142315
O,0,-0.7676976008,0.5483271984,2.2176832682
H,0,-0.3347430358,1.1969263585,2.7873034821
H,0,-1.7117816576,0.6076168699,2.4125385917
O,0,0.1635282898,0.4766004555,-1.9410941011
H,0,0.958926837,0.0855240846,-2.3248276494
H,0,0.0923698536,1.3438808118,-2.3606283702

{30} $[Fe(AG_C)_2(H_2O)_2]^{3+}$ ls (mirror image)

Charge = 3 Multiplicity = 2
N,0,-1.4689368384,1.502130579,-0.1366435587
H,0,-1.5111149494,2.0612679511,0.7177325038
H,0,-1.3029488686,2.1400968622,-0.9144506582
N,0,-2.6950192621,0.8413008819,-0.344025979
C,0,-2.7569466315,-0.4876247633,-0.0132362089
N,0,-3.7607939016,-1.1864202705,0.3264443578
N,0,-1.4635095503,-1.1190891658,-0.1757423455
H,0,-1.4120256877,-1.5132507387,-1.1173115519
H,0,-1.3984532668,-1.9015682152,0.4737046838
H,0,-4.5885857576,-0.6005784168,0.4228211672
H,0,-3.5113663188,1.4020961168,-0.1300197563
N,0,1.5429240457,1.4880983829,0.2358159168
H,0,1.5685991742,2.1008196882,-0.5812527299
H,0,1.3796307311,2.0703217156,1.0568130141
N,0,2.7737934678,0.8234933632,0.3793748705
C,0,2.8258334272,-0.4958706252,0.0150168871
N,0,3.822836494,-1.1900225318,-0.3534399125
N,0,1.5306571879,-1.1272892968,0.1726378669
H,0,1.4895071042,-1.5497339453,1.1025880491
H,0,1.4622712462,-1.8931250087,-0.4961019879
H,0,4.6530417168,-0.6060342077,-0.4410415079
H,0,3.584336112,1.3914777075,0.1631096609
Fe,0,0.0339575882,0.1761187197,0.0199600735
O,0,-0.2159564399,0.0312017405,1.9541227985
H,0,0.5059287316,0.2368501345,2.5645400107
H,0,-1.0330245691,0.3248832593,2.3808657733
O,0,0.2489558116,0.1455673142,-1.9186470525
H,0,1.1005484551,0.3146471556,-2.3452987239
H,0,-0.4329979015,0.5313118235,-2.4856981508

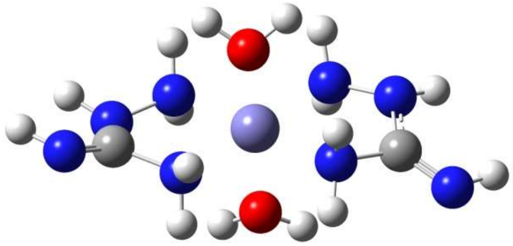

{31} $[Fe(AG_C)_2(H_2O)_4]^{3+}$ hs

Charge = 3 Multiplicity = 6
N,0,-5.1043151213,-1.4147085721,0.1139524726
H,0,-5.8461490019,-0.9471012462,0.6232952053
H,0,-5.2301602369,-2.4154014789,0.2178985634
N,0,-3.8808340915,-1.0481421102,0.6509997346
C,0,-2.9427568072,-0.4473704034,-0.1073882723
N,0,-1.7954966932,-0.0570465999,0.4352094898
N,0,-3.1928765396,-0.2858313684,-1.408185254
H,0,-4.1280659162,-0.4906589127,-1.7251051483
H,0,-2.6168578594,0.3624023247,-1.9250992501
H,0,-1.7911712127,-0.1996677627,1.4393182565
H,0,-3.6794383051,-1.2119564659,1.6286162045
N,0,4.9619715883,2.1896174777,-1.8703655773
H,0,5.6345836416,2.7429070129,-1.3518837836

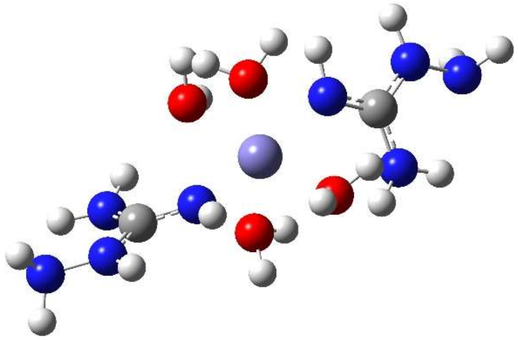

```
H,0,5.0916814422,2.375326128,-2.8591989343
N,0,3.6799872998,2.5597371481,-1.4941513298
C,0,2.7953198447,1.6398530086,-1.0571600939
N,0,1.5604780277,1.9884803891,-0.7268074537
N,0,3.2083196217,0.3695905486,-0.9925728248
H,0,4.1862217417,0.1870704055,-1.1562293712
H,0,2.6514643921,-0.3050091812,-0.4951564945
H,0,1.3983276776,2.9842287429,-0.8283423284
H,0,3.356801432,3.5071579767,-1.6377958291
O,0,-0.7697396612,2.6112956293,0.9477088595
H,0,-1.6133528763,2.9392560702,0.6162976079
H,0,-0.1816641772,3.3742415323,0.925293772
O,0,0.1549388986,-0.9253627802,-1.5742181583
H,0,0.9894644358,-0.9346955008,-2.0578476156
H,0,0.1469786807,-1.7375381969,-1.0537076089
Fe,0,-0.1098068181,0.8184088462,-0.3268554558
O,0,0.9256697448,0.1003418259,1.4907225173
H,0,1.6546707047,0.6938217986,1.7069695248
H,0,0.396454279,0.0527549377,2.2957301381
O,0,-1.1672238334,1.8683290101,-1.9626313918
H,0,-1.3088936659,2.7961102724,-1.7387271316
H,0,-0.6423801158,1.872464224,-2.7721966989
```

{32} $[Fe(AG_D)_2(H_2O)_2]^{3+}$ hs (trans)

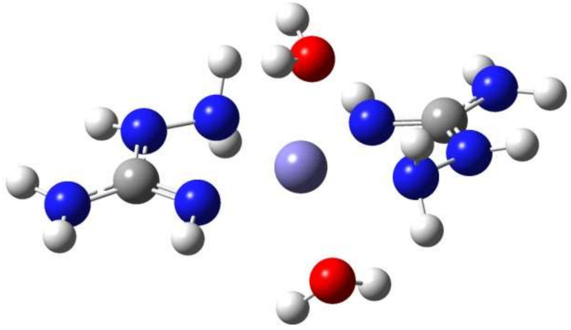

```
Charge =  3 Multiplicity = 6
N,0,1.8448575035,-1.4602641512,0.5341027759
H,0,1.8663355011,-1.6336430827,1.536073953
H,0,1.8757938645,-2.3526470455,0.0483451406
N,0,1.4419797056,0.9454400884,-0.4001020542
H,0,1.3440779043,1.8996566662,-0.7238912706
C,0,2.697910135,0.5810756368,-0.2332676288
N,0,2.9374498333,-0.6895156655,0.1585757203
H,0,3.8573988745,-0.9730730638,0.4677064424
N,0,3.7489290762,1.3787872482,-0.4296306574
H,0,3.6109413168,2.2811750406,-0.8542516618
H,0,4.686738116,1.0131036419,-0.3798208099
N,0,-1.2752116996,1.5894839222,-0.3784313115
H,0,-0.9759856411,2.3218961061,0.2597409204
H,0,-1.0867128932,1.89322023,-1.3320622629
N,0,-2.6296707258,1.339820126,-0.2051672214
C,0,-2.990458105,0.0381586371,-0.1077818148
N,0,-2.0303241018,-0.8501700132,0.0385418948
N,0,-4.290529524,-0.2516416648,-0.1539137676
H,0,-4.5947551262,-1.1940464681,0.0269041163
H,0,-4.9825170238,0.4800786697,-0.1887079205
H,0,-2.3678069406,-1.8010496283,0.1320781905
H,0,-3.288202393,2.0184913339,-0.5639378176
O,0,0.2630844851,-1.5075818795,-1.7654366148
H,0,-0.4629058272,-1.4262355902,-2.3961492688
H,0,1.0537810041,-1.2589671119,-2.26038364
O,0,0.274480564,0.1205739553,2.2848196866
H,0,-0.4074270412,-0.3243595283,2.8019611636
H,0,0.08811117,1.060773915,2.3951237797
Fe,0,-0.0634740115,-0.3402443345,0.0480739488
```

{33} [Fe(AG$_D$)$_2$(H$_2$O)$_2$]$^{3+}$ ls

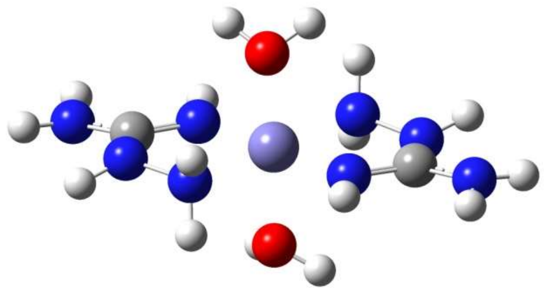

```
Charge =  3 Multiplicity = 2
 N,0,-1.2383000682,-1.5374438353,-0.0053531415
 H,0,-1.1438252835,-2.0128655344,-0.9018441316
 H,0,-1.0297975244,-2.2048912651,0.7345267553
 N,0,-1.6276380515,0.987510345,0.0084415757
 H,0,-1.760354738,1.9906540128,-0.0194215642
 C,0,-2.7249780627,0.2581678782,0.0340594151
 N,0,-2.5551956103,-1.0809408035,0.1538612697
 H,0,-3.296489689,-1.7153756695,-0.113581505
 N,0,-3.9628968182,0.7389475171,-0.0313574465
 H,0,-4.1066852873,1.7352928103,0.0099035869
 H,0,-4.7577513732,0.1311431578,0.0893814305
 N,0,1.2823792432,1.5883162548,0.0640769855
 H,0,1.045700452,2.2547859806,-0.668642819
 H,0,1.2306817073,2.0793160832,0.9566934517
 N,0,2.5912135008,1.1283608007,-0.1488889603
 C,0,2.7581417614,-0.2133679146,-0.0431515121
 N,0,1.6590771669,-0.9368959888,0.0141477435
 N,0,3.9953205759,-0.7011369397,-0.020030492
 H,0,4.1304110264,-1.6972527538,-0.0892162282
 H,0,4.7884650622,-0.0968435062,-0.1673560938
 H,0,1.7876932712,-1.9399730181,0.0575084295
 H,0,3.3438696714,1.7578084325,0.0975816294
 O,0,-0.0069957038,-0.0990958932,2.0279011371
 H,0,0.7281505093,0.3146889037,2.5001110321
 H,0,-0.8100490887,0.2586554424,2.4301188426
 O,0,-0.0340215501,-0.0058339933,-1.9682365915
 H,0,0.826309116,-0.0521091375,-2.4059531183
 H,0,-0.4768655734,0.767746184,-2.3419337162
 Fe,0,0.0176895283,0.0250266396,0.0412785957
```

{34} [Fe(AG$_D$)$_2$(H$_2$O)$_2$]$^{3+}$ hs (mirror image)

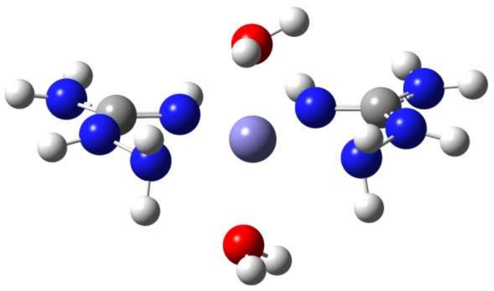

```
Charge =  3 Multiplicity = 6
 N,0,-2.1185518944,1.4889299242,0.03792709
 H,0,-2.2635776165,1.9598702689,0.9280588787
 H,0,-2.2098834216,2.175378417,-0.7063786695
 N,0,-1.3504365709,-0.99077809,0.0915031121
 H,0,-1.1032245104,-1.9714889445,0.1283205855
 C,0,-2.6453296111,-0.7843287919,-0.0104195696
 N,0,-3.0698128797,0.4926288702,-0.1414988133
 H,0,-4.0421077942,0.7246801766,0.0121176346
 N,0,-3.5686419025,-1.7497368114,0.0128953951
 H,0,-3.2680500666,-2.7067101885,-0.0800815949
 H,0,-4.5274183929,-1.5371815838,-0.2151301046
 N,0,2.1148738341,1.5023543352,0.0918554398
 H,0,2.1989705798,2.2175973772,-0.6250189715
 H,0,2.2353983018,1.9420027149,1.0021339889
 N,0,3.0850203307,0.5291138494,-0.1091450268
 C,0,2.6758381041,-0.7594944351,-0.0339929799
 N,0,1.3814120164,-0.989122789,0.0227212503
 N,0,3.6119348292,-1.7104843019,-0.0220171836
 H,0,3.3328448835,-2.6733456284,-0.1179499961
 H,0,4.5801681239,-1.4785737392,-0.1794286237
 H,0,1.1481613937,-1.9738547996,0.0038748809
 H,0,4.0452931098,0.7643751937,0.1049525948
 O,0,-0.2164678191,1.058355835,2.1120636981
 H,0,0.4858156561,0.65294643,2.6365103876
 H,0,-0.1305084741,2.0081682598,2.2637130537
 O,0,-0.0433223022,0.8539173076,-2.167621705
```

```
H,0,0.8208009077,0.6699317781,-2.5564251696
H,0,-0.2239716632,1.7782285744,-2.3785232602
Fe,0,0.0019260086,0.5403423512,0.0260839582
```

{35} $[Fe(AG_D)_2(H_2O)_2]^{3+}$ ls (mirror image)

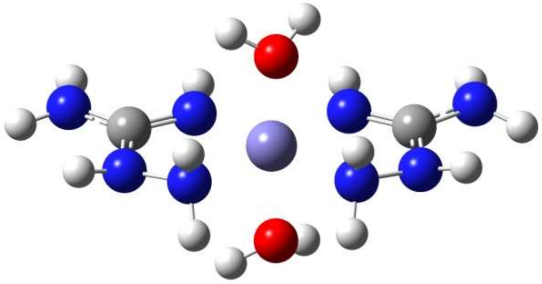

```
Charge =  3 Multiplicity = 2
N,0,1.5446656477,-1.4708990296,0.1587986123
H,0,1.5618376844,-1.8939533013,1.0865191231
H,0,1.4472673136,-2.2161820151,-0.5277261483
N,0,1.4532879459,1.082041881,-0.0557788457
H,0,1.3885502997,2.0908363503,-0.0981155441
C,0,2.656965079,0.5720042061,-0.0571846078
N,0,2.7477987608,-0.7881388873,-0.0838419883
H,0,3.58824004,-1.2317539656,0.264703635
N,0,3.7964001781,1.2734416884,-0.0439701831
H,0,3.7469696075,2.2602517025,-0.2421116572
H,0,4.6668477123,0.8054433391,-0.2416709006
N,0,-1.5666190692,-1.4713248526,0.1486644804
H,0,-1.4875058318,-2.2119256243,-0.5460074401
H,0,-1.5977304437,-1.9132569323,1.0677837095
N,0,-2.7599244086,-0.7673090934,-0.0779141248
C,0,-2.6492336533,0.5833134316,-0.0741708408
N,0,-1.4248733124,1.0715669059,-0.0749897904
N,0,-3.7564027451,1.3177464081,-0.0964712921
H,0,-3.6843947613,2.3181932823,-0.1877854916
H,0,-4.6637410315,0.8853222663,-0.168462328
H,0,-1.3320444858,2.0782040604,-0.1254429134
H,0,-3.6207166693,-1.2122872854,0.2133206097
O,0,0.0742814502,-0.439996563,-1.9041410226
H,0,-0.7630789414,-0.4686349723,-2.3867049057
H,0,0.6130818602,0.2267405561,-2.3521286435
O,0,0.0639395757,-0.0962617179,2.0036500328
H,0,-0.7751898924,-0.0413410273,2.4815597684
H,0,0.6053692947,0.6352522763,2.3311915971
Fe,0,-0.0139102038,-0.168062087,0.0415000998
```

{36} $[Fe(AG_C)(AG_D)(H_2O)_2]^{3+}$ hs

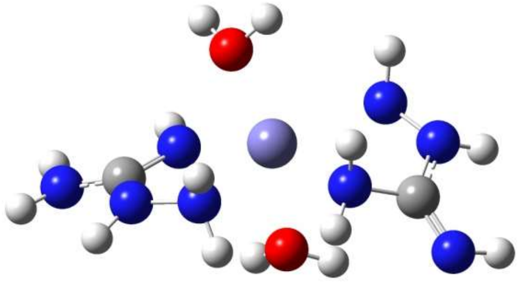

```
Charge =  3 Multiplicity = 6
N,0,2.0368710946,1.6303979763,0.5479948397
H,0,1.955675802,2.4673584224,-0.0246075699
H,0,2.1761803301,1.9199150626,1.5125702336
N,0,3.5805285648,-1.0957398541,-1.1533055219
H,0,4.3365240243,-0.5262793527,-1.5287827792
C,0,2.8808880793,-0.4116770688,-0.3355121959
N,0,3.1226211972,0.8610147956,0.1148101675
H,0,3.8548335184,1.3739055597,-0.3582282106
N,0,1.7154860809,-0.9748924842,0.2565017804
H,0,1.8724632074,-1.1293949041,1.2533549011
H,0,1.5342011523,-1.8764988722,-0.1732570776
N,0,-1.1190603015,-1.4392081767,0.2283315878
H,0,-0.778376709,-1.9644265796,-0.5751195122
H,0,-0.9601452399,-1.9915679207,1.0679847885
N,0,-2.481840439,-1.1912793838,0.0855115164
C,0,-2.8408272701,0.0940038351,-0.1412623709
N,0,-1.9076713684,1.0135738766,-0.0036656207
N,0,-4.1060545349,0.3484936577,-0.4633105097
H,0,-4.4143509306,1.3027484594,-0.5539102907
H,0,-4.7932550859,-0.3882386266,-0.4950172304
H,0,-2.2349765037,1.9635577797,-0.1337256169
H,0,-3.0648558803,-1.9470398745,-0.2498050804
```

```
O,0,-0.5717158746,0.5169234324,2.4139988546
H,0,0.1674549317,0.5944553598,3.0299741802
H,0,-1.1826705714,1.2286156003,2.6388589308
O,0,0.0971885084,0.2160186678,-1.8240763714
H,0,0.9077957708,0.491003078,-2.2714603766
H,0,-0.6129890703,0.6895256574,-2.2747140594
Fe,0,0.0007402073,0.504978397,0.3433107638
```

{37} $[Fe(AG_C)(AG_D)(H_2O)_2]^{3+}$ ls

```
Charge =  3 Multiplicity = 2
N,0,-1.2753337822,-1.5354330382,0.2923814045
H,0,-1.1248569012,-2.2331864932,-0.436762973
H,0,-1.13223646,-1.9880322572,1.1940542359
N,0,-1.6108740451,0.9244775219,-0.293804636
H,0,-1.7087989137,1.9019333007,-0.5395011063
C,0,-2.7271794022,0.2280526003,-0.1740131363
N,0,-2.58968225,-1.0545673978,0.2283016957
H,0,-3.3321243044,-1.7301138833,0.1038119515
N,0,-3.9440113278,0.70647351,-0.3970185323
H,0,-4.0612746452,1.677572936,-0.637515315
H,0,-4.7623526886,0.1397638018,-0.2379137225
N,0,1.2150716462,1.5868530448,-0.1690101154
H,0,1.0997210423,1.9638558711,-1.1106800647
H,0,0.9694265328,2.3262226689,0.4879177324
N,0,2.5603144838,1.2222104002,0.036804733
C,0,2.8710718297,-0.1123834395,-0.014434295
N,0,3.9801963412,-0.6602021204,-0.3060174334
N,0,1.7518108351,-0.9227135246,0.4073234758
H,0,1.8303976326,-1.8371856424,-0.0335849176
H,0,1.8284380926,-1.0786511286,1.4138531885
H,0,4.6515810938,0.0422036391,-0.6118937597
H,0,3.2334629888,1.8614391053,-0.3680930119
O,0,-0.0981497604,0.3052184406,2.0083536325
H,0,0.509635169,0.9498022336,2.3962985926
H,0,-0.9697448424,0.5045629631,2.3771286543
O,0,0.2186924592,-0.5642849788,-1.8139461619
H,0,0.8844944322,-0.1259309297,-2.3621941444
H,0,-0.5928792546,-0.565468139,-2.3392234192
Fe,0,-0.0000817316,0.0117971153,0.0605401181
```

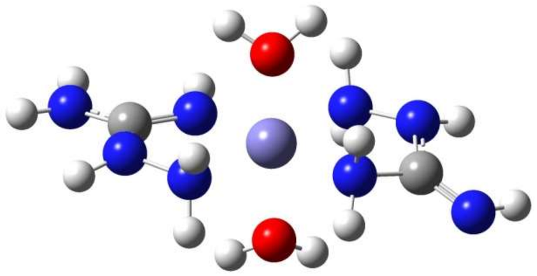

{38} $[Fe(AG_D)_2(H_2O)_2]^{3+}$ hs (cis)

```
Charge =  3 Multiplicity = 6
N,0,0.0965693006,0.6148252465,1.9562777917
H,0,-0.7911005902,1.0669443891,2.1560503329
H,0,0.2504220422,-0.1054199783,2.6601870813
N,0,1.6637757469,0.8068888769,-0.1000529436
H,0,2.3070601187,0.9038410674,-0.8762416244
C,0,1.9646969363,1.567178932,0.9308554091
N,0,1.1213142516,1.5517454929,1.9899027418
H,0,1.4124051832,1.920761791,2.8852324707
N,0,3.0225674957,2.3775831522,0.9885799878
H,0,3.7031233742,2.3563029902,0.2471417456
H,0,3.2501034473,2.8696284462,1.8379036292
N,0,-1.1776708603,1.4078844583,-0.6989534014
H,0,-0.8017737238,2.2435921478,-0.2592341362
H,0,-1.1171809733,1.5294310162,-1.7085741851
N,0,-2.5001825006,1.228532023,-0.3097656219
C,0,-2.841871403,-0.0102113377,0.1194640998
N,0,-1.8723493957,-0.8761946372,0.3207939103
N,0,-4.1374825622,-0.2670196541,0.3068727597
H,0,-4.4083408428,-1.1231588154,0.7619074548
```

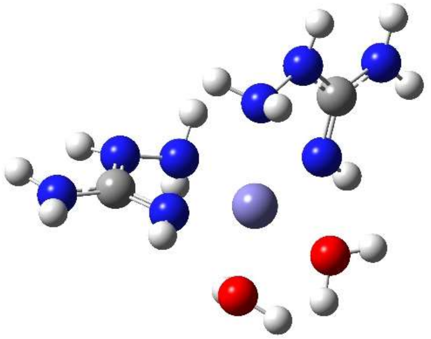

 H,0,-4.824165947,0.4653769193,0.2180897416
 H,0,-2.193218305,-1.7710301022,0.6709247075
 H,0,-3.2028221484,1.8425158811,-0.6994966211
 O,0,-0.1407170217,-1.1077149905,-2.1936701858
 H,0,-0.1913678089,-0.4143208384,-2.8630258237
 H,0,0.6481149368,-1.6165839202,-2.4175865899
 O,0,1.3437231199,-2.1274181076,0.2379586999
 H,0,2.281783806,-1.9149132697,0.1705070038
 H,0,1.1883563813,-2.8306100414,-0.4044520069
 Fe,0,0.0589576921,-0.4388998172,-0.0975855976

{39} $[Fe(AG_D)_2(H_2O)_3]^{3+}$ hs

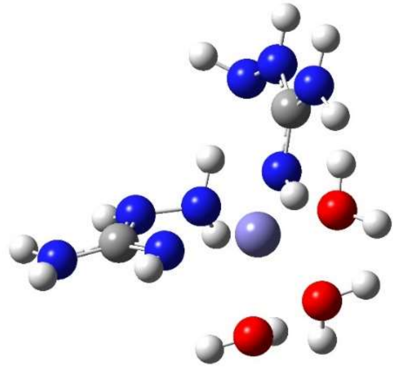

```
Charge =  3 Multiplicity = 6
 N,0,0.1370536544,2.5687584709,1.3297178963
 H,0,0.6290467426,3.2880382428,0.8096931655
 H,0,-0.6869774893,2.990285414,1.7466436904
 N,0,0.8493033748,-0.1575839962,1.6668843158
 H,0,1.1553591396,-1.0654462297,2.0016516864
 C,0,1.2417165314,0.7920286389,2.5025096139
 N,0,0.9647660489,2.0966132014,2.3307111313
 H,0,1.302587501,2.7445861496,3.0299287122
 N,0,1.9372071292,0.4905494401,3.6149940161
 H,0,2.3723035016,-0.4144686944,3.6834059572
 H,0,2.3224998491,1.2293751824,4.1818374912
 N,0,-1.3447178403,1.2272080021,-1.0604674622
 H,0,-1.0455328152,2.1337178163,-0.7135807747
 H,0,-1.2255490854,1.1941801734,-2.069574365
 N,0,-2.6717369927,0.9976446978,-0.7186892713
 C,0,-2.929810103,-0.1475668114,-0.0432141781
 N,0,-1.8977968394,-0.8299849163,0.4019819976
 N,0,-4.2030211646,-0.491473021,0.1455115204
 H,0,-4.4218087581,-1.2851722142,0.7250162328
 H,0,-4.9532562537,0.1123083914,-0.1505118487
 H,0,-2.1451464073,-1.6344272027,0.965698275
 H,0,-3.3893907474,1.423565491,-1.2896843453
 O,0,-0.589791313,-1.2861426369,-2.4030113509
 H,0,-1.540213664,-1.3545693209,-2.5420314851
 H,0,-0.2755032372,-0.7176686493,-3.113606229
 O,0,1.242563598,-2.1354127773,-0.336549644
 H,0,2.1758449303,-1.8906093362,-0.3368637842
 H,0,1.0667761462,-2.4858256949,-1.2189907246
 Fe,0,-0.0176167393,-0.3607816895,-0.1400414601
 O,0,1.4764629099,0.7931482207,-1.2692194371
 H,0,2.3030290956,0.2955779918,-1.2627553654
 H,0,1.6795696469,1.6243785663,-0.8229973565
```

{40} $[Fe(AG_D)_2(H_2O)_4]^{3+}$ hs

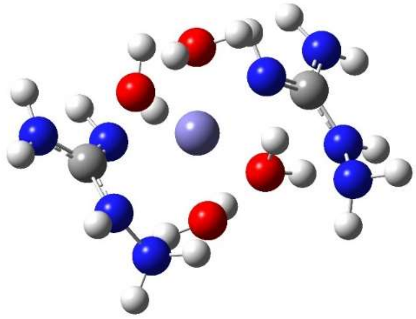

```
Charge =  3 Multiplicity = 6
 N,0,-0.3902045582,2.4594614487,-2.1029710284
 H,0,-0.9192520547,2.8093016699,-2.8974128687
 H,0,-0.1733107537,3.2544609465,-1.5099756222
 N,0,0.3758721898,-0.2793343079,-1.8980747179
 H,0,0.6432613434,-1.2242569904,-2.1529512081
 C,0,1.0412939442,0.5936858498,-2.6287840932
 N,0,0.812832062,1.9282958807,-2.5645975001
 H,0,1.3525394163,2.5113939123,-3.1904419074
 N,0,1.9915638995,0.2127586765,-3.5019735192
 H,0,2.4207233769,-0.6911223094,-3.3887237842
 H,0,2.4822527265,0.894581365,-4.0588323867
 N,0,-1.8433852645,1.0766934481,2.7012642384
```

```
 H,0,-1.9578290141,1.6251883187,3.5455540844
 H,0,-2.4131356685,1.484709924,1.9690087017
 N,0,-2.2777980067,-0.2152563634,2.9596592438
 C,0,-1.5091558346,-1.2887645345,2.7037313488
 N,0,-0.4720703092,-1.2236858834,1.8776046335
 N,0,-1.8405667954,-2.4272438198,3.3298036526
 H,0,-1.4685924709,-3.3019967494,2.9988672113
 H,0,-2.5875209053,-2.4480006077,4.0054796773
 H,0,0.0726706001,-2.0782664263,1.9055818031
 H,0,-3.0947548164,-0.3490344055,3.5404796954
 O,0,0.9420347683,1.6246468956,0.7523421311
 H,0,1.697576953,1.6994114385,0.1568970959
 H,0,1.325967629,1.4571079419,1.6208604283
 O,0,1.833843923,-1.3702001741,0.3740124198
 H,0,2.4864269928,-0.7056589633,0.1255454326
 H,0,1.9367292647,-2.0668928031,-0.2838431857
 Fe,0,-0.2311257528,-0.2460900337,0.1297127604
 O,0,-1.4729964216,-1.9761756098,-0.635961745
 H,0,-2.1774545194,-2.2119699627,-0.0214633957
 H,0,-1.9179595856,-1.7611870385,-1.4641869927
 O,0,-1.8948124889,0.9940017484,-0.323558184
 H,0,-1.493521,1.5432868984,-1.0479637731
 H,0,-2.5660177088,0.4458269096,-0.746936406
```

{41} $[Fe(AG_B)(H_2O)_4]^{3+}$ hs

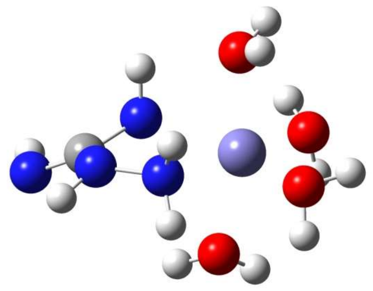

```
Charge =  3 Multiplicity = 6
 N,0,-0.7195976014,-1.5279132811,-0.2784839294
 H,0,-0.6126426967,-1.8296834616,-1.2459922312
 H,0,-0.4751442261,-2.3103831099,0.3238799279
 N,0,-2.0443080739,-1.1390649788,-0.0406099106
 C,0,-2.2868214809,0.1974761169,0.1602677595
 N,0,-3.3945351852,0.7401436993,-0.1568815461
 H,0,-3.4253467399,1.6996088502,0.1770522925
 H,0,-2.7362239168,-1.6220362074,-0.5990728398
 N,0,-1.16747405,0.8334061022,0.7804444883
 H,0,-1.2648687586,1.8442905252,0.7427032952
 H,0,-1.1147256849,0.5534527406,1.7597171808
 O,0,0.2045989422,0.6344479335,-1.9702367763
 H,0,-0.6363533982,1.0802283405,-2.1342240285
 H,0,0.8773296723,1.225808708,-2.3331719578
 O,0,0.8969166696,-0.6318341533,2.0054046797
 H,0,1.4338088479,-0.0476670152,2.5569140879
 H,0,1.3292237396,-1.4956549128,2.0414871236
 Fe,0,0.7997147737,0.0801288089,0.0105517846
 O,0,2.0290971715,-1.5090253901,-0.6570695341
 H,0,2.956347503,-1.3311494686,-0.4517865574
 H,0,1.9860043174,-1.6004165902,-1.6183677101
 O,0,2.067483218,1.7705483757,0.2625355729
 H,0,2.1522705657,2.2466167955,-0.5734311135
 H,0,1.6912863919,2.4048545729,0.886153942
```

{42} $[Fe(AG_C)(H_2O)_4]^{3+}$ hs

```
Charge =  3 Multiplicity = 6
 N,0,-0.7911508159,-1.4923617232,-0.5410847767
 H,0,-0.6783140381,-1.6302291686,-1.5441327704
 H,0,-0.6078503352,-2.3798620725,-0.0785969886
 N,0,-2.0898025136,-1.0646920739,-0.2551232632
 C,0,-2.2567056551,0.2329142007,0.1571396376
 N,0,-3.2893883743,0.966732336,0.0233148065
 N,0,-1.1152204023,0.7045929415,0.8651492119
 H,0,-1.1054121168,0.3002886934,1.8015856563
```

```
 H,0,-1.1715151658,1.7139169701,0.9629973151
 H,0,-3.9884415722,0.4848737617,-0.5396063797
 H,0,-2.8013898809,-1.4287655517,-0.8761950098
 O,0,1.1536318106,-1.0929319943,1.7576781635
 H,0,0.4270292475,-1.5766585893,2.1713756346
 H,0,1.5467898134,-0.5451248719,2.4507095793
 O,0,0.0683031479,1.0866887305,-1.6658336918
 H,0,0.1936275118,2.035595855,-1.5327975299
 H,0,0.5530631279,0.868183613,-2.4720382539
 Fe,0,0.8604711047,0.002937321,0.0174590377
 O,0,2.4638207628,-0.794644856,-1.1436376927
 H,0,2.401486657,-1.7521031084,-1.2510443283
 H,0,2.4724530043,-0.431392486,-2.0382803626
 O,0,2.0018327125,1.6772993898,0.7255997787
 H,0,1.9660386485,1.7347603228,1.6889952909
 H,0,1.6135820712,2.5006388705,0.4008641056
```

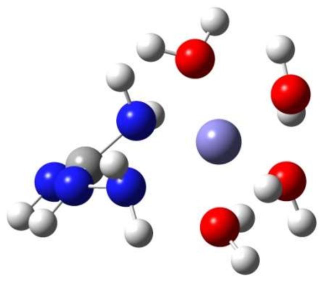

{43} $[Fe(AG_D)(H_2O)_4]^{3+}$ hs

```
Charge =  3 Multiplicity = 6
 N,0,-1.1465853804,1.5332979986,-0.4133723994
 H,0,-0.9721717466,2.243384376,0.2951941437
 H,0,-0.8228509186,1.8788497529,-1.3125512291
 N,0,-2.4976388402,1.2298367599,-0.4802349384
 C,0,-2.878520334,0.0116239116,-0.0469203203
 N,0,-1.9235295094,-0.8436795541,0.2765714047
 N,0,-4.1776351035,-0.2626942127,0.0204296087
 H,0,-4.4796775106,-1.1862306615,0.2841307533
 H,0,-4.867706261,0.4142034625,-0.2651566577
 H,0,-2.2619004582,-1.7501224053,0.5781643349
 H,0,-3.1673972522,1.9789222642,-0.5949658208
 O,0,-0.0324398529,-0.6368810769,-2.0504636037
 H,0,-0.9142805353,-0.7602447885,-2.4206547034
 H,0,0.3545483756,0.1007071252,-2.5371409839
 O,0,0.2025882536,-0.0553189784,2.2695946114
 H,0,-0.4640583599,-0.5286006902,2.7812413615
 H,0,0.0471878474,0.8791890438,2.4560196526
 Fe,0,0.0141723483,-0.3979915602,0.1280754121
 O,0,1.2589392112,-2.1300581169,0.2524188156
 H,0,1.5881093293,-2.3802841901,-0.6194855318
 H,0,2.0423559127,-1.942009375,0.7839971031
 O,0,1.7099375954,0.9590123516,-0.1401996408
 H,0,1.6620028345,1.7531472973,0.4057013269
 H,0,1.7902524748,1.2748135362,-1.0485202594
```

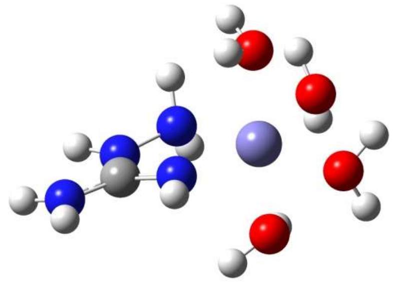

{44} $[Fe(AG_A)(H_2O)_5]^{3+}$ hs

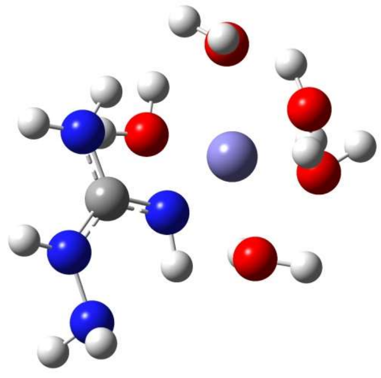

```
Charge =  3 Multiplicity = 6
 N,0,-3.5939782695,-1.3231412276,-0.0375897034
 H,0,-4.0772310119,-1.5770584238,-0.8919250225
 H,0,-4.1866370782,-1.5632347911,0.7493416659
 N,0,-3.3581839487,0.0407885482,-0.0354780022
 C,0,-2.1012927219,0.5117099401,0.0585434882
 N,0,-1.0739713646,-0.3197065107,0.1665431075
 N,0,-1.9366237046,1.8379601012,0.0692626673
 H,0,-2.7104937368,2.4471218346,-0.1438521289
 H,0,-1.0048367329,2.2145876342,0.0173546921
 H,0,-1.408270558,-1.2810679808,0.1521554254
 H,0,-4.1286305908,0.6946849198,-0.0904408677
 O,0,1.0917905022,-0.8479860475,2.0682441373
 H,0,2.0181288289,-0.812191533,2.3385670793
```

H,0,0.6210372462,-0.2723622273,2.6834591659
O,0,0.8568010021,0.637322527,-1.9978616986
H,0,1.5576353767,1.2909827215,-2.1130415216
H,0,0.0357943504,1.1149503317,-2.1689924422
Fe,0,0.9100899164,-0.1432941835,0.0662157746
O,0,0.8976031153,-2.1912092721,-0.6836742929
H,0,1.3291850546,-2.192389295,-1.5472590429
H,0,1.4621725386,-2.7359308499,-0.1216919831
O,0,3.1486509347,-0.2631675376,-0.1598967868
H,0,3.4900092482,0.5603185499,-0.5263901274
H,0,3.3502729183,-0.9378181469,-0.8183655251
O,0,1.4438020188,1.8617166269,0.7415332199
H,0,1.3098785731,2.5097140914,0.0392156849
H,0,0.8955011033,2.1575662004,1.4785990469

{45} $[Fe(AG_B)(H_2O)_5]^{3+}$ hs

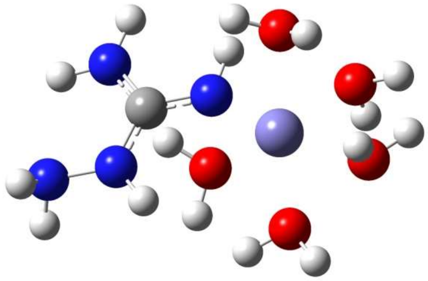

Charge = 3 Multiplicity = 6
N,0,-4.3679831777,2.2399742047,0.4371915572
H,0,-4.4647987714,2.5645101012,-0.5190568555
H,0,-4.4336034757,3.0435100998,1.0519629526
N,0,-3.1234346779,1.6512597358,0.5942768201
C,0,-3.0190024914,0.3557705793,0.9292830112
N,0,-1.8146721352,-0.2126144563,1.035016419
H,0,-1.8849952966,-1.1710068859,1.3613117438
H,0,-2.2808369487,2.1855455695,0.4350948425
N,0,-4.1399386103,-0.3250800454,1.1544348
H,0,-4.1029218654,-1.3002219518,1.3988176075
H,0,-5.0243690594,0.1535479304,1.0795579497
O,0,-0.3701379957,1.4335431804,-1.207476354
H,0,-1.1876614373,1.1982551519,-1.6635731804
H,0,-0.4378465297,2.3742414806,-1.0046197613
O,0,0.5957429709,-1.2318293916,2.0594932524
H,0,0.6185859152,-0.785219401,2.9144699019
H,0,1.4848412541,-1.5832568967,1.9364501031
Fe,0,0.0538334079,0.234958299,0.4876112191
O,0,2.2367945915,0.3566336034,0.0611834296
H,0,2.7206163555,-0.38309792,0.445700096
H,0,2.3784533592,0.2775599144,-0.8892565471
O,0,0.2381816644,1.848258133,1.8701497685
H,0,1.0385533056,2.3600668917,1.6994197529
H,0,-0.4842243361,2.4868430837,1.8806555457
O,0,0.0279032171,-1.5570691524,-0.8362800576
H,0,0.8390502772,-1.5414304993,-1.3586437085
H,0,-0.6857963902,-1.4812156785,-1.4809605386

{46} $[Fe(AG_C)(H_2O)_5]^{3+}$ hs

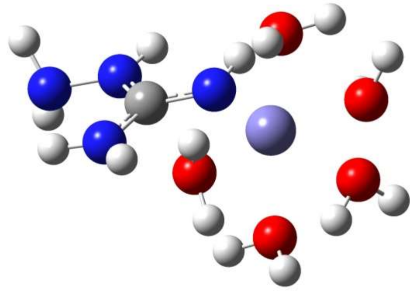

Charge = 3 Multiplicity = 6
N,0,4.4678423173,-0.0597663816,0.0158596127
H,0,4.9606970499,-0.2205075955,-0.8558349605
H,0,5.0395752435,-0.4141529904,0.7745221682
N,0,3.2659066697,-0.748330535,-0.0059764533
C,0,2.1001142589,-0.0913293471,0.1080316408
N,0,0.9475390358,-0.7697720639,0.1051315447
N,0,2.1268145711,1.2323140881,0.2201480139
H,0,3.0243041346,1.6900880345,0.2568078024
H,0,1.2726907202,1.7429672512,0.3910917372
H,0,1.077120956,-1.7734251411,0.0370091749
H,0,3.2474207307,-1.7521998368,-0.131610795
O,0,-0.7403034793,0.6748099215,-2.009967516
H,0,0.1001569214,1.1347533258,-2.1289469893
H,0,-1.420432146,1.3486930768,-2.1339434679

O,0,-1.5002265273,-0.6779589937,2.0388587876
H,0,-1.8921137781,0.0673409917,2.5105480629
H,0,-2.199924626,-1.3420718166,1.9915828379
Fe,0,-0.9172495042,-0.0651476739,0.0534094869
O,0,-1.5228692122,-2.0415375228,-0.6974800391
H,0,-0.8299173309,-2.5890025259,-1.0846317559
H,0,-1.9230637994,-2.5814153572,-0.0058516743
O,0,-3.2205050754,0.3933263523,-0.3293722339
H,0,-3.4443971073,-0.0494350581,-1.1554205607
H,0,-3.2786487482,1.3326272363,-0.5362886333
O,0,-0.8089319781,2.0144008849,0.6963043025
H,0,-1.3116777335,2.5725088194,0.0903982298
H,0,-1.2160292233,2.1317232171,1.5636437766

{47} $[Fe(AG_D)(H_2O)_5]^{3+}$ hs

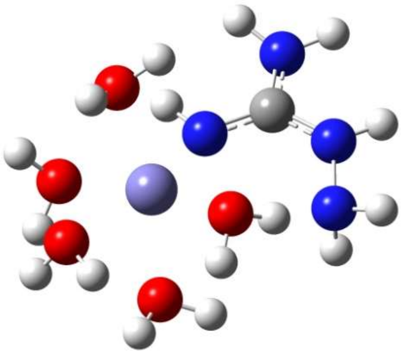

Charge = 3 Multiplicity = 6
N,0,-3.2065706027,0.279269288,1.163119138
H,0,-3.5840478787,0.4041254402,2.0965970145
H,0,-3.6388890819,0.9859023064,0.570660013
N,0,-3.6038948306,-0.9882839547,0.7399508638
C,0,-3.0016459669,-1.6519576807,-0.2609996153
N,0,-1.8424326648,-1.2799160804,-0.7963958325
N,0,-3.6583938208,-2.7231593521,-0.7323711184
H,0,-3.1579220767,-3.3859087995,-1.3024391608
H,0,-4.5014788975,-3.0441649201,-0.2819481544
H,0,-1.6108448819,-1.8690700236,-1.5898605035
H,0,-4.5494266827,-1.2704267507,0.9636159893
O,0,0.330394017,-0.5703153239,-2.4016754971
H,0,0.5347767481,0.265390408,-2.8386704547
H,0,1.1426239955,-1.0894476197,-2.452135438
O,0,-0.4406873726,0.4138495666,1.7189980508
H,0,-1.4221856026,0.5001725059,1.7111248841
H,0,-0.0986930529,1.3119518373,1.8047718755
Fe,0,-0.1982563851,-0.2223746763,-0.3157839744
O,0,0.8545876869,-2.0387909643,0.2313790299
H,0,1.7158780133,-1.7901761265,0.5902045768
H,0,0.3981346491,-2.4881643717,0.9531469338
O,0,1.9525353253,0.7518161595,-0.1108881716
H,0,1.9212515673,1.4183340983,0.5840324586
H,0,2.0899217499,1.2541300116,-0.9217339366
O,0,-0.7889798447,1.7302233937,-1.0536836022
H,0,-1.5851165462,2.0447065633,-0.6071706441
H,0,-0.0980586331,2.3615121354,-0.8173802944

{48} $[Fe(AG_A)_2(H_2O)_2]^{3+}$ hs (5-coord.)

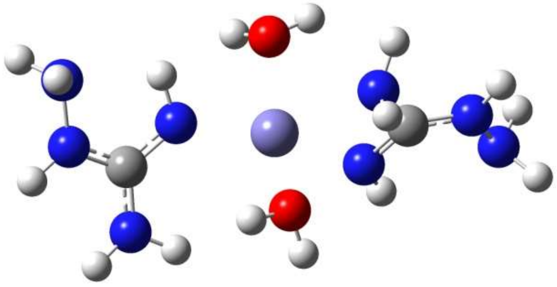

Charge = 3 Multiplicity = 6
N,0,4.3338749569,0.3772023402,-1.2290133701
H,0,4.5939510954,-0.3390718569,-1.8986341092
H,0,5.1077046122,1.025250108,-1.1364564106
N,0,4.0823207379,-0.211345466,-0.001234924
C,0,2.848470979,-0.1616009303,0.5313246875
N,0,1.8717817863,0.4927672271,-0.0806410948
N,0,2.65512633,-0.75977273,1.7129175149
H,0,1.709766672,-0.8748309857,2.0404061174
H,0,3.3500731832,-1.3975707767,2.068639253
H,0,2.2335964231,0.9408935582,-0.9210383989
H,0,4.807146133,-0.732413754,0.4754365387
N,0,-4.7696684077,1.0122913703,0.7410693211

H,0,-5.495650092,0.3160792744,0.8744599115
H,0,-5.2201847951,1.9062464384,0.5755757548
N,0,-4.0321590692,0.6744703384,-0.3855425873
C,0,-2.7343464282,0.4365896466,-0.2718275468
N,0,-2.0174061127,0.4451776574,0.8101207923
N,0,-1.9533781798,0.170773316,-1.4210604917
H,0,-1.9873553419,-0.8160515022,-1.6676439036
H,0,-2.2152830707,0.730542738,-2.2277285707
H,0,-2.5144770999,0.6239834664,1.6760527229
H,0,-4.4731075477,0.6004121816,-1.2955461269
O,0,-0.1125049551,2.4117138668,-0.6982302533
H,0,-0.9581295012,2.8659123536,-0.8037864793
H,0,0.4435564914,3.0094337484,-0.1829389203
O,0,-0.1951341506,-1.7301457364,0.0927119497
H,0,-0.5521845894,-2.0670782153,0.9236287051
H,0,0.6461799888,-2.1847680739,-0.0355507132
Fe,0,-0.1126217181,0.4258892177,0.0341645817

{49} $[Fe(AG_A)_2(H_2O)_2]^{3+}$ hs (4-coord.)

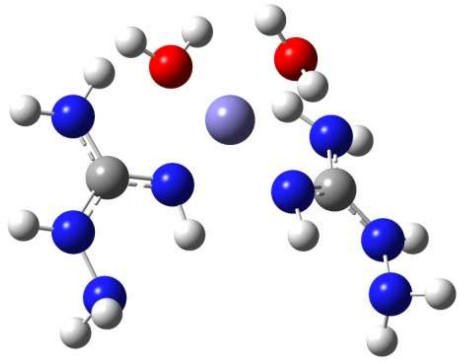

Charge = 3 Multiplicity = 6
N,0,3.5392158602,-1.82853003,-1.1125128518
H,0,4.1017289856,-2.4646552119,-0.5583347897
H,0,3.8164721801,-1.9176809645,-2.0836307645
N,0,3.7659886494,-0.5295111141,-0.6905169764
C,0,2.747613058,0.2173165816,-0.2336207536
N,0,1.5132610761,-0.2654099291,-0.2041907992
N,0,3.0263315542,1.4484017418,0.2175425956
H,0,3.9385853504,1.8399167964,0.0414474995
H,0,2.2629858048,2.0982718356,0.3150494197
H,0,1.4980329568,-1.211560118,-0.5802305586
H,0,4.6947350125,-0.1302429514,-0.7380652276
N,0,-2.7197219913,-2.1221880802,-2.2685081336
H,0,-2.764568878,-1.891703572,-3.2549912658
H,0,-3.1828511721,-3.0126346093,-2.125571917
N,0,-3.3768247489,-1.1447972579,-1.540435195
C,0,-2.7073233474,-0.4170831171,-0.6298234471
N,0,-1.4291783998,-0.6568093262,-0.3695544181
N,0,-3.3722054609,0.5619788096,-0.008369168
H,0,-4.3621588565,0.6803431951,-0.1521439013
H,0,-2.9435313691,1.0332496608,0.7705612998
H,0,-1.0862489045,-1.4194237127,-0.9491902953
H,0,-4.351300498,-0.9405773024,-1.7205402915
Fe,0,-0.1226587245,0.3983066901,0.6860893122
O,0,-0.3982334845,2.4497139015,0.4667857491
H,0,-1.2990400712,2.7394344595,0.6604632409
H,0,0.1754933355,2.9771139216,1.038280338
O,0,-0.0087074604,-0.3671900314,2.653073309
H,0,-0.0855672761,-1.3295165894,2.6596672984
H,0,-0.7407997805,-0.0470626757,3.1951768118

{50} $[Fe(AG_A)(H_2O)_4]^{3+}$ hs (5-coord.)

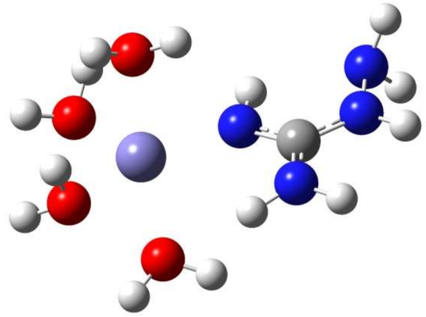

Charge = 3 Multiplicity = 6
N,0,3.5300455237,-0.580285356,0.8940646044
H,0,4.1541646082,0.0550481875,1.3791082189
H,0,4.0269761064,-1.4467862848,0.71854504
N,0,3.1575854791,-0.0161588063,-0.3139553424
C,0,1.8621203837,0.2081488346,-0.5834279961
N,0,0.9189487433,-0.0856346493,0.3033407195
N,0,1.5497352844,0.7131784494,-1.7801816031
H,0,2.2775247395,1.0334907446,-2.3994393845
H,0,0.6129240395,1.04637296,-1.9426583931
H,0,1.3260151265,-0.481972226,1.1481971701

H,0,3.8557966057,0.2290045433,-1.0045374766
O,0,-0.9430928028,-2.0394762556,-0.5091133629
H,0,-1.6841138708,-2.3364113214,-1.0501758886
H,0,-0.1522565064,-2.2280140542,-1.0270538863
O,0,-0.930394406,2.1814734741,0.5185960387
H,0,-1.6402731627,2.6694075558,0.0817501645
H,0,-0.1082897605,2.5785671946,0.2046781556
Fe,0,-1.0434582756,0.0832763235,0.1647351939
O,0,-1.9613198655,-0.4822243062,1.9950636188
H,0,-2.8675704114,-0.7669745633,1.8196114905
H,0,-1.5156390616,-1.25762395,2.3608628628
O,0,-2.6857917907,0.3617219636,-1.1747075249
H,0,-3.496132649,0.5672996471,-0.6917386591
H,0,-2.8808690469,-0.4317211049,-1.6875497902

{51} $[Fe(AG_B)_2(H_2O)_2]^{3+}$ hs (4-coord.)

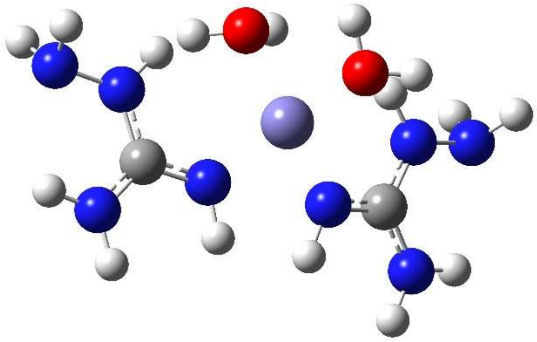

Charge = 3 Multiplicity = 6
N,0,4.8488062858,-0.3296539456,-0.7482797617
H,0,5.2589319892,-0.8374119669,0.0273232244
H,0,5.3400842075,0.5513717852,-0.8505705909
N,0,1.2821931892,-0.2598465806,-1.0356479497
H,0,0.6557209664,-0.5953990868,-1.7595007645
C,0,2.5603328265,-0.5125989842,-1.3206616349
N,0,3.5130800105,-0.0833000352,-0.4796351465
H,0,3.2338383409,0.4670447689,0.32315227
N,0,-3.5269284191,2.7583404243,1.5976201387
H,0,-3.3290912751,3.7434926988,1.4615899379
H,0,-3.6932891264,2.6009615668,2.5856020886
N,0,-2.4302425075,2.0147524183,1.1949918083
C,0,-2.5462227049,1.082292709,0.2370061136
N,0,-1.4687264829,0.3794612855,-0.114481681
H,0,-1.6612160128,-0.2846401398,-0.8567672856
H,0,-1.5308410798,2.1397261813,1.6391662757
N,0,-3.7353980209,0.9025592695,-0.3286897047
H,0,-3.8622269529,0.2076371952,-1.0447072076
H,0,-4.5088889424,1.4709355939,-0.0196810201
N,0,2.9247548173,-1.1677301882,-2.4180795464
H,0,3.904083848,-1.349859837,-2.574596508
H,0,2.2283664658,-1.5581705762,-3.030360182
O,0,1.0687034649,2.2933578432,1.2369722815
H,0,0.545881972,3.0162888322,0.8674522858
H,0,1.9682534183,2.4326838181,0.912979696
O,0,0.6466110753,-0.8499371556,2.3034397734
H,0,0.6584059249,-0.3240411863,3.1130027989
H,0,1.4943014808,-1.3112004747,2.2826664967
Fe,0,0.3832962214,0.380389837,0.604257144

{52} $[Fe(AG_B)(H_2O)_4]^{3+}$ hs (5-coord.)

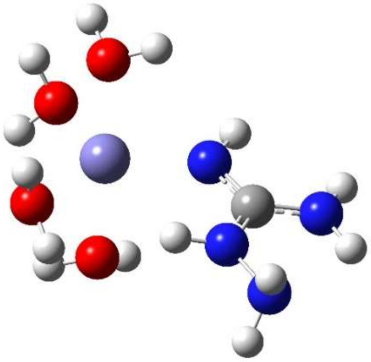

Charge = 3 Multiplicity = 6
N,0,-3.8273583034,-2.2481187635,-0.0464350334
H,0,-3.4825882894,-3.1152412029,0.351613132
H,0,-4.090992508,-2.4241766434,-1.0097394691
N,0,-2.8130709567,-1.3049920486,-0.0201312392
C,0,-2.969856211,-0.1531566554,0.649116419
N,0,-1.9537434268,0.7156112155,0.6932159137
H,0,-2.1937818208,1.5774651267,1.1715406579
H,0,-1.9448088016,-1.4726144715,-0.5120701827
N,0,-4.1357263284,0.0851649925,1.2380470586
H,0,-4.2694048509,0.9154668125,1.7901823533
H,0,-4.8583917128,-0.6164519504,1.1825667729

```
 O,0,-0.2831309104,1.6090396297,-1.5731263021
 H,0,-1.2048125183,1.8549399862,-1.7126442734
 H,0,0.2014078051,2.4423548692,-1.5388376644
 O,0,0.2284071511,-0.8131201593,2.023058918
 H,0,-0.0673470736,-0.3618147625,2.8231214083
 H,0,1.1662606104,-0.9957375342,2.1619267274
 Fe,0,-0.0496987294,0.4286147145,0.2787198525
 O,0,1.3122998593,1.8415747079,0.9891962308
 H,0,2.053321603,1.4317829899,1.453023767
 H,0,1.6974286806,2.3943590524,0.2977470724
 O,0,0.8474418733,-1.1416038718,-0.8327860012
 H,0,0.7719702009,-0.9659620655,-1.7795430523
 H,0,0.3930181079,-1.9815034779,-0.6870921961
```

{53} $[Fe(AG_C)_3]^{3+}$ hs (5-coord.)

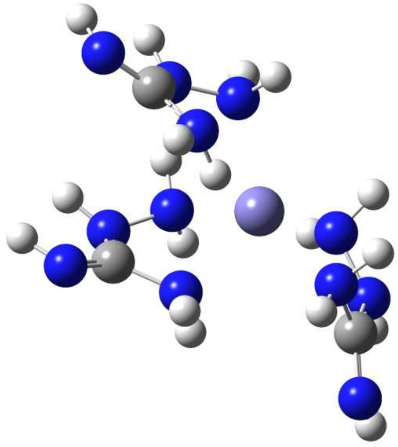

```
Charge =  3 Multiplicity = 6
 N,0,-1.1592642543,-1.7994909704,0.8072420967
 H,0,-0.9972769125,-2.7355431417,0.4301273764
 H,0,-1.0987457189,-1.8747936088,1.8213736179
 N,0,-2.4421268909,-1.3253837103,0.4933153004
 C,0,-2.9543367554,-1.5197476339,-0.788712759
 N,0,-4.2146255073,-1.546246937,-1.034569541
 N,0,-2.0196099539,-1.7012075114,-1.7715496069
 H,0,-1.1490861772,-1.1919267954,-1.6933653745
 H,0,-2.4130618834,-1.6627506566,-2.6995693922
 H,0,-4.7407102606,-1.5165208133,-0.1653555915
 H,0,-3.1103644217,-1.503570906,1.2332996965
 N,0,2.2703488529,-0.5383228628,1.631713221
 H,0,2.1374143601,0.0639545876,2.4424022209
 H,0,2.3933766408,-1.4894900112,1.9747751103
 N,0,3.4261788694,-0.1240699455,0.9426671506
 C,0,3.3295447997,0.1015543976,-0.409262071
 N,0,4.0642351725,0.8413651008,-1.1398774631
 N,0,2.2580082796,-0.6427035107,-0.9822352385
 H,0,2.4618262677,-1.6430153353,-0.9491230546
 H,0,2.1350727773,-0.3776945316,-1.9555705268
 H,0,4.733411966,1.3521096285,-0.5668038956
 H,0,4.0292492176,0.5009078773,1.4615650726
 Fe,0,0.5068460821,-0.4282567182,0.290988173
 N,0,-0.4090382763,1.1079685359,1.6616957989
 H,0,-0.9549789404,0.6451744669,2.3866796602
 H,0,0.3058980217,1.6619170329,2.131527926
 N,0,-1.2535689147,1.9571969253,0.9224593555
 C,0,-1.1503944814,1.9040432314,-0.4482008767
 N,0,-2.0214962483,2.2326277936,-1.3164944067
 N,0,0.1308912734,1.4229667231,-0.8449681231
 H,0,0.8794508347,2.0530522025,-0.5531926241
 H,0,0.1657018763,1.3151182146,-1.8548222935
 H,0,-2.8951343416,2.4844035626,-0.85732549
 H,0,-2.1887682732,2.0474870496,1.2972552717
```

{54} $[Fe(AG_C)_2(H_2O)_2]^{3+}$ hs (4-coord.)

```
Charge =  3 Multiplicity = 6
 N,0,-3.8929502368,-0.7425555817,-2.0184984775
 H,0,-4.6245176515,-0.1674597568,-2.420692244
 H,0,-4.2953324817,-1.6366927411,-1.7593153056
 N,0,-3.3921590253,-0.119426641,-0.886811717
 C,0,-2.0813187819,0.168389223,-0.7865806585
 N,0,-1.6103482741,0.7487942297,0.3174451531
 N,0,-1.29962669,-0.1374014456,-1.8153585676
 H,0,-1.7178872072,-0.5951061904,-2.6105898851
 H,0,-0.3093953805,0.0454136785,-1.7944360917
```

```
H,0,-2.3434191812,0.93022819,0.9973781009
H,0,-3.9919944898,0.0664990816,-0.0938545586
N,0,3.0326994426,2.0428604683,-3.4582867968
H,0,3.9632942665,2.4439117676,-3.4948481482
H,0,2.5114994802,2.3809699874,-4.2602960633
N,0,2.4057587729,2.4700737699,-2.2985343333
C,0,1.9268708575,1.5892874312,-1.4075718616
N,0,1.368429997,2.0115766195,-0.2797739134
N,0,1.991367784,0.2835821964,-1.6965020029
H,0,2.5443133483,0.0256466491,-2.5010836537
H,0,1.9153954604,-0.3742137985,-0.9358303734
H,0,1.406531836,3.0205373647,-0.1735748046
H,0,2.2869366929,3.4579750396,-2.1114896188
O,0,0.0302609346,2.2212667751,2.8490278256
H,0,-0.8267039985,2.6639373554,2.9010361256
H,0,0.6919943375,2.9226900966,2.9039275075
O,0,0.8779198973,-0.8698078452,1.32546366
H,0,1.7410652484,-0.9098163157,1.7569829439
H,0,0.285673087,-1.412595898,1.8608077088
Fe,0,0.2097307552,1.1012798202,1.0781069903
```

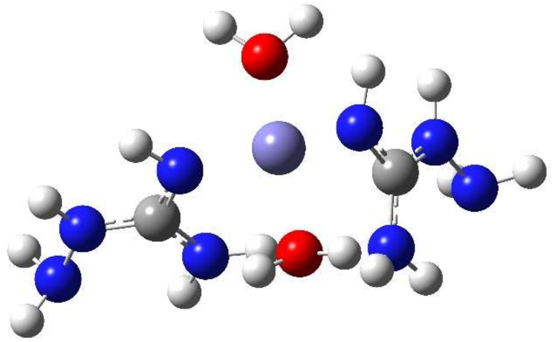

{55} [Fe(AG$_C$)(H$_2$O)$_4$]$^{3+}$ hs (5-coord.)

```
Charge =  3 Multiplicity = 6
N,0,-4.993687563,-1.2613304035,-0.4552954095
H,0,-5.8725289007,-0.7557959874,-0.4313836022
H,0,-5.1418753707,-2.1745834031,-0.0393833102
N,0,-4.0526152769,-0.5729059177,0.2935113745
C,0,-2.8917546703,-0.1864262393,-0.2578650453
N,0,-1.9710709646,0.4292589466,0.4892353843
N,0,-2.6999635792,-0.4014907489,-1.5545420003
H,0,-3.3904471857,-0.94181081,-2.0528201236
H,0,-1.792170246,-0.2369385114,-1.9585727918
H,0,-2.2650335686,0.562583726,1.4518847882
H,0,-4.1964476395,-0.4162427179,1.2826658014
O,0,-0.3484003248,2.700541112,1.2997491965
H,0,-0.9301932654,3.3087260791,0.8272400794
H,0,0.4874753189,3.1726020726,1.3988181953
O,0,0.2430814113,-0.9019730786,-1.0356017477
H,0,1.1827663501,-1.0152961037,-1.2276758264
H,0,-0.0177550008,-1.6915463807,-0.5440161187
Fe,0,-0.0755069807,0.8837894142,0.1157864892
O,0,1.6377981537,0.5367553447,1.3171126003
H,0,2.3281666683,0.1031524585,0.8001801727
H,0,2.0292740273,1.3568729775,1.6433937495
O,0,0.1355076035,2.0666162812,-1.6375467017
H,0,-0.3076825011,1.6422762332,-2.3839364338
H,0,-0.307264495,2.9167506666,-1.5177937201
```

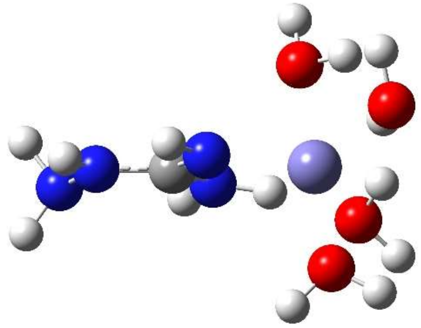

{56} [Fe(AG$_D$)(H$_2$O)$_2$]$^{3+}$ hs (4-coord.)

```
Charge =  3 Multiplicity = 6
N,0,-1.1681678236,1.5388803385,0.3169313967
H,0,-1.0850791466,1.9843742979,1.2285271392
H,0,-0.8131799079,2.1737670143,-0.3944177894
N,0,-2.4845888721,1.2117426594,0.0541334194
C,0,-2.7923687974,-0.0889753833,-0.0590757456
N,0,-1.7921449227,-0.9606368947,0.0224219399
N,0,-4.0565990704,-0.4380263034,-0.255340979
H,0,-4.3053917828,-1.4114012475,-0.3261825548
H,0,-4.7866598005,0.2562841255,-0.2933155251
H,0,-2.0801286937,-1.9288929361,-0.0706183216
```

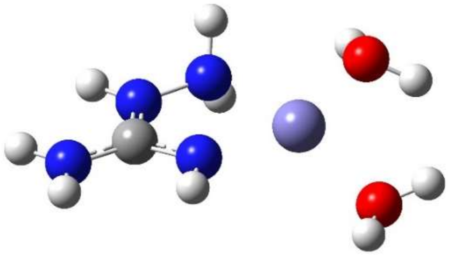

```
H,0,-3.1917279859,1.9348305726,0.0511650794
Fe,0,0.1013591914,-0.4661855082,0.3200135519
O,0,1.3790147716,-1.7200649117,-0.6737867481
H,0,2.2303801285,-1.8220965058,-0.2274120147
H,0,1.0266900859,-2.6113553615,-0.7975227398
O,0,1.5515687538,0.9159182612,0.8939982881
H,0,2.4203457919,0.6515201132,0.5633933824
H,0,1.4719751805,1.8575604995,0.6977100611
```

{57} $[Fe(AG_D)(H_2O)_3]^{3+}$ hs (5-coord., sq. pyr.)

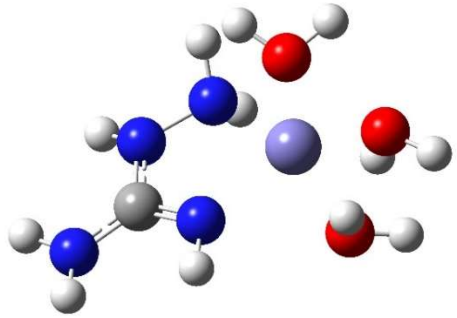

```
Charge =  3 Multiplicity = 6
N,0,-1.2756424864,1.5842197305,0.7562594297
H,0,-1.2849610854,1.9257607399,1.7143327171
H,0,-0.9283670937,2.3266905722,0.1506516929
N,0,-2.5589981723,1.2110194077,0.3780929189
C,0,-2.6932446682,0.0092937928,-0.2199936335
N,0,-1.623843299,-0.7652563608,-0.2539201597
N,0,-3.8742029836,-0.3257347666,-0.7291424472
H,0,-4.0177608995,-1.2672975936,-1.0574059646
H,0,-4.6809454587,0.2624586323,-0.5895333755
H,0,-1.7592798337,-1.6710975996,-0.6858240964
H,0,-3.2591433449,1.9318651515,0.2617577678
Fe,0,0.0911454287,-0.2194017423,0.5909100513
O,0,0.9330211273,-1.9309889145,-0.3034390916
H,0,1.8394411474,-1.7503744646,-0.5840976092
H,0,1.0022923336,-2.6448110487,0.3440923678
O,0,1.5355768028,1.1355794708,-0.0539795808
H,0,2.322027681,0.6651740754,-0.3607333609
H,0,1.2680233223,1.7048847066,-0.7875384697
O,0,0.3215653101,-0.8081359403,2.5735898458
H,0,1.1342039712,-0.433580122,2.939664438
H,0,-0.3919332489,-0.4708249968,3.1317147197
```

{58} $[Fe(AG_D)(H_2O)_3]^{3+}$ hs (5-coord., tr. bipy.)

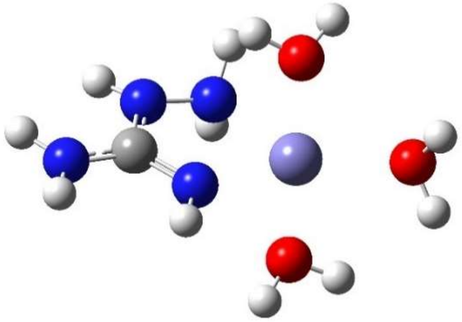

```
Charge =  3 Multiplicity = 6
N,0,-1.1742211197,1.5038485859,0.0233517128
H,0,-0.8839024194,2.1058498994,0.7903473978
H,0,-1.0226080774,1.9963541753,-0.8559439613
N,0,-2.5154534226,1.1722891504,0.1728808083
C,0,-2.8520372833,-0.1237020709,0.0036432649
N,0,-1.8615009483,-0.9902462379,-0.1280224241
N,0,-4.1370996726,-0.4557598944,-0.0106237156
H,0,-4.3989305944,-1.42737871,-0.0590535434
H,0,-4.8587859426,0.2386082299,0.1034949096
H,0,-2.1589838962,-1.9530807623,-0.2364831191
H,0,-3.2050315956,1.9086072332,0.0979524565
Fe,0,0.0344751621,-0.3738920994,0.05110554
O,0,0.1849771783,-0.3405500533,-2.1429944234
H,0,1.0969221532,-0.4458069281,-2.4399049571
H,0,-0.3082675311,-1.0581755386,-2.5574677113
O,0,2.1285972827,-0.5114618687,0.119564479
H,0,2.5215003642,-1.0212482752,-0.5992791345
H,0,2.4672359466,-0.8898019874,0.9402431829
O,0,-0.1139303225,-0.2767132115,2.1652495336
H,0,0.4964315878,0.3483088029,2.5760551215
H,0,-0.9799647194,-0.1083935892,2.5569250129
```

{59} $[Fe(AG_D)_2]^{3+}$ hs (4-coord.)

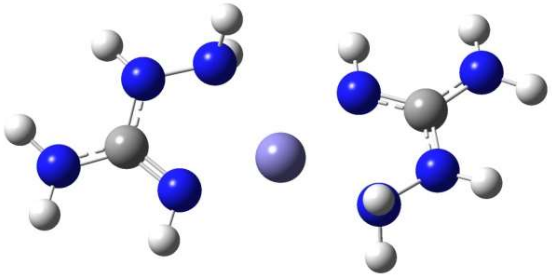

```
Charge =  3 Multiplicity = 6
 N,0,1.8027199736,-1.3810355247,0.6719771805
 H,0,1.8448581142,-1.4695377121,1.6859199636
 H,0,1.7941705528,-2.3160999785,0.2702120691
 N,0,1.4315985949,0.9642773541,-0.3918107087
 H,0,1.3398745009,1.8970727501,-0.7754969867
 C,0,2.6888216594,0.5656630283,-0.2725676763
 N,0,2.9024759935,-0.6806346081,0.1960867305
 H,0,3.8271176733,-0.9971348211,0.4542976724
 N,0,3.7425487994,1.3097648705,-0.5893776029
 H,0,3.6073327425,2.2066769305,-1.0270721165
 H,0,4.6782970824,0.9406928133,-0.5226415604
 N,0,-1.2618427996,1.6034408772,-0.2966015545
 H,0,-0.9820024176,2.3238243574,0.3645655554
 H,0,-1.0673733682,1.938848379,-1.23893214
 N,0,-2.612329162,1.3285284804,-0.1453949906
 C,0,-2.9575790214,0.025948265,-0.0931582795
 N,0,-1.9759406852,-0.8540541884,0.0055470522
 N,0,-4.2488041462,-0.2896671881,-0.1339864128
 H,0,-4.5313644317,-1.2403998127,0.0405549902
 H,0,-4.9542242757,0.4307059748,-0.1293124736
 H,0,-2.2911251409,-1.8154006883,0.0694222356
 H,0,-3.2860099364,2.0375448084,-0.4032871052
 Fe,0,-0.0549053021,-0.3053033669,0.066265158
```

{60} $[Fe(AG_D)_2(H_2O)]^{3+}$ hs (5-coord.)

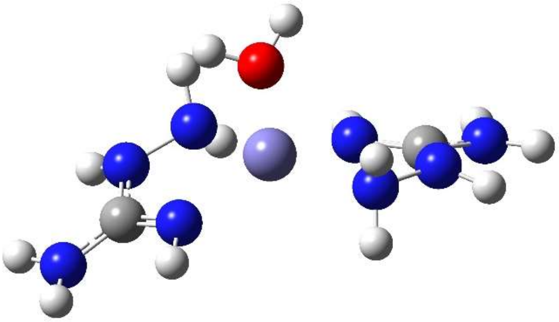

```
Charge =  3 Multiplicity = 6
 N,0,1.7588451212,-1.5665079383,0.5004661466
 H,0,1.8402480824,-2.1600353364,1.3218606036
 H,0,1.6057684791,-2.161845003,-0.3119564359
 N,0,1.5507354712,0.998705826,0.211317601
 H,0,1.4939559676,1.9845458807,-0.0143096876
 C,0,2.7609356862,0.4933694528,0.0669847418
 N,0,2.9241955388,-0.8221126902,0.3470952096
 H,0,3.7461155412,-1.3071046065,0.0106948289
 N,0,3.8276934972,1.1918382598,-0.3090692413
 H,0,3.7513278256,2.1871784647,-0.4411020923
 H,0,4.7367567382,0.7615774146,-0.3726548881
 N,0,-1.2166926451,1.7755807751,0.5906736141
 H,0,-1.2438961338,2.2547710155,1.4872864761
 H,0,-0.78626655,2.3979184254,-0.0895718746
 N,0,-2.5108917486,1.4622246527,0.1920907407
 C,0,-2.7283002526,0.1942978549,-0.221237503
 N,0,-1.7680306995,-0.6861898838,-0.0242539203
 N,0,-3.899632206,-0.0904820346,-0.7864273255
 H,0,-4.1301178277,-1.0501919383,-0.986586987
 H,0,-4.6344394411,0.5981574833,-0.8211161154
 H,0,-2.0087908261,-1.6282015053,-0.3096738552
 H,0,-3.1032103468,2.2122465602,-0.1391483109
 Fe,0,0.0102955311,-0.1789662596,0.7714117112
 O,0,0.0866565609,-0.7490060237,2.8137669266
 H,0,0.6613305375,-0.1648076003,3.3244271579
 H,0,-0.7796160808,-0.7014802857,3.237120729
```

{61} $[Fe(AG_D)_3]^{2+}$ ls

```
Charge =  2 Multiplicity = 1
 Fe,0,-0.0676860227,-0.0362588881,0.0694380924
 N,0,-0.2369683129,1.4740403971,1.4463274309
 H,0,-1.2085915894,1.7710445663,1.5037141329
 H,0,0.0501940573,1.1969131187,2.3804791768
 N,0,0.5641175184,2.5677038252,1.0577052278
 H,0,0.2838220649,3.4645456568,1.4323556479
 C,0,0.9862658623,2.5446142218,-0.2497858447
 N,0,1.4974874467,3.7145193018,-0.7267278268
 H,0,1.8531065018,4.365252709,-0.0418542552
 H,0,2.0831300868,3.6225811347,-1.5427563332
 N,0,0.857737473,1.4401810245,-0.9096631062
 H,0,1.2522684533,1.4762765597,-1.841597065
 N,0,0.3374850939,-1.4697708365,-1.3452291787
 H,0,0.2911278526,-1.0754928337,-2.2797813322
 H,0,-0.3292423689,-2.2383743332,-1.3161737369
 N,0,1.6370104316,-1.9793981949,-1.1530555919
 C,0,2.2075225296,-1.6936014304,0.0625668463
 H,0,1.8103208714,-2.8952880622,-1.5444207517
 N,0,3.3186023293,-2.4178980764,0.3743912589
 H,0,3.8232743832,-2.8315763881,-0.395440447
 H,0,3.9124321414,-2.0146769023,1.0828487541
 N,0,1.6499420361,-0.7872139877,0.7992416397
 H,0,2.1674700857,-0.5812609827,1.6449173556
 N,0,-1.2347474717,-1.3719651964,1.1472429773
 H,0,-1.2994623266,-1.088744221,2.1229256121
 H,0,-0.8452268684,-2.3103729563,1.1398851847
 N,0,-2.5288871788,-1.4334393822,0.5924349946
 H,0,-3.2557057789,-1.7710548595,1.2097237039
 C,0,-2.8522267523,-0.3927694079,-0.2438972398
 N,0,-4.172632582,-0.2657480456,-0.5455211673
 H,0,-4.7503989477,-1.0842356988,-0.4260715094
 H,0,-4.380269038,0.2710072839,-1.373432013
 N,0,-1.8939416367,0.3720601117,-0.6616138366
 H,0,-2.2010133443,1.0777067726,-1.3199788003
```

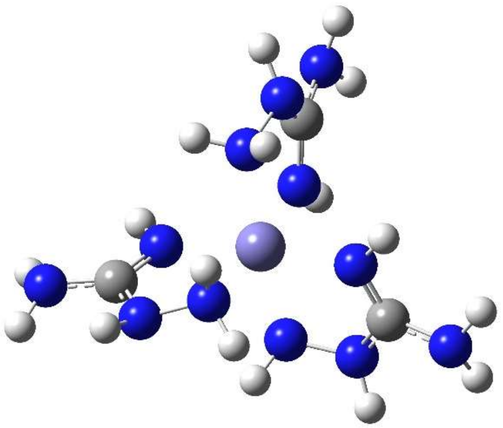

{62} $[Fe(AG_D)_3]^{2+}$ hs (same orientation)

```
Charge =  2 Multiplicity = 5
 Fe,0,-0.1570281909,-0.0905104863,0.0175369383
 N,0,0.165467871,1.7755779088,1.5204775881
 H,0,-0.7030057946,2.293565348,1.6282852316
 H,0,0.4457720455,1.4512152988,2.4396542542
 N,0,1.1613397542,2.605980335,1.0098549321
 H,0,1.2244653556,3.5364713865,1.4003252121
 C,0,1.4574798289,2.4630780054,-0.3206098687
 N,0,2.2038101862,3.4646577356,-0.8559050284
 H,0,2.7117687209,4.0608797902,-0.2200605213
 H,0,2.6915671979,3.248065501,-1.7108539424
 N,0,1.0082872866,1.4300900413,-0.9647194394
 H,0,1.3248310551,1.4211026165,-1.9263941578
 N,0,1.742216754,-0.8332258522,1.3384732248
 H,0,1.4556383107,-1.1342532064,2.2657993383
 H,0,2.4000681901,-0.0688744484,1.4476964981
 N,0,2.3707840568,-1.879675595,0.6691729892
 C,0,1.6851548757,-2.4685553836,-0.3538476057
 H,0,3.0177902073,-2.4522030568,1.194398118
 N,0,2.1963726695,-3.6442866253,-0.7988270643
 H,0,3.1537246516,-3.858447891,-0.5628751613
 H,0,1.9149237076,-3.9291584239,-1.7237618154
 N,0,0.6149433489,-1.8948850799,-0.819796571
```

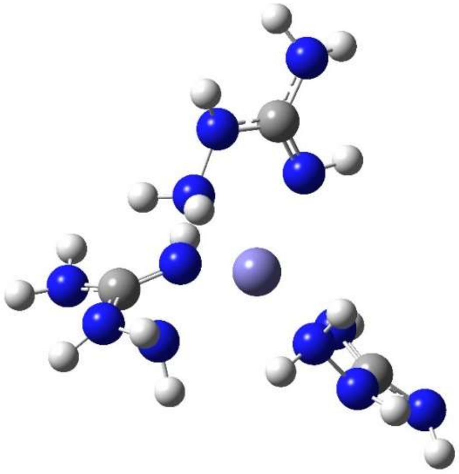

```
H,0,0.2213576478,-2.4079929126,-1.598460964
N,0,-1.963682335,-0.593155503,1.4807299965
H,0,-1.9249711718,-0.0626076722,2.3444329026
H,0,-1.9189739578,-1.5790047624,1.7252928967
N,0,-3.1742200838,-0.3184191979,0.8413102271
H,0,-4.0000124245,-0.759575662,1.2234955063
C,0,-3.1361667159,-0.1302873892,-0.5143970071
N,0,-4.3448529559,-0.1481736242,-1.1342690437
H,0,-5.1664903689,0.0078151008,-0.5697310482
H,0,-4.3765473875,0.2703426653,-2.0505742406
N,0,-1.9921010824,0.0381266228,-1.1053553606
H,0,-2.1087432529,0.2271304161,-2.0933010142
```

{63} [Fe(AG$_D$)$_3$]$^{2+}$ hs

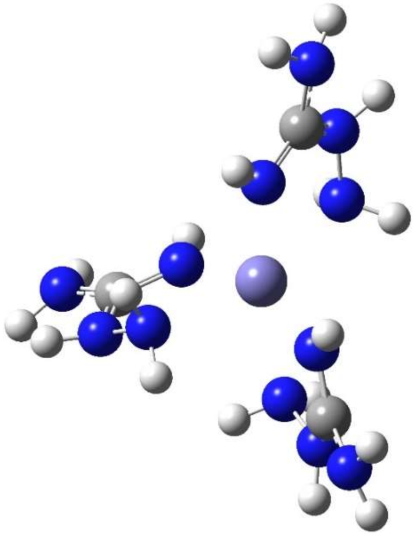

```
Charge =  2 Multiplicity = 5
 Fe,0,0.1688301073,0.0833211402,0.1745377172
 N,0,1.9439660888,-0.3232929334,1.6706842399
 H,0,2.2355503957,0.5445404419,2.1122073716
 H,0,1.6740367548,-0.9664449836,2.4067355954
 N,0,3.0149758051,-0.875698612,0.964118361
 H,0,3.9229634641,-0.8355053734,1.4067215786
 C,0,2.9754230509,-0.8071474814,-0.4020850275
 N,0,4.14725347,-1.0843038623,-1.0302371331
 H,0,4.8599889077,-1.5755592004,-0.5122538237
 H,0,4.0785194496,-1.3402490393,-2.0026861869
 N,0,1.8651043678,-0.4864151708,-0.9958194083
 H,0,1.9550693336,-0.5225144696,-2.0037252353
 N,0,-1.5107461084,-0.4372839021,-1.3921136702
 H,0,-1.1071407977,-0.7585756791,-2.2678575164
 H,0,-2.0576677417,0.3946202505,-1.5845498607
 N,0,-2.3482475058,-1.4188278508,-0.8620870851
 C,0,-1.8515450685,-2.1521788304,0.1817900096
 H,0,-2.9697646494,-1.8883179919,-1.5067891095
 N,0,-2.5513113669,-3.2746772685,0.4863425487
 H,0,-3.4976352458,-3.3473017089,0.1448939449
 H,0,-2.3995178165,-3.6619434579,1.4042471143
 N,0,-0.7724157352,-1.7482972507,0.782911662
 H,0,-0.5278473133,-2.3384171732,1.5684460357
 N,0,-1.513667899,1.2299607855,1.4722672452
 H,0,-1.3693085486,1.200059916,2.4754402206
 H,0,-2.3861792119,0.7505506785,1.27064028
 N,0,-1.5962432275,2.5589187732,1.0633619849
 H,0,-2.4604189329,3.0537740124,1.2351966798
 C,0,-0.831930649,2.9361085013,-0.0028282884
 N,0,-1.1224844814,4.1511267925,-0.5349076038
 H,0,-1.6446459021,4.8029356121,0.0311080393
 H,0,-0.4062342681,4.5678435145,-1.108435449
 N,0,0.0907340739,2.1304690481,-0.4396694147
 H,0,0.6203072004,2.5338827734,-1.2018098162
```

{64} [Fe(AG$_D$)$_3$(H$_2$O)]$^{2+}$ hs

```
Charge =  2 Multiplicity = 5
 Fe,0,0.1308619838,0.0128075956,-0.2633255182
 N,0,1.5391763585,-0.3801270703,1.5658955836
 H,0,1.835184356,0.5101222909,1.9582211074
 H,0,1.0715579525,-0.8990459393,2.3017750852
 N,0,2.6492594114,-1.1075953608,1.1370585957
 H,0,3.4565230728,-1.125912027,1.7447665717
 C,0,2.8560611258,-1.2223594348,-0.2090534006
```

```
N,0,4.064578779,-1.7322122401,-0.5660317856
H,0,4.5705918801,-2.261732681,0.1280497827
H,0,4.1256935289,-2.1007148911,-1.5022205685
N,0,1.9282745631,-0.8393018266,-1.0349700379
H,0,2.1746132109,-1.0421812324,-1.9961751762
N,0,-1.2357016763,-0.4792871354,-2.0864379542
H,0,-0.705139798,-0.9021377447,-2.8434170434
H,0,-1.6642363144,0.3677364109,-2.4446485976
N,0,-2.2470628774,-1.3481075107,-1.6693402098
C,0,-2.0303766186,-2.038596523,-0.5064353288
H,0,-2.7718413359,-1.8188265073,-2.3945796616
N,0,-2.8883237331,-3.0648815126,-0.2741817554
H,0,-3.7628351125,-3.0707832412,-0.7778202578
H,0,-2.9428371071,-3.3909677477,0.677665774
N,0,-1.04732497,-1.6886314524,0.2666050153
H,0,-1.0220216802,-2.2301712968,1.1221901194
N,0,-1.7058856732,1.3044752045,0.6217346399
H,0,-1.6639687375,1.2146853868,1.6344781435
H,0,-2.6078004412,0.9761309269,0.296493449
N,0,-1.5460534725,2.6415614954,0.2646358696
H,0,-2.0401934709,3.3294563926,0.8182490749
C,0,-0.316206462,3.0004538664,-0.2143039436
N,0,-0.1170835905,4.3301529644,-0.3791974005
H,0,-0.7089756056,4.9735793006,0.1235558358
H,0,0.8335373693,4.6316090642,-0.5202276799
N,0,0.5319423837,2.0721320615,-0.5525660799
H,0,1.3795275485,2.4610753718,-0.9469359515
O,0,-0.3194968029,1.9055934297,3.3302714153
H,0,0.149905812,1.1912561843,3.7705423749
H,0,0.3069431433,2.2213194291,2.6720169131
```

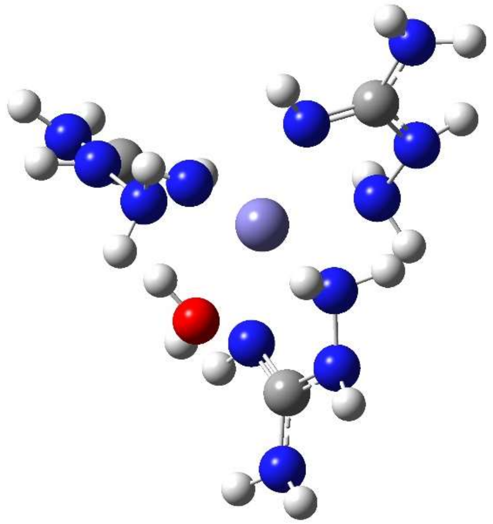

{65} $[Fe(AG_D)_2(H_2O)_2]^{2+}$ hs (cis)

```
Charge =  2 Multiplicity = 5
 N,0,0.2649728259,0.3240612352,1.9493496042
 H,0,-0.6651576489,0.5574754335,2.276528331
 H,0,0.6572592634,-0.3732169818,2.5759360656
 N,0,1.7723789021,0.8581137424,-0.1525869881
 H,0,2.3996365741,1.1292897755,-0.8998483137
 C,0,1.883520593,1.6461141138,0.8791423424
 N,0,1.0515472652,1.4713614985,1.9479098181
 H,0,1.3466555913,1.8430997365,2.8409151727
 N,0,2.7244618201,2.70403946,0.9658988603
 H,0,3.4853827229,2.7402714994,0.3074745715
 H,0,2.9095639605,3.099653921,1.87483601
 N,0,-1.245414205,1.4434354793,-0.9046340269
 H,0,-0.7865167752,2.2936522301,-0.5969071923
 H,0,-1.3836989729,1.5062222861,-1.9097202469
 N,0,-2.4761413972,1.3317558472,-0.2557018456
 C,0,-2.8329693208,0.0820828879,0.1760289904
 N,0,-1.9438013642,-0.8644183628,0.1971328031
 N,0,-4.1309946495,-0.0596886434,0.542322018
 H,0,-4.3511472965,-0.8577988764,1.1166273583
 H,0,-4.6673218392,0.7718825471,0.737147601
 H,0,-2.3136753683,-1.7242544927,0.5828934773
 H,0,-3.2212835445,1.9335442922,-0.5808556839
 O,0,0.1927829494,-1.1681279766,-2.4641816682
 H,0,0.3796535937,-0.4793391469,-3.1094298343
 H,0,0.8892379421,-1.8208672441,-2.5884352695
 O,0,0.983104556,-2.5150239526,0.167820625
 H,0,1.4859421084,-2.504665229,0.9880469405
 H,0,1.6136375183,-2.7877716542,-0.5060312495
 Fe,0,0.0911111957,-0.4313484251,-0.3276682704
```

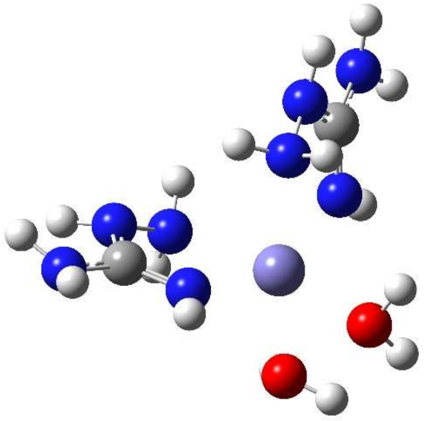

{66} $[Fe(AG_D)_2(H_2O)_2]^{2+}$ hs (mirror image)

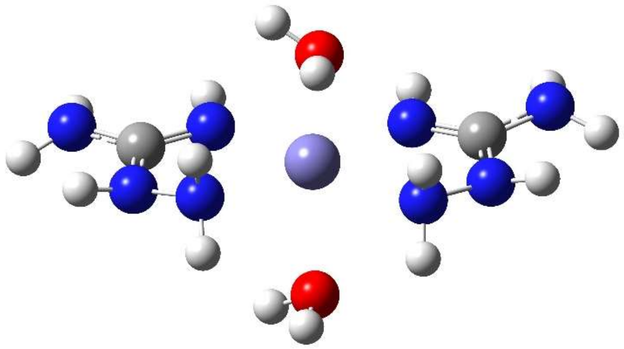

```
Charge =  2 Multiplicity = 5
 N,0,-1.906748264,-1.3314686487,0.2097901518
 H,0,-1.964087327,-1.9582589293,-0.5881129456
 H,0,-1.8774678139,-1.8986972479,1.0501538634
 N,0,-1.6980203342,1.3208210817,-0.1056343755
 H,0,-1.7249413219,2.3147355045,-0.2959651351
 C,0,-2.8915755798,0.8060987484,-0.0479545368
 N,0,-3.0411808057,-0.5192181049,0.2530894941
 H,0,-3.9266657266,-0.9555725057,0.0376038522
 N,0,-4.0476867911,1.4797218665,-0.273724999
 H,0,-3.9990338621,2.48405688,-0.2047122197
 H,0,-4.9055770151,1.0715908757,0.0662540613
 N,0,1.8737853438,-1.3981639298,0.0001855787
 H,0,1.8443229147,-2.0789374572,0.7511643469
 H,0,1.9206975093,-1.9058664587,-0.8792477459
 N,0,3.0150182573,-0.6106211394,0.1572643748
 C,0,2.893239392,0.7314731821,-0.0702730656
 N,0,1.7092917837,1.2600720828,-0.1769362806
 N,0,4.064307267,1.4089555574,-0.179346912
 H,0,4.0135522968,2.4057381054,-0.0383240285
 H,0,4.8983725454,0.971238208,0.1823606896
 H,0,1.756898704,2.2648697492,-0.2924227272
 H,0,3.9039606752,-1.0432396263,-0.0531519509
 O,0,-0.2222890007,-0.6336305798,-2.2167369665
 H,0,0.550570439,-0.342328669,-2.7120260776
 H,0,-0.2000489191,-1.5944522867,-2.2807590666
 O,0,0.0160501814,-0.3069959687,2.3142696525
 H,0,0.7797651481,0.1420196072,2.6918116942
 H,0,0.1621175911,-1.2393433598,2.5064619395
 Fe,0,-0.0216022878,0.0200634632,0.008146334
```

{67} $[Fe(AG_D)_2(H_2O)_2]^{2+}$ hs (trans)

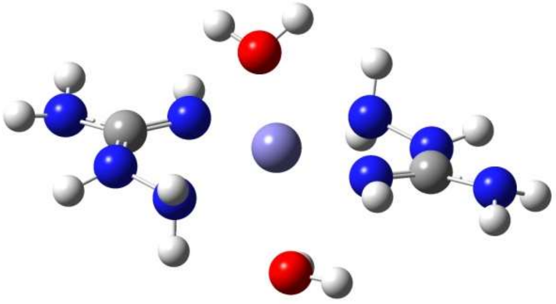

```
Charge =  2 Multiplicity = 5
 N,0,1.7805909594,-1.4167672813,0.6209337193
 H,0,1.7570271006,-1.457561967,1.6361384082
 H,0,1.7078368165,-2.3636253608,0.265577661
 N,0,1.8313134015,1.0273325734,-0.4376070748
 H,0,1.9565823726,1.9744925921,-0.7717718667
 C,0,2.9713911582,0.4370598922,-0.2202832522
 N,0,2.9882179063,-0.8664280399,0.1905069194
 H,0,3.8305309518,-1.1980347405,0.6413693178
 N,0,4.1861163389,1.0205390615,-0.3546090646
 H,0,4.2260077633,1.8630391387,-0.9056022403
 H,0,4.999527381,0.4266351775,-0.4084719703
 N,0,-1.541852954,1.6629841338,-0.5169168312
 H,0,-1.2895142334,2.487197545,0.0184281227
 H,0,-1.6027171001,1.9347772257,-1.4946961227
 N,0,-2.7838779044,1.205193744,-0.0732125662
 C,0,-2.9378113768,-0.1409811235,0.1086289613
 N,0,-1.8900137168,-0.912206081,0.1106319139
 N,0,-4.2167940756,-0.564885885,0.26373119
 H,0,-4.3364450693,-1.4702854493,0.6899662993
 H,0,-4.9164266087,0.1192136632,0.5087106967
 H,0,-2.1325120347,-1.8790583146,0.2862975539
 H,0,-3.5965331724,1.7376698677,-0.3520089994
 O,0,0.2878737054,-1.1205320588,-2.2156973831
 H,0,-0.394601104,-0.9269771867,-2.8660359218
 H,0,1.1079948394,-0.8200539098,-2.6211340639
 O,0,-0.2594769297,0.5057921291,2.3337972475
 H,0,-1.1772933396,0.229614091,2.4177841156
```

```
 H,0,-0.2951861998,1.4636268983,2.4179481724
 Fe,0,0.0288431245,-0.0313113347,-0.1725099417
```

{68} $[Fe(AG_A)_2(H_2O)_2]^{2+}$ hs (4-coord.)

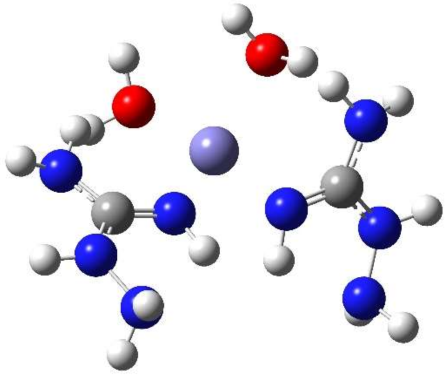

```
Charge =  2 Multiplicity = 5
 N,0,-3.7376336827,2.332824031,-0.9486163088
 H,0,-3.9619669898,3.1242348398,-0.3548934583
 H,0,-4.3373933254,2.3682071671,-1.7641532476
 N,0,-3.9702675449,1.1490007653,-0.2648408215
 C,0,-2.9212280307,0.362994227,0.0946343092
 N,0,-1.6912720045,0.6007995158,-0.2769323639
 N,0,-3.2295500452,-0.6894539469,0.8916578881
 H,0,-4.1845140338,-1.0106640715,0.9078424581
 H,0,-2.5200122111,-1.398632915,0.9965809395
 H,0,-1.662464355,1.4627548435,-0.8118580751
 H,0,-4.8553224363,1.0145225491,0.2049089067
 N,0,3.6546224951,2.4849067488,0.6397359247
 H,0,3.8202133725,3.2081530724,-0.0520648487
 H,0,4.2597881482,2.6590638664,1.4328200126
 N,0,3.9544751263,1.2406748806,0.1032284546
 C,0,2.9416105192,0.3775712232,-0.1788571981
 N,0,1.704417367,0.5898168567,0.1794811975
 N,0,3.2963136859,-0.7242413993,-0.8861471923
 H,0,4.2671589195,-0.9956857849,-0.8838056145
 H,0,2.6332090655,-1.4823960424,-0.884887053
 H,0,1.6415169754,1.4915354086,0.6415717711
 H,0,4.8328640145,1.1155900282,-0.3821948344
 Fe,0,0.002525954,-0.6210151086,-0.0473824794
 O,0,0.1649048295,-2.0448918004,-1.6867745793
 H,0,0.8277615754,-1.8430804001,-2.3549238793
 H,0,0.3406645518,-2.9530829495,-1.4207797044
 O,0,-0.4098666938,-1.9914109705,1.6197395989
 H,0,-0.4305511099,-1.568051185,2.4843014172
 H,0,0.2083258635,-2.7238094494,1.7114637803
```

{69} $[Fe(AG_B)_2(H_2O)_2]^{2+}$ hs (4-coord.)

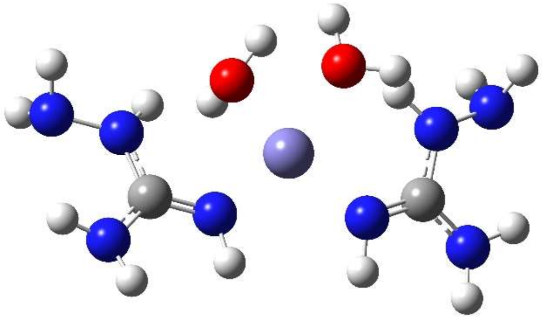

```
Charge =  2 Multiplicity = 5
 N,0,4.8255427679,-0.3933693831,-0.3921205754
 H,0,5.1585761606,-0.9426843447,0.3910558186
 H,0,5.2374090705,0.5316608754,-0.3289083279
 N,0,1.3736099089,-0.4029109937,-1.3306944483
 H,0,0.9185817979,-0.6478234243,-2.2006998608
 C,0,2.6728484748,-0.5881854086,-1.4059567966
 N,0,3.4433131437,-0.2996736923,-0.3282197377
 H,0,3.0020476803,0.1744835032,0.4481565853
 N,0,-3.4683402984,2.7994368502,1.7758660361
 H,0,-3.333560663,3.7640155078,1.4923805038
 H,0,-3.4125751309,2.7569860059,2.7864880183
 N,0,-2.4694319676,2.00353258,1.2326560883
 C,0,-2.7567511023,1.0848220937,0.2777935004
 N,0,-1.7739233829,0.4001708956,-0.2649642336
 H,0,-2.1227358518,-0.2544475096,-0.9536928904
 H,0,-1.4971506499,2.2568334165,1.3539590648
 N,0,-4.0493680155,0.9473565961,-0.0704645332
 H,0,-4.3225970796,0.1240449476,-0.5800193393
 H,0,-4.736782614,1.4144172394,0.5000820549
 N,0,3.3056621225,-1.0363568416,-2.5060354156
 H,0,4.2768419352,-1.2901554947,-2.4143713216
 H,0,2.756756323,-1.4743512316,-3.2263611491
 O,0,0.8293846821,2.3566806635,0.9442937038
 H,0,0.9428475888,3.040625052,0.2764637535
 H,0,1.6533151779,2.3482928755,1.4426791344
```

```
O,0,0.815445927,-0.6715430942,2.0727534301
H,0,1.2246300546,-0.0750317853,2.7083500858
H,0,1.4859044103,-1.3387957429,1.8929365108
Fe,0,0.2430725298,0.3894768443,0.2121543402
```

{70} $[Fe(AG_C)_2(H_2O)_2]^{2+}$ hs (4-coord.)

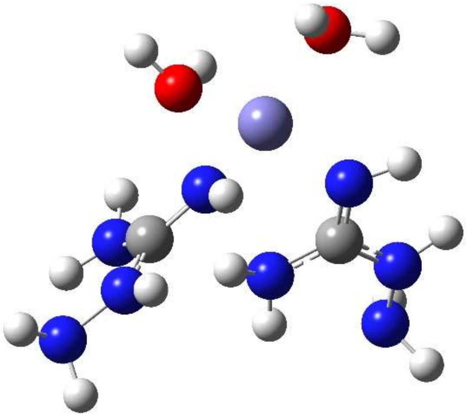

```
Charge =  2 Multiplicity = 5
N,0,-3.977878367,-0.8707713226,-1.961472098
H,0,-4.7222171906,-0.3860629694,-2.4485718016
H,0,-4.345578211,-1.7513426048,-1.6166085876
N,0,-3.5474072112,-0.0889733519,-0.8978483275
C,0,-2.2251907866,0.1589806916,-0.7026564166
N,0,-1.7949703429,0.8451597138,0.3303039071
N,0,-1.3761533228,-0.3421151316,-1.6205651623
H,0,-1.7878651484,-0.7145210982,-2.4620442031
H,0,-0.4457092117,0.0424076761,-1.6712146755
H,0,-2.562374699,1.161740298,0.9112132916
H,0,-4.1737855657,0.0928071363,-0.1256217611
N,0,3.1945338024,2.0231645312,-3.4404989727
H,0,4.1619970574,2.3267512673,-3.433862932
H,0,2.7543377797,2.389389817,-4.277198324
N,0,2.5462598066,2.5251121193,-2.322078225
C,0,1.9773249475,1.7089709534,-1.4043100967
N,0,1.4256597658,2.1791205447,-0.3088043387
N,0,1.9615313117,0.3861698923,-1.6822590623
H,0,2.5703481763,0.0801141561,-2.4259159073
H,0,1.8172039618,-0.2384067224,-0.9023689908
H,0,1.4889386511,3.1896116088,-0.2683659848
H,0,2.5359168287,3.519734009,-2.1426516036
O,0,0.0289734105,2.2086160801,2.8830416897
H,0,-0.8280236472,2.6248876151,3.0200781454
H,0,0.6778349932,2.9073577858,3.0132983063
O,0,1.0023090606,-0.9182519494,1.0919581376
H,0,1.8417505164,-0.9980875106,1.5574145646
H,0,0.4223002236,-1.5680349822,1.5025811774
Fe,0,0.1840224105,1.1363137472,0.9912752513
```

{71} $[Fe(AG_D)(H_2O)_3]^{2+}$ hs (5-coord., sq. pyr.)

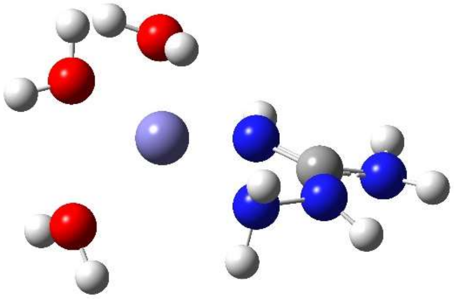

```
Charge =  2 Multiplicity = 5
N,0,-1.3961370797,1.5921445607,0.831060482
H,0,-1.4247938023,1.8963762761,1.7984946267
H,0,-1.1029624907,2.3834610015,0.2641812299
N,0,-2.6721675451,1.1803544444,0.44785215
C,0,-2.7708438035,0.0223240341,-0.2663189569
N,0,-1.7356872751,-0.766458709,-0.3478140759
N,0,-3.9680613507,-0.2068263484,-0.8499873684
H,0,-4.1634094983,-1.1532879068,-1.1352766374
H,0,-4.7641058529,0.3293818129,-0.5403650998
H,0,-1.9348246944,-1.6083610034,-0.8725182716
H,0,-3.3586820563,1.9027366309,0.2769150065
Fe,0,0.0579639345,-0.1953200338,0.5450953118
O,0,1.4454249909,-1.9041580395,0.0487822055
H,0,2.372501239,-1.6449558871,0.0529463367
H,0,1.3895276452,-2.6434228579,0.6624315221
O,0,1.7051294471,1.0219486364,-0.309517706
H,0,2.1689284785,0.5592153071,-1.0149721824
H,0,1.4306094472,1.8604894963,-0.6938466558
O,0,0.1809526791,-0.7989986831,2.6716069739
H,0,1.0777889408,-1.0323366104,2.9341090772
H,0,-0.0748541434,-0.0830798011,3.2633205018
```

{72} $[Fe(AG_D)_2(H_2O)]^{2+}$ hs (5-coord.)

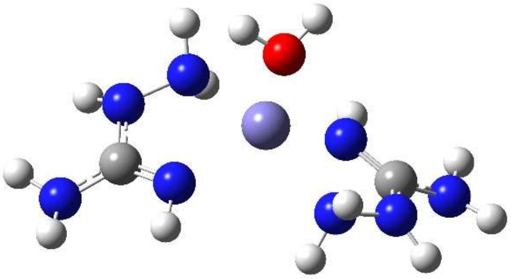

```
Charge =  2 Multiplicity = 5
 N,0,1.5580755751,-1.5191526816,0.3809740683
 H,0,1.6918904286,-2.2293189466,1.0926825439
 H,0,1.21116447,-1.9785804895,-0.4568380282
 N,0,1.7262350058,1.1341179206,0.3345530858
 H,0,1.8577838084,2.1307457833,0.2165133607
 C,0,2.7811398511,0.4554805446,-0.0200853498
 N,0,2.7813438005,-0.9026029789,0.1200081816
 H,0,3.4637522244,-1.4313345014,-0.4060779444
 N,0,3.9097833084,0.9894654703,-0.5432025862
 H,0,4.0542645213,1.9753060877,-0.3925362086
 H,0,4.7468808589,0.426332658,-0.5482174268
 N,0,-1.4606266508,1.8672261904,0.8078102936
 H,0,-1.6812655339,2.3385587362,1.6783188822
 H,0,-1.0580056748,2.5565039232,0.1782876792
 N,0,-2.6395543121,1.3555036084,0.2621997614
 C,0,-2.5866814962,0.1024308112,-0.2813251708
 N,0,-1.5569932458,-0.6573291853,-0.0357290353
 N,0,-3.6411907026,-0.2405150482,-1.0566308173
 H,0,-3.7729846477,-1.2225400687,-1.2396242659
 H,0,-4.4851015702,0.3079478251,-0.9901303717
 H,0,-1.6448030603,-1.5689611461,-0.466839772
 H,0,-3.2734165945,2.0209440174,-0.1592171885
 Fe,0,0.0486281085,0.1153767567,1.0803846198
 O,0,0.0754657643,-0.4561663602,3.1762022635
 H,0,0.6296594166,0.1147236027,3.7189531708
 H,0,-0.7904666532,-0.4386835292,3.5976562548
```

{73} $[Fe(AG_D)(H_2O)_5]^{2+}$ hs

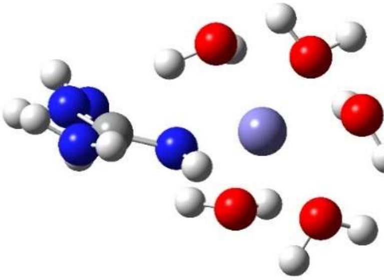

```
Charge =  2 Multiplicity = 5
 N,0,-1.9698709183,1.8562388464,-0.1293772141
 H,0,-2.4121345913,2.6565224371,0.3089773421
 H,0,-1.9198486187,2.0564775006,-1.1270194348
 N,0,-2.8144443403,0.7637483719,0.0926607778
 C,0,-2.374790096,-0.5148043183,-0.0912273355
 N,0,-1.1195697358,-0.838113263,-0.223833052
 N,0,-3.3855962263,-1.4331910565,-0.1626152911
 H,0,-3.1211206017,-2.3827585748,0.0504793217
 H,0,-4.2812922463,-1.1660401479,0.2186516762
 H,0,-1.0572636965,-1.8448274694,-0.3387617043
 H,0,-3.788645404,0.9130798206,-0.1364206946
 O,0,1.6348602933,-1.9390875075,-1.1681946127
 H,0,1.2183370074,-1.9424957242,-2.0359944859
 H,0,2.5749446482,-1.8490929414,-1.353275239
 O,0,0.4080625248,1.680915374,1.3549265042
 H,0,-0.4119692317,1.8834253775,0.8505461523
 H,0,1.0318172879,2.3774665797,1.1306837479
 Fe,0,0.8956910698,-0.1378362181,0.0844618399
 O,0,1.1321652697,-1.4178918621,1.8945529262
 H,0,1.9794502177,-1.2271719794,2.3111445373
 H,0,0.4759892465,-1.2119613738,2.5686661813
 O,0,3.1440950376,0.4306227563,0.4277615503
 H,0,3.2023776388,1.3904993586,0.3846556148
 H,0,3.6771228167,0.1245030315,-0.3129477562
 O,0,1.1174425685,1.144799033,-1.758132655
 H,0,0.4375034924,1.8226376654,-1.8331201325
 H,0,1.9486025874,1.629332284,-1.7189215639
```

{74} $[Fe(AG_B)(H_2O)_5]^{2+}$ hs

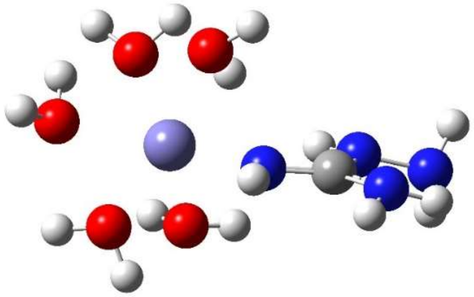

```
Charge =  2 Multiplicity = 5
 N,0,-3.7402625443,-0.9628589854,-0.0887740409
 H,0,-4.0226459167,-1.5625044383,0.676982569
 H,0,-3.7871938509,-1.5015589602,-0.9470778885
 N,0,-2.4384642152,-0.5281545786,0.1262984783
 C,0,-2.1216892865,0.7900993737,0.015187995
 N,0,-0.8619204035,1.1464524381,-0.0052673994
 H,0,-0.7737190352,2.1552953097,-0.0285237278
 H,0,-1.6727941746,-1.1835450165,0.0510079631
 N,0,-3.1589766023,1.657471528,-0.0004774971
 H,0,-2.9731433153,2.5916190617,-0.3257546899
 H,0,-4.067730475,1.2665208892,-0.1980337086
 O,0,0.5239608854,-1.6977981678,1.475629067
 H,0,-0.2044962649,-1.4577163584,2.0576722324
 H,0,0.1800630838,-2.4322068672,0.9564142601
 O,0,1.7839457747,1.7095673111,-1.32327079
 H,0,1.358906496,1.7267486318,-2.1870166062
 H,0,2.7186215202,1.5808864773,-1.5148965545
 Fe,0,0.9569381854,0.0432978401,-0.0117290989
 O,0,3.110569367,-0.8827025898,-0.0150276478
 H,0,3.7259038496,-0.1877921005,0.2387085702
 H,0,3.1733870068,-1.53007073,0.6941869429
 O,0,0.5995135293,-1.3602733211,-1.7384313011
 H,0,1.2641606857,-2.0565680216,-1.7603968364
 H,0,-0.2448975872,-1.8213924866,-1.7127763165
 O,0,1.6709608295,1.2601429074,1.8244707743
 H,0,2.4551474012,0.8245043076,2.17435915
 H,0,1.0310890572,1.2203965466,2.5427501013
```

{75} $[Fe(AG_D)(H_2O)_4]^{2+}$ hs

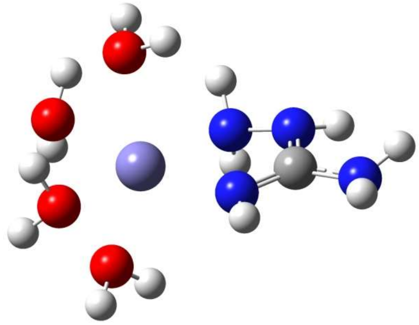

```
Charge =  2 Multiplicity = 5
 N,0,0.9072139452,1.128279053,-1.1354467092
 H,0,0.9277812651,0.8971866866,-2.1251782678
 H,0,0.6689915808,2.1104082008,-1.0480569789
 N,0,2.1635674532,0.9166995337,-0.5678937445
 C,0,2.3258105292,-0.2231751812,0.1685294436
 N,0,1.2778626116,-0.9159396808,0.5103173821
 N,0,3.6035786156,-0.5468646969,0.4734301092
 H,0,3.7366601566,-1.2055462371,1.2243764754
 H,0,4.3093114567,0.1696444733,0.3960501307
 H,0,1.5142178038,-1.7023244542,1.1018039192
 H,0,2.9636224248,1.2492015854,-1.0895743966
 O,0,-0.9030203373,1.3849187389,1.7718114111
 H,0,-0.0852134439,1.3999907271,2.2791880631
 H,0,-0.9572899707,2.2625758665,1.3806468747
 O,0,-1.2592218694,-1.4673525187,-1.7911261005
 H,0,-1.3930142024,-2.3875619319,-1.5427586636
 H,0,-0.5829972625,-1.495768449,-2.4754115161
 Fe,0,-0.642477735,-0.1872550305,0.0181047002
 O,0,-2.0258028153,-1.5746785831,1.1599778591
 H,0,-2.4683788407,-1.1135783603,1.8794861845
 H,0,-2.7370034699,-1.8893331656,0.5927030123
 O,0,-2.4484677649,1.0854401988,-0.7297897304
 H,0,-2.5285800495,0.9426735383,-1.6783148048
 H,0,-2.2411364347,2.0213059608,-0.6407434537
```

{76} $[Fe(AG_A)(H_2O)_4]^{2+}$ hs (5-coord.)

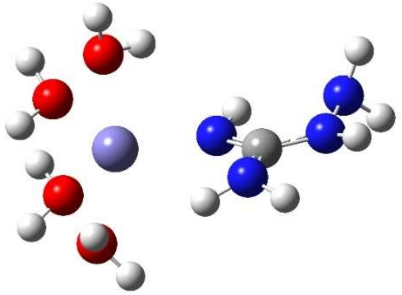

```
Charge =  2 Multiplicity = 5
 N,0,-3.7006152586,-1.0054772814,0.3811498782
 H,0,-4.3841044925,-1.3383045957,-0.2880313257
 H,0,-4.1261636659,-1.0129968752,1.3019973852
 N,0,-3.3266465891,0.2872944665,0.0453911196
 C,0,-2.0070391864,0.6018115409,-0.0390854907
 N,0,-1.0613183442,-0.2949529332,0.0279331909
 N,0,-1.7286717752,1.9235731579,-0.1736297721
 H,0,-2.4711970534,2.5237224935,-0.4976903397
 H,0,-0.8161304078,2.1425178228,-0.5398370622
 H,0,-1.4791504382,-1.2044242144,0.2010452389
 H,0,-3.9934513561,1.0389559101,0.1574327413
 O,0,1.1483445409,-2.0119137169,1.4322266715
 H,0,1.1569036639,-1.7022943898,2.3432141556
 H,0,0.3892802091,-2.601301154,1.3804443796
 O,0,1.5282466093,1.4780568679,-1.4352686795
 H,0,1.5141632162,2.3353834851,-0.9974836206
 H,0,0.9192461212,1.5700650008,-2.1748176882
 Fe,0,1.0289516476,-0.2260924416,-0.0169405158
 O,0,2.2739946411,-1.4775855878,-1.3318852513
 H,0,2.9675429385,-0.9870529565,-1.7849510179
 H,0,2.7259315726,-2.1867938142,-0.863480157
 O,0,2.1124016581,0.871064836,1.5604475419
 H,0,2.8107188628,1.4641180972,1.2659035526
 H,0,2.520752886,0.3034242821,2.2219030655
```

{77} $[Fe(AG_D)_2]^{2+}$ hs (4-coord.)

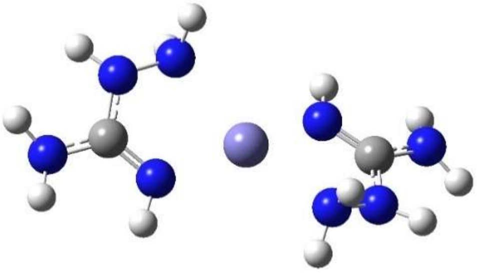

```
Charge =  2 Multiplicity = 5
 N,0,1.7385480501,-1.5019145917,0.4851957409
 H,0,1.839237017,-1.7426376366,1.4681324521
 H,0,1.5794979143,-2.3622167711,-0.0283177931
 N,0,1.7555354407,1.0790898284,-0.114244331
 H,0,1.8618145628,2.0687745864,-0.2966989856
 C,0,2.8950553125,0.446882311,-0.1539829391
 N,0,2.9093052044,-0.9078796765,0.0143439513
 H,0,3.7824626274,-1.3415194858,0.2821180461
 N,0,4.1003359543,1.0282619457,-0.3456857523
 H,0,4.0983205612,1.9510465708,-0.7503914697
 H,0,4.8808489426,0.442799082,-0.6020526629
 N,0,-1.6371049897,1.6536802897,0.4848097957
 H,0,-1.5465023498,2.1012926481,1.3905032256
 H,0,-1.566935384,2.375746805,-0.2270572777
 N,0,-2.8836887369,1.0331591715,0.4138515646
 C,0,-2.9438469108,-0.2057791733,-0.1530818842
 N,0,-1.8389876238,-0.8613243175,-0.3874992955
 N,0,-4.1822314909,-0.6607657111,-0.4414211798
 H,0,-4.2793015285,-1.6495425421,-0.6087239423
 H,0,-4.9755777468,-0.2079065055,-0.0136352452
 H,0,-2.0123629022,-1.7846791482,-0.7623829963
 H,0,-3.694259327,1.6311779315,0.3242875309
 Fe,0,-0.0173662569,-0.0267157105,0.1541737677
```

{78} $[Fe(H_2O)_6]^{3+}$ hs

```
Charge =  3 Multiplicity = 6
 Fe,0,0.5112394738,-0.4336466633,-0.0261871528
 O,0,0.5817499339,1.7043221437,0.0661055836
 H,0,1.3648090614,2.0368426561,-0.3908720969
 O,0,-1.6523268471,-0.3514392415,0.1371132801
 H,0,-2.0160528412,-1.240148818,0.0376499341
 O,0,2.6679911333,-0.5217215281,-0.1967853412
 H,0,3.0349572659,-1.0160951372,0.5467803002
 O,0,0.6545197795,-0.5445881548,2.1265779498
 H,0,0.1089473262,-1.2689640468,2.4584906012
 O,0,0.4397544879,-2.5820025749,-0.1032840185
 H,0,0.8543789144,-2.8965253035,-0.9169029103
 O,0,0.3486543548,-0.3633628922,-2.1747142003
 H,0,-0.5781171275,-0.2387372685,-2.4161515805
 H,0,0.6621384966,2.0123225949,0.9778401591
 H,0,0.602004061,-1.2121809018,-2.5595397991
 H,0,3.0331460984,0.3686484058,-0.1201326225
 H,0,0.9645156382,-2.9573666144,0.6156193404
 H,0,0.2890083411,0.2556791034,2.5238177079
 H,0,-2.0306775506,0.1652962409,-0.5854271343
```

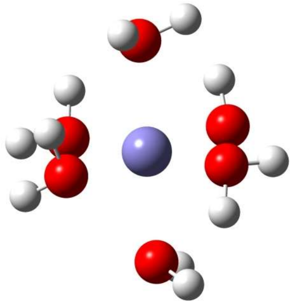

{79} $[Fe(H_2O)_6]^{3+}$ ls

```
Charge =  3 Multiplicity = 2
 Fe,0,0.4907155434,-0.5442860894,-0.0160568095
 O,0,0.3253516386,1.4346214582,-0.1868944453
 H,0,1.1568207469,1.8595791258,-0.4408163305
 O,0,-1.4693891134,-0.6315906813,-0.1941343653
 H,0,-1.8729836229,-1.1335614039,0.5278590085
 O,0,2.4698590922,-0.3662869223,0.1923735526
 H,0,2.908986032,-1.2275057021,0.2311714308
 O,0,0.3345132992,-0.6099146657,1.9938454078
 H,0,1.1104504811,-1.0417897225,2.377415986
 O,0,0.6037235252,-2.5430008192,0.1267867779
 H,0,0.5562639511,-2.9638329693,-0.7428628154
 O,0,0.5421432943,-0.5738686787,-2.0095475323
 H,0,0.0476172049,0.1683242864,-2.384760626
 H,0,0.0490580101,1.8552100526,0.6393247428
 H,0,1.4412040207,-0.4998702538,-2.3588829541
 H,0,2.8612012684,0.1003613847,-0.559772846
 H,0,-0.1458403897,-2.8897269973,0.6320021949
 H,0,0.3223377835,0.2844505019,2.3625230716
 H,0,-1.8913947658,0.2390210959,-0.1895724485
```

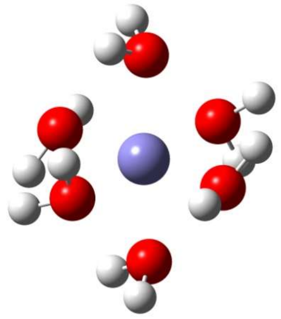

{80} $[Fe(H_2O)_6]^{2+}$ hs

```
Charge =  2 Multiplicity = 5
 Fe,0,0.5480206471,-0.5020279532,0.0855502379
 O,0,0.3470246467,1.7255976515,0.0327313423
 H,0,0.9289275093,2.1507977846,-0.6052707506
 O,0,-1.6993790635,-0.6609487218,0.1206184435
 H,0,-2.0173083273,-1.1437621679,-0.6495485265
 O,0,2.779923116,-0.4407520038,-0.0773033764
 H,0,3.0363704432,-0.8733213297,-0.8987709475
 O,0,0.6353152455,-0.3947256572,2.3288518721
 H,0,0.5508113424,-1.2686381341,2.7236287017
 O,0,0.6494501538,-2.7422039122,0.2151951916
 H,0,0.1392016886,-3.1721793388,-0.4784918149
```

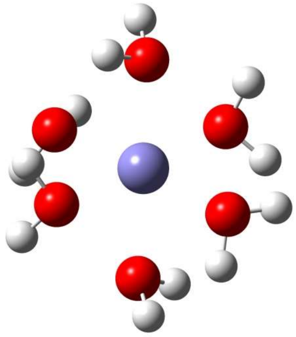

O,0,0.463562215,-0.6595754576,-2.1730760917
H,0,0.4925555661,0.1997561645,-2.6055694359
H,0,0.569146741,2.1290921701,0.8779428366
H,0,1.2426326207,-1.1218813624,-2.4985356582
H,0,3.0992751985,0.4631715396,-0.1679978709
H,0,0.2678192548,-3.0633992114,1.0385487477
H,0,-0.0877769747,0.1170199871,2.7048077024
H,0,-2.0940630233,0.2136789527,0.0400683968

{81} AGH^+

Charge = 1 Multiplicity = 1
C,0,-0.5010286928,0.0103715891,0.0041964808
N,0,0.6595667723,-0.6491621382,-0.0531533786
N,0,1.8562754498,0.0468278718,-0.1014158253
N,0,-0.5075005806,1.3327690572,0.0446957836
N,0,-1.6384791073,-0.6837524445,-0.0009224011
H,0,-2.5055459869,-0.208278755,0.1898411662
H,0,-1.6091955947,-1.6812730878,0.1392147922
H,0,0.6443556166,-1.6593787368,-0.1034638027
H,0,2.3442752974,-0.1900499848,-0.9581074148
H,0,0.3719683465,1.8220891832,-0.0103350135
H,0,2.4337630924,-0.2336493311,0.6831292744
H,0,-1.3767643027,1.8395189069,0.0686572287

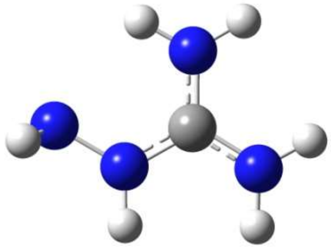

{82} $(AGH^+)_2$

Charge = 2 Multiplicity = 1
N,0,-1.218931,1.519416,0.375765
H,0,-1.086277,1.841759,1.328854
H,0,-1.071501,2.309877,-0.243675
N,0,-1.829234,-1.056084,-0.278577
H,0,-2.052223,-2.010715,-0.509097
C,0,-2.803083,-0.177424,-0.107891
N,0,-2.528001,1.085404,0.230525
H,0,-3.294371,1.731824,0.36387
N,0,-4.076288,-0.527155,-0.300828
H,0,-4.307904,-1.505976,-0.359579
H,0,-4.809637,0.105152,-0.021409
N,0,1.21907,-1.519581,0.375488
H,0,1.086327,-1.842034,1.328526
H,0,1.071823,-2.309989,-0.244064
N,0,2.528099,-1.08538,0.23049
C,0,2.803049,0.177468,-0.107958
N,0,1.829076,1.055918,-0.27906
N,0,4.076237,0.527415,-0.300538
H,0,4.307731,1.506246,-0.359537
H,0,4.809649,-0.104789,-0.021056
H,0,2.05197,2.010623,-0.509363
H,0,3.294549,-1.731661,0.36406
H,0,0.867011,0.800996,-0.097865
H,0,-0.867142,-0.801247,-0.097425

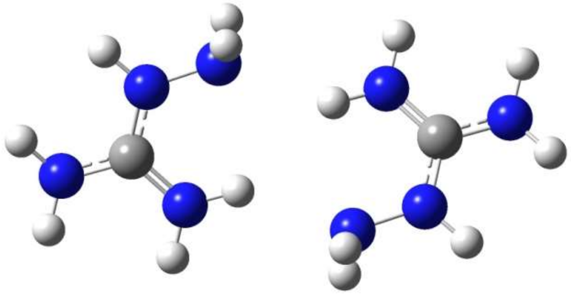

{83} $(AGH^+)_3$

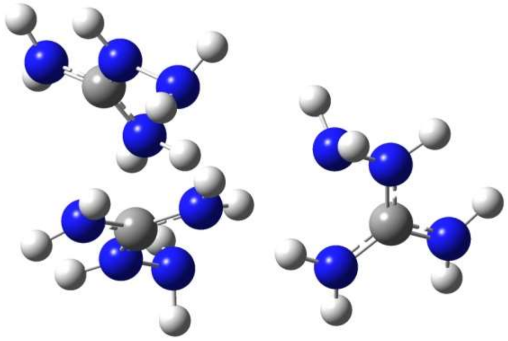

```
Charge =  3 Multiplicity = 1
 N,0,0.5174353851,1.9857169338,1.4954283874
 H,0,-0.0721315747,1.3322066494,2.0008711019
 H,0,1.1924727868,2.3654260111,2.1523704717
 N,0,-0.2730063605,3.0229534037,1.0234985235
 H,0,-0.7555257599,3.6286701419,1.6748190738
 C,0,-0.3006083149,3.3346176659,-0.2760372932
 N,0,-0.9892046587,4.4091647232,-0.6594824569
 H,0,-1.5789733751,4.8872695295,0.0027564819
 H,0,-1.160765954,4.5604399998,-1.6402895416
 N,0,0.3631893532,2.6082075884,-1.1607778103
 H,0,0.8323214267,1.7556205916,-0.883094277
 N,0,1.620697707,-0.2475058492,-1.496924916
 H,0,1.9203819068,0.0759193575,-2.4110430218
 H,0,0.7190682086,-0.7025990378,-1.6035773097
 N,0,2.5396495116,-1.1779307496,-1.0358692303
 C,0,3.1566896522,-1.0191347404,0.1387604149
 H,0,2.7551811701,-1.9975918844,-1.5883216303
 N,0,4.027930361,-1.94722573,0.5322099514
 H,0,4.3462082801,-2.6455878561,-0.1204902844
 H,0,4.5797350965,-1.7861458592,1.3591112059
 N,0,2.9072606995,0.0370947794,0.8986620726
 H,0,3.3304488138,0.0948463469,1.8108824281
 N,0,-0.7482654577,-1.2804328967,0.861053375
 H,0,-1.1213478564,-0.8925645448,1.7210348391
 H,0,0.2529555227,-1.4019555682,0.9725754461
 N,0,-1.335712514,-2.5113373093,0.6225580068
 H,0,-1.2277990001,-3.2762053183,1.2760985107
 C,0,-2.0062235511,-2.723562256,-0.5143218091
 N,0,-2.5150082334,-3.9310140575,-0.7519564973
 H,0,-2.5314161493,-4.6168875596,-0.0137626834
 H,0,-3.1399581196,-4.0593051867,-1.5314569248
 N,0,-2.1431529472,-1.7384702842,-1.3875221573
 H,0,-1.6699368868,-0.8675707739,-1.2007686467
 H,0,0.287099111,2.830444878,-2.1400598719
 H,0,2.1205515796,0.6397414804,0.6927343183
 H,0,-2.6119386187,-1.8917237984,-2.2648348572
```

{84} AG_A

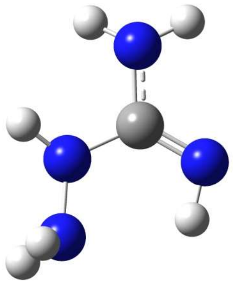

```
Charge = 0 Multiplicity = 1
 N,0,1.8464653399,0.0082114418,0.0387940031
 H,0,2.4963342272,-0.3649145358,-0.6414615986
 H,0,2.2224788455,-0.1735579737,0.9638701366
 N,0,0.6177828001,-0.6300332508,-0.116562949
 C,0,-0.526230913,0.1390255661,-0.0093279574
 N,0,-0.5691436878,1.4259681856,-0.0205693271
 N,0,-1.6711096016,-0.6152804174,0.1230851829
 H,0,-2.5085659506,-0.111951078,-0.1244106458
 H,0,-1.6367674705,-1.5387667349,-0.2832872472
 H,0,0.3922483982,1.7578824647,-0.0533177263
 H,0,0.5471860127,-1.5721186676,0.2458471287
```

{85} AG_B

```
Charge = 0 Multiplicity = 1
 N,0,-1.7987343346,0.1121860392,0.1126460421
 H,0,-2.1729518457,-0.1643587059,1.0148517934
 H,0,-2.5092961613,-0.0693211915,-0.5852784915
 N,0,-0.6642512732,-0.6448141806,-0.19115797
 C,0,0.5856878065,-0.0780974696,-0.0289725074
 N,0,1.606105758,-0.8643412579,0.0917519139
 N,0,0.6134976627,1.2867014432,-0.113114896
 H,0,1.4288204047,1.7263122349,0.282401736
 H,0,-0.2651006834,1.7429015534,0.0879110104
 H,0,2.4535264177,-0.3051184255,0.1148813861
 H,0,-0.6868017514,-1.6238830396,0.0599789829
```

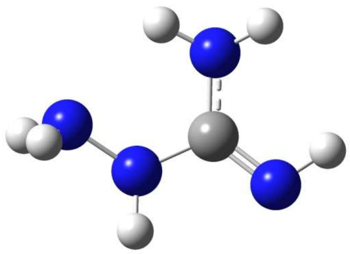

{86} AG_C

```
Charge = 0 Multiplicity = 1
 N,0,-1.7944358785,0.0754541235,0.0928035693
 H,0,-2.1868665652,-0.1996099418,0.9873714806
 H,0,-2.4776489824,-0.135758399,-0.6242538603
 N,0,-0.6287676303,-0.6506976282,-0.164206504
 C,0,0.6030744705,-0.0388197837,-0.0278925915
 N,0,1.719676109,-0.6868188565,0.0663805611
 N,0,0.5792398102,1.3247900026,-0.1057501873
 H,0,1.3992846386,1.7800780394,0.2595693052
 H,0,-0.303607857,1.7508774261,0.1333241397
 H,0,1.5252363258,-1.6844579535,0.0815167435
 H,0,-0.6343074407,-1.6291670291,0.0913723436
```

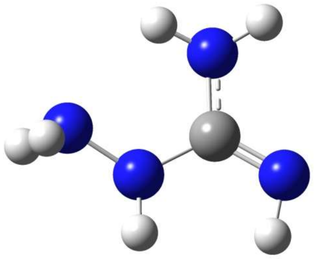

{87} AG_D

```
Charge = 0 Multiplicity = 1
 C,0,-0.506095489,0.1220613005,-0.0120571794
 N,0,0.6881725629,-0.558049556,-0.1030855864
 N,0,1.8972681979,0.1195469342,0.0309539592
 N,0,-0.5801689448,1.4095510217,-0.0327642233
 N,0,-1.5833030443,-0.7360492234,0.1261238479
 H,0,-2.4647134969,-0.3298073653,-0.1481545736
 H,0,-1.4585255775,-1.6642772817,-0.2524295138
 H,0,0.6674604819,-1.5122344587,0.2309693211
 H,0,2.5626206935,-0.2878842635,-0.6137245439
 H,0,-1.5610607694,1.6722294862,-0.0074410442
 H,0,2.2618503855,-0.003345594,0.9703365366
```

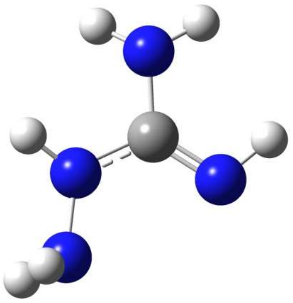

{88} $(AG_A)_2$

```
Charge = 0 Multiplicity = 1
 N,0,-4.020652936,-1.748896825,0.3190639115
 H,0,-4.4500193697,-2.1191132734,-0.5228365237
 H,0,-4.7445630171,-1.6475462697,1.0192030261
 N,0,-3.4617027667,-0.4974637428,0.0659026823
 C,0,-2.0890327305,-0.4221790266,-0.0937323029
 N,0,-1.2573826978,-1.3606428701,0.2210223033
 N,0,-1.659140806,0.7626460395,-0.627906569
```

H,0,-0.6825227584,0.9935778536,-0.4354344087
H,0,-2.2940119831,1.5421063587,-0.5480221323
H,0,-1.8047530723,-2.1580043518,0.5351154072
H,0,-4.0056341463,0.1343986784,-0.5076301925
N,0,4.049024227,1.7109298464,-0.127142485
H,0,4.4523229376,1.9133105167,-1.0363546326
H,0,4.79381376,1.7420129009,0.557585909
N,0,3.4867016069,0.4353389813,-0.1215295984
C,0,2.1101744225,0.3353120206,-0.2264163245
N,0,1.2852616808,1.3186099701,-0.0724572133
N,0,1.668429218,-0.92809287,-0.5144220093
H,0,0.6984917822,-1.115678578,-0.2532032455
H,0,2.3074137564,-1.6799436647,-0.3059232277
H,0,1.8386079806,2.1597621191,0.0698480681
H,0,4.0153586818,-0.2943307232,-0.5822195723

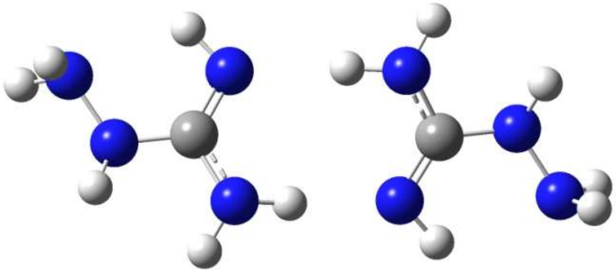

{89} $(AG_A)_3$

Charge = 0 Multiplicity = 1
N,0,5.0037325086,1.6439898157,-3.2623713863
H,0,5.214950295,1.0708849587,-2.4512165353
H,0,4.977234302,1.0412040206,-4.0747949544
N,0,5.9918960547,2.6082859236,-3.4480627876
C,0,5.7218561705,3.8935652868,-3.0132761192
N,0,4.5662252298,4.3407573563,-2.663154516
N,0,6.8399141995,4.696193052,-2.9779330253
H,0,6.6224780464,5.6803618691,-2.9776289233
H,0,7.5785063968,4.4563013809,-3.6222850654
H,0,3.9172330374,3.5573393747,-2.6929907405
H,0,6.9500872682,2.3030107491,-3.3339936427
N,0,3.4319591928,-3.8883079032,0.5183092333
H,0,4.2958874256,-4.2807132686,0.8702883563
H,0,2.9528484147,-4.6094116157,-0.0117040984
N,0,3.7348116291,-2.8042309902,-0.3039306035
C,0,2.9196263292,-1.6873421533,-0.2269294725
N,0,2.0558467646,-1.4657813856,0.7085246663
N,0,3.101707253,-0.8039592903,-1.2572826935
H,0,2.7843327003,0.1499385638,-1.0692901979
H,0,3.9942777892,-0.851000743,-1.7261188947
H,0,2.0502608719,-2.2805317369,1.3172449966
H,0,4.0787921488,-3.0236459636,-1.2300562066
N,0,1.2304010008,4.5823146358,-0.060981677
H,0,1.5202039338,5.3269533378,0.5601372813
H,0,0.4454485889,4.9185494378,-0.6093990077
N,0,0.8499583704,3.4805769267,0.7027674868
C,0,1.2317797163,2.2252906772,0.2628242399
N,0,2.1142150261,2.0018484558,-0.654617468
N,0,0.581621483,1.1972259564,0.8926850183
H,0,1.062429921,0.2965081539,0.8447417287
H,0,0.1995023716,1.4101891847,1.8016146934
H,0,2.4109977033,2.9021900532,-1.0229904594
H,0,-0.0554101134,3.5303889999,1.1523322642

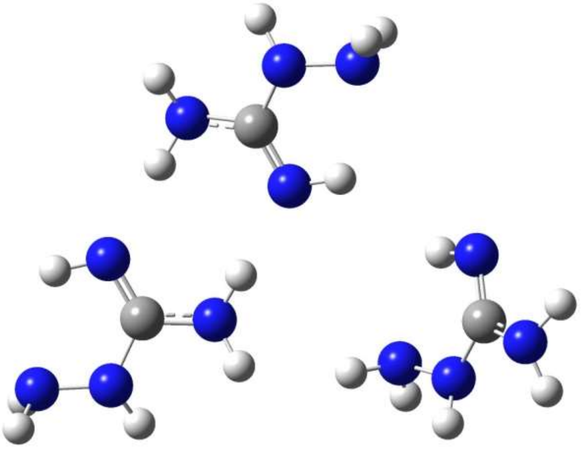

{90} ASC^-

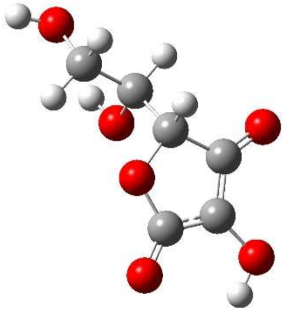

Charge = -1 Multiplicity = 1
C,0,-2.0113725502,0.2411115215,0.5585246119
O,0,-2.6529667905,1.1933390879,-0.1957674898
C,0,-1.6691612519,2.0820058343,-0.7429547457
C,0,-0.3252085275,1.5548095183,-0.2661404315
C,0,-0.6184052391,0.456016813,0.5170648657

```
O,0,0.3076571947,-0.3212725445,1.1803235569
H,0,-0.1497244448,-1.0728175586,1.5727606118
O,0,0.7715858386,2.0942927076,-0.5669270273
O,0,-2.667526667,-0.6237909288,1.1398780469
H,0,-1.8391872506,3.0733156671,-0.311370911
C,0,-1.7957909299,2.188120694,-2.2495130571
H,0,-1.0087030282,2.8839412785,-2.5719356212
C,0,-3.1326084813,2.7580428528,-2.6731837661
H,0,-3.9429244382,2.083637927,-2.3792120803
H,0,-3.2843869098,3.7267472992,-2.1831808379
O,0,-3.1107210044,2.9108909838,-4.0868750808
H,0,-4.0142124402,3.0158908515,-4.3935843144
O,0,-1.5694960487,0.9121976054,-2.826763254
H,0,-1.7310730409,0.99214091,-3.7716127662
```

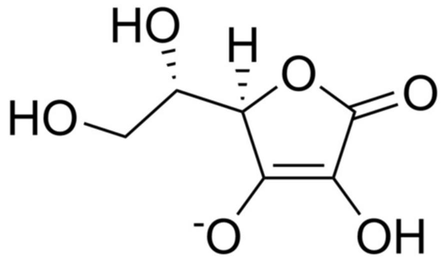

Supplement: Supplementary file 1 [file antioxidants-09-00756-s001.pdf]
